# Recent Progress of Hollow Carbon Nanocages: General Design Fundamentals and Diversified Electrochemical Applications

**DOI:** 10.1002/advs.202206605

**Published:** 2023-01-01

**Authors:** Zesheng Li, Bolin Li, Changlin Yu, Hongqiang Wang, Qingyu Li

**Affiliations:** ^1^ College of Chemistry Guangdong University of Petrochemical Technology Maoming 525000 China; ^2^ Guangxi Key Laboratory of Low Carbon Energy Materials Guangxi Normal University Guilin 541004 China

**Keywords:** composite nanocages, electrocatalytic conversion, electrochemical energy storage, hollow carbon nanocages, hollow carbon spheres, single atom catalysts

## Abstract

Hollow carbon nanocages (HCNCs) consisting of sp^2^ carbon shells featured by a hollow interior cavity with defective microchannels (or customized mesopores) across the carbon shells, high specific surface area, and tunable electronic structure, are quilt different from the other nanocarbons such as carbon nanotubes and graphene. These structural and morphological characteristics make HCNCs a new platform for advanced electrochemical energy storage and conversion. This review focuses on the controllable preparation, structural regulation, and modification of HCNCs, as well as their electrochemical functions and applications as energy storage materials and electrocatalytic conversion materials. The metal single atoms‐functionalized structures and electrochemical properties of HCNCs are summarized systematically and deeply. The research challenges and trends are also envisaged for deepening and extending the study and application of this hollow carbon material. The development of multifunctional carbon‐based composite nanocages provides a new idea and method for improving the energy density, power density, and volume performance of electrochemical energy storage and conversion devices.

## Graphical abstract

In this review, a clear and comprehensive definition of hollow carbon nanocages (HCNCs) is provided. The latest research progress (including preparation, regulation, and modification) of HCNCs in the field of electrochemical energy storages and conversions is detailly reviewed and discussed. The challenges and some insights into new trends and directions for HCNCs are also provided.

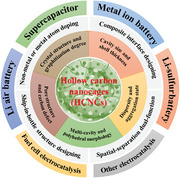

## 1 Introduction

In the past several decades, 0D fullerenes (1985), 1D carbon nanotubes (CNTs) (1991), 2D graphene (2004), and graphdiyne (2010) have been discovered successively, making carbon nanomaterials one of the most interesting frontiers today.^[^ ^1^, ^2^, ^3^, ^4^, ^5^ ^]^ In the last decade, scientists all over the world have been devoted to the physical and chemical research of various carbon nanomaterials, and have accumulated long‐term achievements in the design and energy‐related application of new carbon nanomaterials, especially in the mesoscopic nanostructures of 3D carbon nanomaterials (such as 3D carbon nanotube arrays, 3D graphene networks, 3D carbon nanocages).^[^ ^6^, ^7^, ^8^ ^]^ Carbon nanomaterials have been the ideal choice for electrochemical energy storage and catalytic materials because of their diverse structures, rich surface states, high controllability, and good chemical stability, as well as excellent electrical transport characteristics and high active surface.^[^ ^9^, ^10^ ^]^ Whether 0D fullerenes, 1D CNTs, 2D graphene, or 3D graphene networks, carbon nanomaterials attract the world's attention. Especially in recent years, the rapid development of 3D carbon nanomaterials has provided new opportunities for their applications in field of electrochemistry (including electrochemical energy storages and conversions), because of their unique structures and excellent performances.^[^ ^11^, ^12^ ^]^

Hollow carbon nanocages (HCNCs) (including hollow carbon spheres and polyhedras) are a new kind of 3D nanostructured carbon material composed of curved carbon nanosheets with sub‐micrometer gap space, and the carbon nanosheets are interconnected by graphene microcrystal as construction units.^[^ ^13^, ^14^, ^15^ ^]^ There are abundant sub‐nanochannels (≈1 nm) or specially designed nanopores (1–10 nm) connecting inside and outside on the shells of carbon nanocages. Different from other nanocarbon materials, this kind of new carbon nanocages material has integrated characteristics of available inner cavity, coexistence of micropores, mesopores, and macropores, high specific surface area, easy doping, and modulation.^[^ ^16^, ^17^, ^18^, ^19^, ^20^ ^]^ Furthermore, the curved nanosheets structure of cage‐like carbon nanomaterials could effectively reduce anisotropy due to the arch configuration of carbon layers, which can avoid interlayer slipping and ensure structural stability.^[^ ^21^, ^22^ ^]^ Therefore, the HCNCs with favorable dimensional structures and porous structures can greatly promote the exchange and transfer of substances in the liquid–solid and gas–solid electrocatalytic reaction, and effectively accommodate the strain relaxation during energy storage, which has become a new platform for developing advanced energy conversion and storage functions.

**Figure 1:**
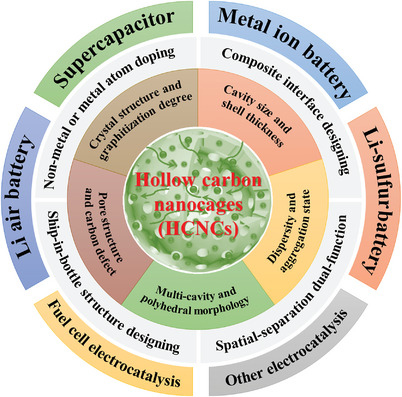
Schematic diagram for the core content of this review.

In this review, we provide a clear and comprehensive definition of HCNCs. Carbon nanocages are hollow carbon nanomaterials with unique hollow internal structures (including ship in a bottle structure), adjustable structural parameters (graphitization degree, cage size, shell thickness, shell pore structure, element composition, etc.), and diversified nanomorphology (such as hollow cubes, hollow polyhedrons, hollow nano‐ or microspheres, or even irregular morphology). The research progress (including preparation, regulation, and modification) of HCNCs in the field of electrochemical energy storages and conversions is detailly reviewed. The up‐to‐date preparation strategies (such as template preparation methods) of HCNCs are introduced in detail. In particular, the structural regulation and modification principles of HCNCs and the methods to improve their performances are discussed in depth. The structural regulations of HCNCs include the following five aspects: 1) crystal structure and graphitization degree regulation, 2) cavity size and shell thickness regulation, 3) pore structure and carbon defect regulation, 4) dispersity and aggregation state regulation, and 5) multicavity and polyhedral morphology regulation. The structural modifications of HCNCs also include five aspects: 1) nonmetal heteroatom doping, 2) metal single/dual atom doping, 3) composite interface designing, 4) ship‐in‐bottle structure designing, and 5) spatial‐separation dual‐function designing modifications (see **Figure** **1** for details). Finally, the remaining challenges are summarized, and some insights into new trends and directions for HCNCs are provided. This review will provide new insights for understanding HCNCs and help researchers in related fields to have a deeper and more comprehensive understanding of HCNCs in advanced electrochemical energy storages (supercapacitors, metal‐ion batteries, metal–air batteries, metal–sulfur batteries) and conversions (fuel cell electrocatalysis and other electrocatalysis).

## 2 Preparation Methods of Hollow Carbon Nanocages

Because of the special hollow structure and its unique physical and chemical properties, hollow carbon materials have been widely concerned by researchers in many fields. However, it is still very challenging to accurately design and control the synthesis of hollow porous carbon nanomaterials. The HCNCs with controlled structure and porosity have been facially synthesized by a series of template‐based methods and some nontemplate methods. In this review, we focus on the principles and cases of template preparation methods (especially hard‐template method) of HCNCs.

### 2.1 Template‐Based Preparation Strategy

In the process of preparing HCNCs, the template method has attracted great attention of scientists due to its advantages of precise regulation, simple operation, and low cost. There are two types of template methods: hard template method and soft template method. Because of the co‐self‐assembly of carbon precursors and decomposing amphiphilic molecules, the soft template process is quite easy to fabricate hollow structures, but the morphology of product is relatively poor. Hard templates are usually formed by coating spherical templates with carbon precursors, followed by carbonization, and finally etching templates with acid or organic solvents to synthesize uniform HCNCs. Therefore, the hard template method is a simple and effective method to prepare HCNCs. This review mainly focuses on hard template, and summarizes its principle and typical preparation cases.

**Figure 2:**
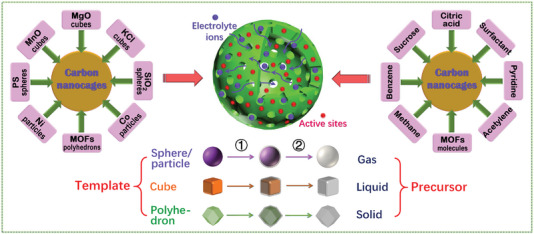
Schematic diagram for formation of hollow carbon nanocages (HCNCs) (including hollow carbon spheres, cubes and other polyhedrons) with diversified templates and precursors (step (1) is in situ carbon growth and step (2) is template removing). The small red dots on the HCNCs indicate the active sites (both metallic and nonmetallic active sites). The blue dots represent electrolyte ions.

Over the years, a variety of hard templates have been selected for the design and synthesis of versatile HCNCs in the fields of electrochemical energy storage and conversion. Among them, sphere/particle, cube, and polyhedron templates are the three important categories for the construction of homologous carbon nanocages, where the schematic diagram is vividly depicted in **Figure** **2**. The intrinsic micropores or customized mesopores of HCNCs jointly promote the mass exchange and ion conduction inside and outside the HCNCs. The sp^2^ hybrid carbon layer of HCNCs provides a good basis for electron conduction toward diversified electrochemical applications. Currently, cube‐like carbon nanocages are a kind of promising carbon material. For example, Xie and co‐workers developed an in situ MgO template method to produce the HCNCs with unique cube‐like morphology,^[^ ^23^ ^]^ which have been extended into several technical fields such as oxygen reduction electrocatalysis,^[^ ^24^ ^]^ lithium–sulfur battery,^[^ ^25^ ^]^ supercapacitor,^[^ ^26^ ^]^ and so on. Chen et al. also reported the preparation of porous HCNCs with mesoporous MnO nanocubes as template,^[^ ^27^ ^]^ which was successfully applied to the design of Sn–C nanocomposites as anodes of sodium‐ion batteries with outstanding electrochemical performances. More interestingly, a simple one‐step templating technique based on KCl nanocubes was developed recently for the preparation of novel 3D HCNCs supported on ultrathin carbon nanosheets as anodes of lithium‐ion batteries.^[^ ^28^ ^]^ In addition to cube‐like HCNCs, the polyhedral HCNCs have attracted intensive attention in a variety of electrochemical applications. Especially, the polyhedral metal–organic frameworks (MOFs, e.g., ZIF‐8 and ZIF‐67 polyhedrons)‐derived hybrid carbon nanocages with foreign components (e.g., Co, Co–Zn, Pt–Co, NiCoP, etc.) have been developed for the applications of lithium–sulfur batteries, oxygen reduction, and oxygen evolution electrocatalysis.^[^ ^29^, ^30^, ^31^, ^32^ ^]^ Because of its unique curved surface and sphere geometry, the spherical HCNCs demonstrated more merits and popularization value than other carbon nanocages, including well fluidity, high packing density, and excellent processability.^[^ ^33^, ^34^, ^35^ ^]^ Originally, the spherical HCNCs were designed and synthesized through polystyrene (PS)^[^ ^36^ ^]^ and silicon dioxide (SiO_2_)^[^ ^37^ ^]^ spherical templates. Although the sphericity of these nanocages is well structured, their degree of graphitization is relatively low, resulting in poor electrical conductivity. For this reason, transition metal nanoparticles have been recently proposed as templates and catalysts for constructing graphitic (or graphene‐like) HCNCs. For example, Li and co‐workers reported the large‐scale synthesis of hollow graphene‐like nanocages for supercapacitor application via an efficient template‐directed catalytic growth using Ni nanoparticles.^[^ ^38^ ^]^ Liu et al. also reported the fabrication of a class of novel network‐like graphitic carbon nanocages, which result from the catalytic graphitization of amorphous carbon by the in situ obtained Co nanoparticles.^[^ ^39^ ^]^ However, most of the previous works generally focus on solving the problems of graphitization regulation by transition metal catalysis. There are rare reports of simultaneously improving the degree of sphericity and electrochemical performance by heterogeneous element doping.

**Figure 3:**
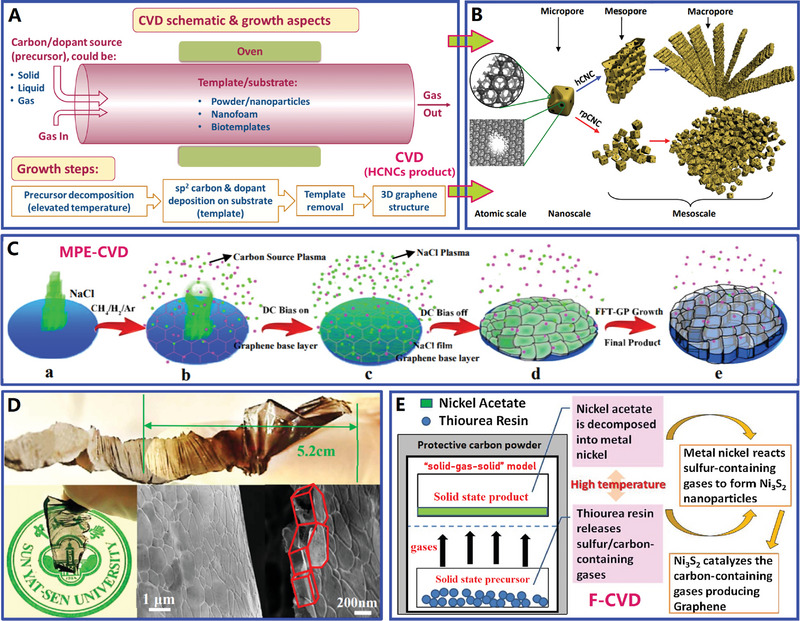
A) Schematic diagram of the device for chemical vapor deposition (CVD). Reproduced with permission.^[^ ^6^ ^]^ Copyright 2019, Wiley. B) Multiscale HCNCs products synthesized by CVD. Reproduced with permission.^[^ ^25^ ^]^ Copyright 2015, Elsevier. C,D) Macroscopical HCNCs products synthesized by MPE‐CVD. Reproduced with permission.^[^ ^40^ ^]^ Copyright 2015, ACS. E) Basic principles and processes of F‐CVD. Reproduced with permission.^[^ ^46^ ^]^ Copyright 2017, Elsevier.

On the basis of the different synthetic strategies (e.g., chemical vapor deposition, liquid‐phase and solid‐phase synthesis), the precursors of HCNCs mainly include three types: gas‐phase, liquid‐phase, and solid‐phase precursors (see Figure 2). Numerous gas‐phase precursors including benzene vapor,^[^ ^23^, ^24^, ^25^, ^26^ ^]^ acetylene,^[^ ^27^ ^]^ methane,^[^ ^40^ ^]^ and carbon monoxide,^[^ ^41^ ^]^ have been used for preparing carbon nanocages by rigorous chemical vapor deposition (CVD) technique (see **Figure** **3**A,B). Among them, benzene vapor becomes an excellent carbon precursor for the controllable preparation of ultrathin HCNCs by minimized benzene dosage upon MgO template.^[^ ^23^, ^24^, ^25^, ^26^ ^]^ Acetylene was also utilized as a feasible carbon precursor for the growth of graphitic porous HCNCs on MnO template.^[^ ^27^ ^]^ While methane and carbon monoxide are universal gas‐phase precursors for the construction of diversified carbon nanomaterials by CVD techniques.^[^ ^42^ ^]^ For example, Wang and co‐workers presented the development of freestanding and transparent graphene polyhedron HCNCs, which was fabricated by using NaCl template from methane precursor in a microwave‐plasma‐enhanced chemical vapor deposition (MPE‐CVD) system^[^ ^40^ ^]^ (see Figure 3C,D). Recently, Jiang et al. demonstrated that graphitized HCNCs can be prepared with carbon dioxide and magnesium metal as precursors, where the combustion reaction of carbon dioxide and magnesium produced MgO template and CO carbon source.^[^ ^41^ ^]^ On the other hand, liquid‐phase (e.g., pyridine^[^ ^43^ ^]^) and solid‐phase (e.g., sucrose,^[^ ^44^ ^]^ citric acid,^[^ ^45^ ^]^ surfactant,^[^ ^28^ ^]^ and MOFs^[^ ^29^ ^]^) precursors are often chosen for the catalytic preparation of graphitized HCNCs during liquid‐phase and/or solid‐phase synthesis. Relative to the gas‐phase precursors, the solid‐phase precursors have several advantages such as high safety, low cost, and convenience in storage and use, however, there is a big drawback, poor dispersity with templates and catalysts. More recently, a high‐efficiency facile‐CVD (F‐CVD) growth technique (or called quasi‐CVD (Q‐CVD) growth technique) was developed for one‐pot construction of 3D graphene nanosheets/Ni_3_S_2_ nanoparticles composite with thiourea resin as carbon and sulfur sources^[^ ^46^ ^]^ (see Figure 3E). Different from the traditional CVD method, the facile‐CVD growth follows the “solid–gas–solid” mass conversion model, that is to say, the precursor is initially a solid‐phase form and then transformed into gas‐phase form at elevated temperature, finally, solid‐phase carbon layer was deposited on catalyst/template materials.^[^ ^47^ ^]^ With all this in mind, the facile‐CVD growth technique combines the advantages of traditional CVD synthesis and high‐temperature solid‐phase synthesis (see Figure 3E for details): i) high dimensional controllability, ii) convenient compositional regulation, and iii) excellent cost effectiveness. Regarding the construction of functionalized HCNCs, the facile‐CVD synthesis with “solid–gas–solid” model will become a high‐efficiency and scalable production technique, which is superior to previous synthesis methods of carbon nanocages.^[^ ^38^, ^46^ ^]^

On the other hand, soft template method is also a common method to fabricate hollow carbon nanostructures. The soft template materials involved include emulsion droplets, vesicles/micelles, bubbles, etc.^[^ ^48^ ^]^ With this approach, the morphology consistency of HCNCs product is sometimes compromised. However, by acting on the hollow interior with a functional substance, the possibility of producing more complex layered structures is greatly increased.^[^ ^49^ ^]^ Compared with the hard template method, the soft template method also has some advantages. In the experiment process, the steps of template synthesis and template removal are avoided, and the introduction of impurities is avoided. The soft template method can be used to prepare hollow nanomaterials by hydrothermal, sol–gel or emulsion polymerization.

### 2.2 Template‐Free Preparation Strategy

With the rapid development of nanotechnology, template‐free (or self‐template) method has become a new way to synthesize hollow nanostructures. Unlike the traditional hard/soft template approach, the precursor of the template‐free (or self‐template) approach is not only used as a template to produce a hollow interior, but is also transformed into the basic components of the final product. In general, the self‐template method involves two typical processes: 1) the formation of self‐template materials (solid nanostructures), and 2) the in situ transformation into hollow nanostructures.^[^ ^50^ ^]^ Hollow carbon‐based nanomaterials show great potential in research fields such as electrochemical devices for energy conversion or storage. Currently, the synthesis methods of HCNCs mainly rely on template‐based strategy, which are conceptually easy to form hollow structures, but face challenges such as time consuming, low product yields, and environmental problems caused by harmful etching agents. Therefore, a new strategy to prepare HCNCs without templates is desirable, not only to ensure accurate control of key structural parameters of hollow structures with specified functions, but also to be an environmentally friendly and scalable approach suitable for practical applications.^[^ ^51^ ^]^

## 3 Structural Regulations of Hollow Carbon Nanocages

HCNCs have attracted great attention due to their special structure and attractive application prospects. However, the previous work mainly focused on the preparation of hollow structural units.^[^ ^18^, ^19^, ^20^ ^]^ However, there are few studies on the cavity size, shell thickness, pore structure, and aggregation state of HCNCs. We will summarize the following five aspects as structural regulations of HCNCs: 1) crystal structure and graphitization degree regulation, 2) cavity size and shell thickness regulation, 3) pore structure and carbon defect regulation, 4) dispersity and aggregation state regulation, and 5) multicavity and polyhedral morphology regulation.

### 3.1 Crystal Structure and Graphitization Degree Regulations

Graphitization degree refers to the degree that the crystal structure of carbon material is close to that of graphite. The increase of graphitization degree means that the crystal structure of carbon material is closer to graphite, which can be reflected by the intensity of (002) peak on XRD spectrum (the stronger (002) peak, the higher graphitization degree) and interplanar spacing of (002) crystal face by TEM observation (the narrower (002) face spacing, the better crystal structure).^[^ ^7^ ^]^ The increase of graphitization degree of carbon materials will generally increase the electronic conductivity and chemical stability of carbon materials, which are two key parameters for electrochemical applications.^[^ ^52^ ^]^ In the process of graphitization, the orderly transformation of thermodynamically unstable amorphous carbon from disordered layer structure to graphite crystal structure is necessarily occurred. Therefore, high temperature heat treatment (generally more than 2000 °C) or catalytic graphitization (with Fe, Co, and Ni metals or compounds as catalysts) should be used to provide energy or reduce activation energy for the carbon atomic rearrangement and structural transformation.^[^ ^53^ ^]^

According to the difference in crystal structure and graphitization degree, hollow carbon nanocages can be divided into three types: 1) amorphous carbon nanocages, 2) graphitized carbon nanocages, and 3) graphene‐like carbon nanocages. The amorphous carbon nanocages can be widely prepared by using nonmetallic templates (e.g., PS spheres^[^ ^36^ ^]^ and SiO_2_ spheres^[^ ^37^ ^]^) or template‐free method,^[^ ^51^ ^]^ and the resulting products are usually low‐crystalline carbon nanocages due to the noncatalytic carbon deposition action and lower carbonation temperature (500–900 °C). Usually, the SiO_2_‐assisted pyrolysis of solid‐state carbon precursor (glucose, dopamine, resorcinol–formaldehyde (RF) resin, etc.) at temperature of 500–900 °C, can generate amorphous carbon nanocages.^[^ ^54^, ^55^ ^]^ However, the SiO_2_‐assisted CVD method with gaseous carbon precursor (benzene^[^ ^56^ ^]^ and methane^[^ ^57^, ^58^ ^]^) at temperature of 1000–1200 °C can generate graphited carbon nanocages. Typically, the carbon shell consists of >10 graphene layers (>5 nm) after 5 min of metal‐free CVD growth at 1150 °C,^[^ ^57^ ^]^ with the graphitization degree comparable to that of multiwall carbon nanotubes.

Furthermore, the porous graphited carbon nanocages can be prepared by volatile metal precursor (ferrocene)‐assisted CVD method, where SiO_2_ spheres with solid core and mesoporous shell were used as template and ferrocene was employed as the carbon precursor (also release Fe catalyst).^[^ ^59^, ^60^ ^]^ During the CVD process (550 °C), Fe and carbon are embedded in the pores of a SiO_2_ template, facilitating the synchronous synthesis of highly ordered graphited structures with porous textural property by continuous carbonization (800–1000 °C). Typically, the sphere diameter is ≈280 nm and the shell thickness is ≈40 nm (porous graphited carbon shell) after 90 min of Fe‐catalysis CVD growth at 850 °C.^[^ ^59^ ^]^ Analogous porous graphited carbon nanocages can also be prepared by the two‐step synthesis strategy, namely, carbonization of porous carbon nanocages at 650 °C and subsequent Fe catalytic graphitization at 1000 °C (with FeCl_3_ as a catalyst precursor).^[^ ^61^ ^]^

**Figure 4:**
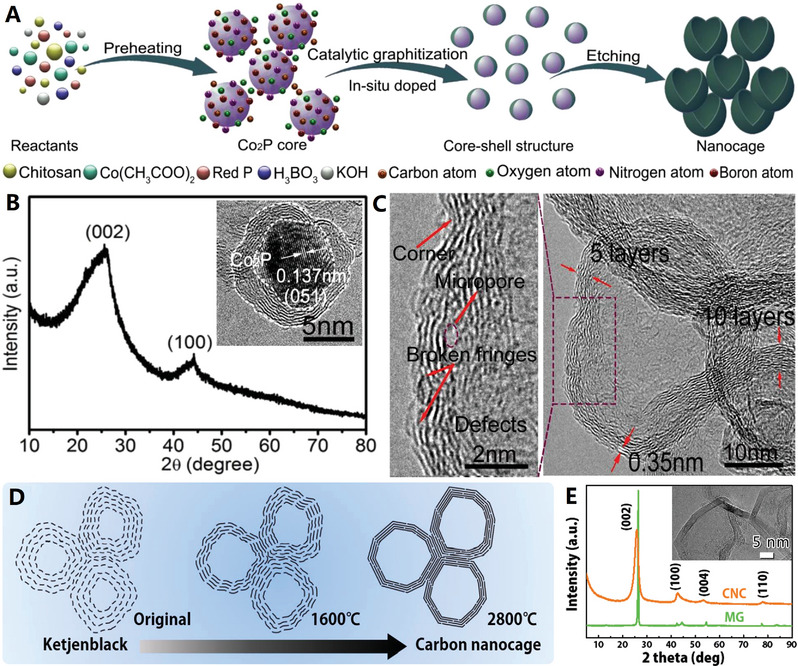
A–C) The defect‐rich ordered graphitic structure of graphene‐like carbon nanocages. Reproduced with permission.^[^ ^66^ ^]^ Copyright 2017, Elsevier. D,E) The high‐crystalline ordered graphitic structure of graphene‐like carbon nanocages. Reproduced with permission.^[^ ^22^ ^]^ Copyright 2018, Wiley.

The graphene‐like carbon nanocages can be controllably prepared by using metallic templates (e.g., single metals,^[^ ^62^, ^63^ ^]^ metal alloys,^[^ ^64^, ^65^ ^]^ phosphides,^[^ ^66^, ^67^ ^]^ sulfides,^[^ ^68^, ^69^ ^]^ and carbides^[^ ^70^, ^71^ ^]^) at relatively low temperature (500–1000 °C) from diversified carbon precursors (ranging from carbohydrates to polymers and organic molecules). Typically, these graphene‐like carbon nanocages have sectional graphitized structures (i.e., microcrystalline graphene layers) and ultrathin carbon shells (1–10 layers of graphene and less than 5 nm in thickness) by controlled catalytic graphitization with metallic nanoparticles.^[^ ^62^, ^63^, ^64^, ^65^, ^66^, ^67^, ^68^, ^69^, ^70^, ^71^ ^]^ Due to their strong catalytic effect, the single metal (e.g., Fe, Co, and Ni) and their alloys nanoparticles have been earlier used for in situ growth of graphene‐like carbon nanocages with very precise pyrolysis parameters.^[^ ^62^, ^63^, ^64^, ^65^ ^]^ Recently, a variety of transition metal compound nanoparticles (e.g., phosphides, sulfides, and carbides) have been proved to be effective for catalytic growth of graphene‐like carbon nanocages.^[^ ^66^, ^67^, ^68^, ^69^, ^70^, ^71^ ^]^ For example, graphene‐like carbon nanocages in defect‐rich characteristic is designed and synthesized by pyrolysis assisted in situ Co_2_P catalytic graphitization method at a mild temperature of 750 °C^[^ ^66^ ^]^ (see **Figure** **4**A–C for details). This product has relatively wide (002) peak due to the ultrathin graphite shell (≈5 nm) and interrupted graphene microcrystals (with rich corners) deposited on Co2P nanoparticles (see Figure 4B). This newly graphene‐like carbon nanocage consists of 5–10 graphene layers (with 0.35 nm spacing of (002) face), which also show abundant carbon defects, broken fringes, and micropores (see Figure 4C). The unique semiopen nanocage structure, the defect‐rich ordered graphitic structure as well as the doping structure jointly contribute to the excellent electrocatalytic activity of the graphene‐like carbon nanocage material by providing abundant active sites and high‐efficiency charge transport.^[^ ^66^ ^]^

The catalytic preparation of graphene‐like carbon nanocages by above‐mentioned methods usually require a strict acid etching treatment (e.g., HCl, H_2_SO_4_, HNO_3_ or HF) to remove the metal core for hollow architectures, resulting their tough scaled‐up applications and slow industrialization process.^[^ ^72^ ^]^ High‐temperature conversion of carbon black nanoparticles into hollow graphitized counterparts is an efficient method for mass‐preparation of graphene‐like carbon nanocages. Recently, the interconnected graphene‐like carbon nanocages were prepared by high‐temperature treatment of Ketjen carbon black (EC300J) at 2800 °C under Ar atmosphere (see Figure 4D,E).^[^ ^22^ ^]^ The original Ketjen carbon black is of low crystal structure with wide layer spacing (>0.36 nm), when the temperature reached 1600 °C layer spacing diminished sharply (≈0.35 nm). After 2800 °C conversion, the layer spacing further diminished (≈0.34 nm) and high‐crystalline graphene‐like carbon nanocages were formed (Figure 4D). This graphene‐like carbon nanocage has a highly graphitized layered structure with sharp (002) peak and an ultrathin shell thickness of ≈5 nm (Figure 4E). Meanwhile, Ketjen carbon black (EC600JD) has also been thermally converted into analogous graphene‐like carbon nanocages by a simple and rapid microwave pulse strategy.^[^ ^73^ ^]^ This high‐efficiency and catalyst‐free approach offers new opportunities to produce large‐scale, high‐quality graphene‐like carbon nanocages. Since carbon black is a commercially available material and the high‐temperature treatment (or microwave) is a mature technology, the production of graphene‐like carbon nanocages could be easily upgraded to industrial scale.^[^ ^22^, ^73^ ^]^

Because delocalized *π* bonds can be formed within the graphite layer, high graphitization of carbon nanocages usually implies excellent electronic conductance and charge transfer properties and low internal resistance. In the conventional carbonization process, high temperature (or metal catalysis) is conducive to the rearrangement of carbon atoms and the growth of graphite microcrystals, but too high temperature (or excessive catalysis) will lead to the reduction of pore volume and specific surface area.^[^ ^74^ ^]^ Therefore, suitable preparation temperature (or other preparation conditions) to balance graphitization, surface area, and pore volume are very important for carbon nanocages.

### 3.2 Cavity Size and Shell Thickness Regulations

**Figure 5:**
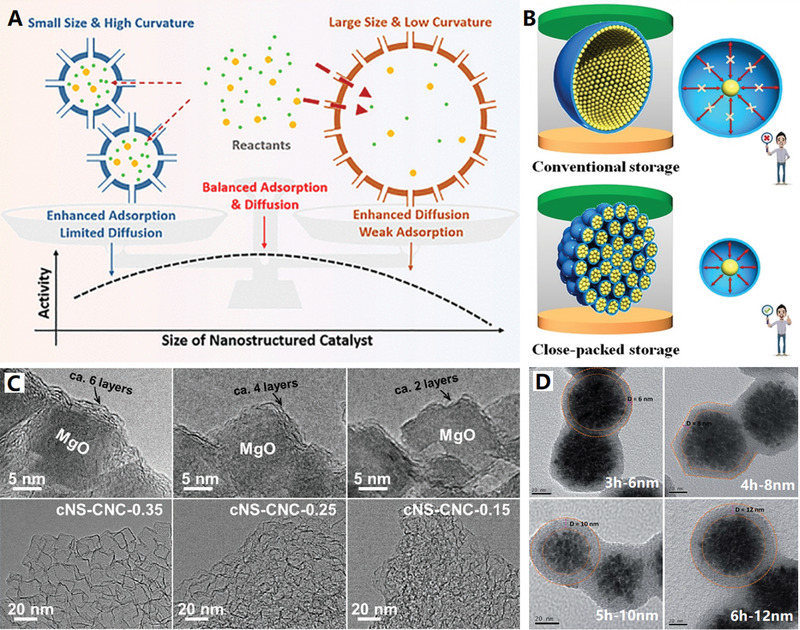
A) Balancing adsorption and diffusion for spherical carbon nanocages. Reproduced with permission.^[^ ^77^ ^]^ Copyright 2019, ACS. B) Close‐packed storage conception of ultrafine carbon nanocages array. Reproduced with permission.^[^ ^78^ ^]^ Copyright 2021, Wiley. C) Ultrathin collapsed carbon nanocages with different shell thickness. Reproduced with permission.^[^ ^88^ ^]^ Copyright 2020, Wiley. D) Carbon layer with different thickness on SnO_2_ nanoclusters template. Reproduced with permission.^[^ ^89^ ^]^ Copyright 2011, RSC.

As one of typical artificial nanoreactors, the metal species‐loaded hollow carbon nanocages show competitive potential in multifunctional catalysis due to its adjustable microenvironment and void‐confinement effect.^[^ ^75^ ^]^ The void‐confinement effect is one of the most basic functions to improve catalytic performance, which is a comprehensive effect including electron metal–support interaction (EMSI), enrichment and diffusion of reactants.^[^ ^76^ ^]^ The spherical curvature (i.e., cavity size) of carbon nanocages can affect the enrichment capacity of reactants, and the change of curvature can induce the balance effect between adsorption and diffusion of reactant molecules, that is, large curvature (small cavity size) will enhance adsorption but limit diffusion, while small curvature (large cavity size) will enhance diffusion but weaken adsorption (see **Figure** **5**A for details).^[^ ^77^ ^]^ So, only appropriate spherical curvature namely cavity size can offer the best enrichment capacity and generate the optimized catalytic performances by balancing adsorption and diffusion.

Hollow carbon nanocages have been widely used in electrochemical energy storage (such as supercapacitors and batteries) because of their porous carbon shells and large inner cavities. The inner cavity can adsorb electrolyte like a “reservoir” to meet the heavy demand for electrolyte ions during high‐current operation, so that it can maintain its high energy density under high power density.^[^ ^38^ ^]^ Therefore, the cavity size has also a direct impact on energy storage, where too small cavity is not conducive to ion diffusion and too large cavity will reduce volume energy density. The electrolyte ions can be bound by hollow carbon nanocages array composed of many ultrafine carbon nanocages, which can fundamentally improve the volume energy density via the close‐packed storage conception^[^ ^78^ ^]^ (see Figure 5B for details). With this unique close‐packed storage conception, the electrolyte ions can be better adsorbed and stably stored in ultrafine hollow carbon nanocages array to realize the efficient and intensive electrochemical energy storages.

At present, the cavity size of hollow carbon nanocages is mainly controlled by the size of the template (e.g., the size of PS and SiO_2_ spheres) or other reaction parameters (temperature and pressure). For example, using prefabricated SiO_2_ spheres with uniform diameter as template, carbon can be uniformly deposited on the outer surface of template by CVD or pyrolysis methods. Therefore, the cavity size of hollow carbon nanocages can be precisely controlled by the size of hard template (varying from 50 to 500 nm generally).^[^ ^79^ ^]^ And the sizes of SiO_2_ spheres can be easily turned by the hydrolysis rate (by altering the ammonia dosage or temperature) of tetrapropyl orthosilicate (TEOS) with the classic Stöber method.^[^ ^80^ ^]^ For metal or metal compound templates, the cavity size of hollow carbon nanocages can be controlled more finely (≈5 nm), through low temperature and rapid metal nucleation strategies.^[^ ^81^ ^]^ While as for template‐free strategy, the cavity size of hollow carbon nanocages is associated with multiple factors including precursor size, reactant concentration, and reaction temperature, etc.

On the other hand, the shell thickness of hollow amorphous carbon nanocages is closely related to the electrochemical performance and structural stability of the materials.^[^ ^82^ ^]^ The microporous or mesoporous structures in carbon shell can provide suitable habitat for other active components or directly carry out electrochemical ion adsorption. It has been realized that the synthesis with an adjustable thickness of carbon shell can effectively regulate the micro/mesoporous porosity and the adsorption capacity for foreign components or electrolyte ions.^[^ ^83^ ^]^ With respect to graphitized or graphene‐like carbon nanocages, the thinner shell thickness often gives the material more abundant active sites and better electrochemical activity.^[^ ^84^ ^]^ Furthermore, it is noteworthy that the shell thickness can significantly affect the structural stability of hollow carbon nanocages, when the shell thickness decreases, the shape of hollow nanocages is gradually changed from spherical geometry into hollow nanobowls^[^ ^85^ ^]^ or collapsed nanocages.^[^ ^86^ ^]^ Despite the structural deformation, these invaginated hollow nanocages can increase the stacking or tap density of powder materials.

Usually, the thickness of carbon shell can be well controlled by tuning the ratio of template and carbon source. For example, N‐doped hollow carbon spheres were controllably prepared by using Cu_2_O microspheres as a hard template and 3‐aminophenol formaldehyde resin polymer as carbon and nitrogen precursors.^[^ ^87^ ^]^ The thickness of the carbon shell can be easily controlled in the range of 15–84 nm by simply adjusting the weight ratios of the precursors to Cu_2_O microspheres. For the template‐assist CVD synthetic method, the dosage of precursor vapor can be used to control carbon shell thickness. Recently, an ultrathin N and S dual‐doped carbon nanocages with different shell thickness (≈6, 4, and 2 graphene layers) have been recently prepared upon the MgO cubic template, with gradually decreased dosage of the mixture of pyridine and thiophene (2:1, V/V) from 0.35 to 0.25, and 0.15 mL (see Figure 5C for details).^[^ ^88^ ^]^ After removing MgO template, the three samples collapsed to different degree via capillarity during drying, denoted as cNS‐CNC‐0.35, cNSCNC‐0.25, and cNS‐CNC‐0.15, respectively. In particular, the authors of the paper previously develop a novel method for preparing collapsed carbon nanocages by using capillary force compression, increasing volumetric energy density and power density for electrochemical energy storage by capillary force compression optimization (the capillary force compression can reduce excess macropores and mesoporous pores, that is a very effective way to increase the volume energy density).^[^ ^26^, ^88^ ^]^ For the hydrothermal synthetic method, the carbon shell thickness can be controlled by the hydrothermal deposition time. For example, the thickness of carbon layer on SnO_2_ nanoclusters template can be controlled in 6–12 nm at 3–6 h hydrothermal deposition time, by using glucose as carbon source and sodium stannate as template precursor (see Figure 5D for details).^[^ ^89^ ^]^ After removing the SnO_2_ template, new‐style hollow carbon nanocages with a built‐in carbon network structure can be synthesized, where the inner core and the outer shell of this cage‐like structure contain mesopores and micropores, respectively.^[^ ^90^ ^]^ Furthermore, the inner space and thickness of this hollow carbon nanocages can also be controlled by adjusting the molar ratio of glucose and sodium stannate.

### 3.3 Pore Structure and Carbon Defect Regulations

Based on the above introduction, we know that carbon shell thickness and porosity are interrelated. As a key structural element of hollow carbon nanocages, porosity has a crucial impact on physical confinement and charge storage.^[^ ^91^ ^]^ Therefore, the controllable adjustment of porous structure within carbon shell and in‐depth analysis of the relationship between porosity and electrochemical properties are very important to realize efficient electrochemical energy storages.^[^ ^92^ ^]^ By synchronously creating the porosity of carbon shell and utilizing the interior void volume of hollow carbon nanocages, sufficient space for charge/substance storage and fast pathways for electrolyte ion diffusion are well guaranteed.^[^ ^93^ ^]^ For the catalytic conversion, by adjusting the pore size and pore distribution on the carbon shell, specific reactants can preferentially enter the interior void to participate in the reaction and realize the selective catalysis.^[^ ^94^ ^]^ Moreover, the hollow carbon nanocages with porous thin wall construction can provide rich active sites and short mass transport path, so the catalytic activity can be greatly improved.^[^ ^95^ ^]^ According to the pore size, the pore structures can be divided into three types: micropores (<2 nm), mesopores (2–50 nm), and macropores (>50 nm).^[^ ^96^ ^]^ Micropores can afford high specific surface area and large porosity, but the ion diffusion and mass transfer are poor; mesopores and macropores have faster diffusion kinetics, but the specific surface area is relatively low.

For the pore structure of carbon nanocages, in the early stage, people paid the most attention to the ultrafine micropore structure (generally <1 nm) spontaneously generated during the high temperature preparation process (namely, the pyrolysis process of precursors).^[^ ^26^ ^]^ These ultrafine micropores are often difficult to detect in high‐resolution electron microscopes and need to be characterized in detail by nitrogen adsorption and desorption micropore analysis. These pyrolytically generated pores play a key role in connecting the internal and external environment of the carbon nanocages, which is equivalent to the bridging effect between the two sides of the carbon shell. These sub‐nanostructured micropores also contribute greatly to the high specific surface area and large pore volume of carbon nanocages. More recently, there has been more interest in the mesoporous structure of carbon nanocages, such as the design of mesoporous carbon hollow spheres with controllable pore sizes (ranging from 2 to 15 nm).^[^ ^26^, ^61^, ^88^ ^]^ These mesoporous designs are mainly focused on accelerated mass transfer (ionic conductivity) for electrochemical applications or as rational spaces for anchoring metal nanoparticles on to the mesoporous carbon nanocages.

According to the difference of pore structures, carbon nanocages can also be included three types: 1) microporous carbon nanocages, 2) mesoporous carbon nanocages, and 3) hierarchical porous carbon nanocages. The microporous carbon nanocages (hollow spheres) are widely synthesized using tetraethoxysilane (TEOS) as the silica precursor and resorcinol/formaldehyde (RF) polymer as the carbon precursor by one‐step assembly strategy (SiO_2_ spheres are formed via Stöber process,^[^ ^80^ ^]^ and SiO_2_@RF core/shell structure are formed synchronously with assistance of NH_4_^+^ ions), following by the carbonization and silica template removal.^[^ ^97^, ^98^, ^99^ ^]^ Normally, the shells of carbon nanocages by direct carbonization of polymer precursor (e.g., RF,^[^ ^97^, ^98^, ^99^ ^]^ polyaniline,^[^ ^100^ ^]^ polypyrrole,^[^ ^101^ ^]^ etc.) are of microporous structure due to the pyrolysis,^[^ ^102^ ^]^ hence the resulting products are regarded as microporous carbon nanocages. To further increase the pore size and tailor porosity of carbon shell (forming mesoporous or hierarchical porous structures), two synthetic routes have been demonstrated: 1) chemical activation (or oxidation etching) of the carbon shell, and 2) the incorporation of secondary nanotemplates (colloidal micelles or solid nanoparticles) into the carbon shell.

Generally, the pore size and pore volume of carbon nanocages can be effectively increased by KOH activation,^[^ ^103^, ^104^ ^]^ a well‐established approach to produce micro‐/mesoporosities in carbonaceous materials.^[^ ^105^, ^106^ ^]^ For example, the KOH‐activated SiO_2_‐templated hollow carbon nanospheres (forming mesoporous carbon nanocages) manifested high specific surface area (758 m^2^ g^−1^), high pore volume (1.72 cm^3^ g^−1^), and large mesoporous structure (20–40 nm in pore size).^[^ ^103^ ^]^ In addition, the KOH‐activated PS‐templated hollow carbon nanospheres (forming hierarchical porous carbon nanocages) also showed high specific surface area (923 m^2^ g^−1^), high pore volume (0.91 cm^3^ g^−1^), and hierarchical porous structure (1–2 and 8–15 nm in pore size).^[^ ^104^ ^]^ Thus, it can be seen that employing a hard‐template route associated with the chemical activation is an effective approach to prepare hollow carbon nanocages with increased the pore size and enhanced pore volume. Furthermore, mesoporous carbon nanospheres can be readily synthesized by an oxidation etching approach, using hydrogen peroxide (H_2_O_2_, 30 wt%) as etching agent.^[^ ^107^ ^]^ The typical formation procedure involves two steps: 1) fabrication of hollow carbon nanospheres with SiO_2_ hard template route, and 2) selectively tailoring of sp^2^ C—C and C=O covalent bonds by H_2_O_2_. The specific surface area (201–421 m^2^ g^−1^) and pore size (8–10 nm) can be easily controlled by adjusting the etching time, and thus the repeatability is quite high for this oxidation etching method.

**Figure 6:**
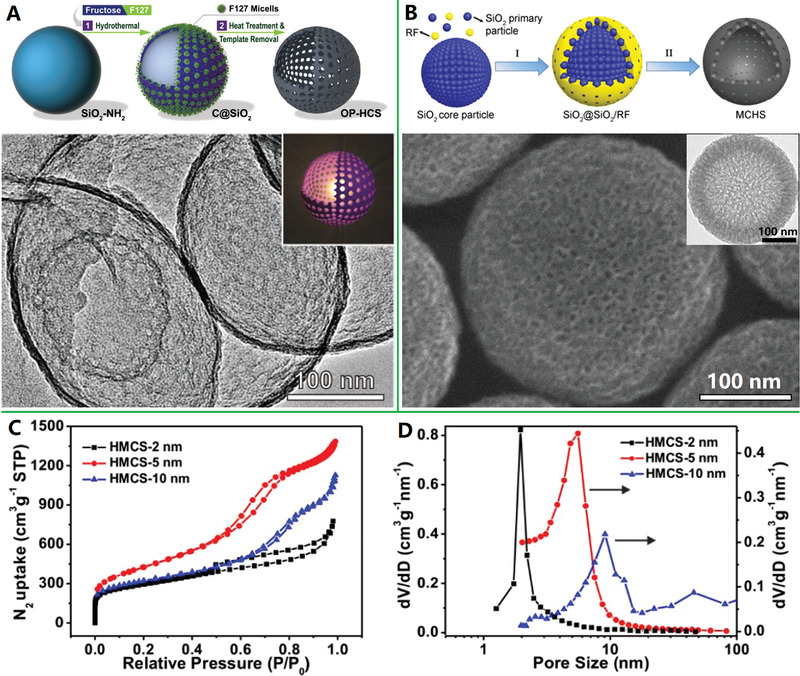
A) Micelles‐assisted SiO_2_ template synthesis of mesoporous carbon nanocages. Reproduced with permission.^[^ ^108^ ^]^ Copyright 2017, Wiley. B) SiO_2_@SiO_2_/RF template synthesis of mesoporous carbon nanocages. Reproduced with permission.^[^ ^111^ ^]^ Copyright 2016, ACS. C,D) The mesoporous carbon nanocages with pore size of 2, 5, and 10 nm. Reproduced with permission.^[^ ^112^ ^]^ Copyright 2020, Wiley.

Despite these progresses, the rational design of porous shell for carbon nanocages still remains challenges. First, the shell of above products displays uneven pore distribution and disordered pore arrangement, which are not conducive to the rapid mass/ions transport. Second, micropore (<2 nm in pore size) is still dominant in the carbon shell despite of formation of some mesopore, which results in a high innerpore transport resistance and a poor utilization of the pore surface area. The incorporation of secondary soft templates (F127 micelles^[^ ^108^, ^109^ ^]^ and PS‐*b*‐PEO micelles^[^ ^110^ ^]^) into the shell of carbon nanocages can generate ordered and uniform mesopores with controlled size. For example, hollow carbon nanocages with uniform diameters (≈300 nm) and ultrathin (≈5 nm), ordered mesoporous shells have been synthesized by a facile dual‐template (SiO_2_‐NH_2_ spheres and F127 micelles)‐assisted hydrothermal treatment and carbonization of fructose^[^ ^108^ ^]^ (see **Figure** **6**A). The ordered mesopores arrays with an average distance of 10–12 nm and a pore size of 4.37 nm are integrated into the shell of carbon nanocages, contributing to high specific surface area (805 m^2^ g^−1^) and high pore volume (0.90 cm^3^ g^−1^). Besides, the ordered mesoporous carbon nanocages with a large shell thickness of ≈36 nm and a uniform pore size distribution centered at 3.4 nm are also synthesized by self‐assembly of resol and F127 onto the surface of SiO_2_–CHO spheres and subsequent heat treatment.^[^ ^109^ ^]^ In addition, nitrogen‐doped mesoporous carbon nanocages with engineered large tunable mesoporous (≈20 nm) shells were successfully synthesized by using the colloidal SiO_2_ and the deblock copolymer PS‐*b*‐PEO micelles as dual‐template and dopamine as precursor.^[^ ^110^ ^]^ It can be seen that, the size of mesopores on shell can only be slightly tuned by changing carbon precursor or significantly tuned by distinguishing micelles of different sizes.

Recently, the highly mesoporous carbon nanocages with uniform diameters (≈320 nm), large shell thickness (≈50 nm), large pore sizes (7.5 nm), ultrahigh surface area (1582 m^2^ g^−1^), and ultrahigh pore volume (2.45 cm^3^ g^−1^) have been synthesized with the in situ generated SiO_2_ primary nanoparticles on SiO_2_ spheres as dual‐templates^[^ ^111^ ^]^ (see Figure 6B). Three small molecules are involved as precursors in the synthesis: resorcinol (R), formaldehyde (F), and tetrapropyl orthosilicate (TPOS). Relative to previous TEOS,^[^ ^80^ ^]^ TPOS undergoes slower hydrolysis and condensation behavior in ethanol and water mixed solution, which allows better control the SiO_2_ core spheres and SiO_2_ primary particles to form SiO_2_@SiO_2_/RF core–shell composite spheres (Step I). After carbonization and template removal, mesoporous carbon nanocages can be easily obtained (Step II). Based on this synthetic strategy, the pore size of carbon nanocages products could be precisely tuned by adjusting the ratios of TEOS/TPOS or ethanol/water. Typically, the mesoporous carbon nanocages with pore size of 2, 5, and 10 nm can be controllably synthesized with TEOS/TPOS of 4/1 (ethanol/water of 70/10), 100% TPOS (ethanol/water of 70/10), and 100% TPOS (ethanol/water of 60/20), respectively (see Figure 6C,D).^[^ ^112^ ^]^ More recently, by altering the reaction temperature from 6 to 45 °C or varying the time‐lag of adding RF carbon precursors from 0 to 180 min (based on the so‐called chemical reaction kinetics guided strategy), the sphere diameter, shell thickness, and pore size of the products can be successfully tailored in the range of 200–700, 10–100, and 4–19 nm, respectively.^[^ ^113^ ^]^ These results indicate this SiO_2_@SiO_2_/RF surfactant‐free method is more powerful in tuning the pore sizes than the previous soft‐templating methods,^[^ ^108^, ^109^, ^110^ ^]^ where the pores are controlled by the “soft” micelle size of surfactants (the pore size controllability is poor). However, the limitations of this double‐silica template self‐assembly strategy include that the pores are disordered and pore size distribution is relatively broad in mesoporous carbon nanocages.^[^ ^114^ ^]^ Nevertheless, such highly mesoporous carbon nanocages (i.e., hollow carbon nanospheres with tunable mesoporous walls) are currently the most popular catalyst support and nanoreactor, due to their relatively high surface area and favorable mesoporous structure.^[^ ^115^ ^]^

On the other hand, the intrinsic defects (such as vacancies and edges) in carbon materials can act as active sites for ion adsorption and intercalation, thus improving electrochemical performances.^[^ ^116^ ^]^ Particularly, the atomic‐level carbon vacancy defect distributed on basic sp^2^ carbon unit (i.e., hexagon ring of graphene), plays a significant role in foreign atom anchoring and electron density regulation.^[^ ^117^ ^]^ When a carbon atom is lost in the hexagon ring, a single vacancy carbon defect is formed on graphene, resulting in the fracture of three covalent bonds and the formation of three dangling bonds. The unsaturated dangling bonds from single vacancy carbon defects can induce strong overlap of metal orbitals and sp^2^ carbon orbitals, which effectively suppress the aggregation of foreign metal species at the defective carbon support.^[^ ^118^ ^]^ Meanwhile, the graphene‐edge linear defects (zigzag and armchair edges) from the pore structures of carbon materials can serve as highly active centers and directly participate in the chemical reaction, contributing to electrocatalytic activity.^[^ ^74^ ^]^ Generally, the vacancy and edge carbon defects can be introduced or increased by physical treatment (such as high‐energy ion beams and *γ* rays irradiation treatment) and chemical treatment (such as chemical reaction and oxidation etching for tailoring covalent bonds).^[^ ^119^, ^120^ ^]^ Apparently, the previously described activation^[^ ^103^, ^104^, ^105^, ^106^ ^]^ or template^[^ ^111^, ^112^, ^113^, ^114^ ^]^ pore‐making strategies can be used as efficient methods for the fabrication of carbon defects on the shell of hollow carbon nanocages.

### 3.4 Dispersity and Aggregation State Regulations

**Figure 7:**
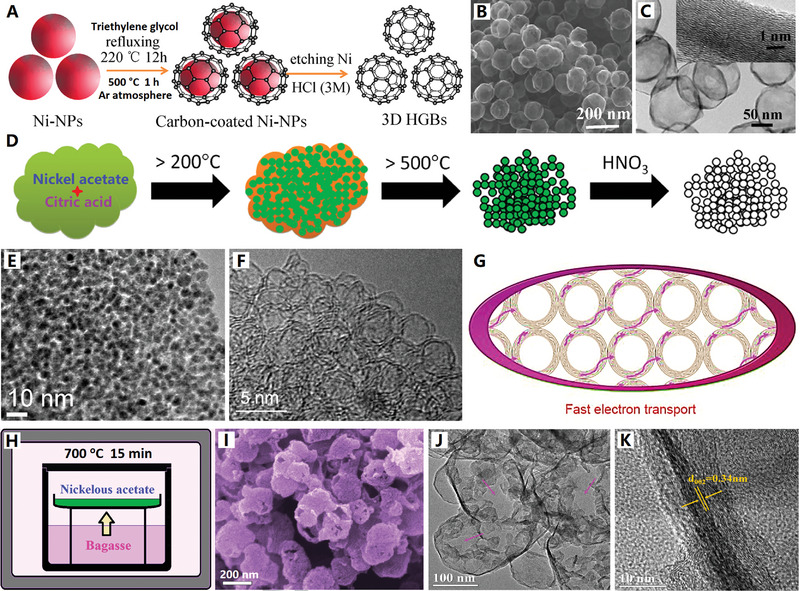
A–C) Monodispersed graphene‐like carbon nanocages. Reproduced with permission.^[^ ^122^ ^]^ Copyright 2018, Springer. D–F) Superfine‐diameter and interconnected graphene‐like carbon nanocages. Reproduced with permission.^[^ ^124^ ^]^ Copyright 2017, Elsevier. G) Fast electron transport of interconnected graphene‐like carbon nanocages. Reproduced with permission.^[^ ^125^ ^]^ Copyright 2018, Elsevier. H–K) Q‐CVD preparation of graphene‐like interconnected carbon nanocages. Reproduced with permission.^[^ ^38^ ^]^ Copyright 2017, Elsevier.

Monodispersed carbon nanocages have many advantages, such as regular morphology, uniform diameter, good fluidity, high surface reactivity, and easy functionalization, which have high research and application values in the fields of electrocatalysis and electrochemical storage.^[^ ^115^ ^]^ In general, the amorphous carbon nanocages prepared by spherical colloid templates (e.g., PS spheres and SiO_2_ spheres) have very high dispersity and good uniformity.^[^ ^36^, ^37^, ^108^, ^111^, ^112^, ^113^, ^114^ ^]^ The monodispersed graphene‐like carbon nanocages can also be prepared by using presynthesized metallic particle templates (e.g., Ni nanoparticles).^[^ ^121^, ^122^, ^123^ ^]^ For example, uniform Ni nanoparticles (Ni‐NPs) were synthesized by the reduction reaction of NiCl_2_·_6_H_2_O, N_2_H_4_·H_2_O, and NaOH, and then the graphene‐like carbon layers were coated on the surface of Ni‐NPs (with triethylene glycol as carbon source) by a successive carburization (refluxing at 220 °C) and carbon‐segregation (annealing at 500 °C) process, and the well‐shaped hollow graphene balls were obtained after etching Ni with 3 m HCl solution (see **Figure** **7**A–C for details).^[^ ^122^ ^]^ Particularly, the graphene‐like carbon nanocages possessed homogeneous spherical morphology with multilayer graphene shell (2–38 layers) and controllable diameter (100–250 nm).^[^ ^121^ ^]^ This Ni nanoparticles template‐directed synthesis approach is also proved a versatile technique to fabricate monodispersed highly crystalline graphene nanocages at elevated temperature (1200 °C).^[^ ^121^ ^]^ In addition, the carbide‐derived carbons strategy (e.g., Ni_3_C NPs‐derived hollow carbon shells) can also be applied to design monodispersed graphitic carbon nanocages.^[^ ^123^ ^]^ These monodispersed graphene‐like carbon nanocages have exhibited excellent electrochemical properties due to their good spherical shapes and high dispersion properties.

Superfine‐diameter graphene‐like carbon nanocages are a particular type of hollow carbon shell, which often interconnected into 3D mesoporous structures with significant pore volume and high specific surface area.^[^ ^124^, ^125^, ^126^ ^]^ For example, the direct pyrolysis of nickel acetate and carbonaceous molecular precursors (citric acid^[^ ^124^ ^]^ and sucrose^[^ ^125^ ^]^) mixture at moderate temperatures (500–600 °C) can produce highly homogenous, densely‐packed, interconnected graphene‐like carbon nanocages with bilayer graphene shell (see Figure 7D–F).^[^ ^124^ ^]^ The synthesis procedure consists of the following steps: 1) in situ formation of superfine Ni nanoparticles, 2) catalytic growth of graphene shells, and 3) removal of Ni nanoparticles through dissolution in acid (Figure 7D). These graphene‐like carbon nanocages with their bilayer graphene structure, showed unimodal pore size distribution of 2.5 nm and very high specific surface area of 1150 m^2^ g^−1^ (Figure 7E,F). When sucrose is used as carbon source, unique carbon nanomesh (i.e., porous nanosheets) constructed by interconnected densely packed carbon nanocages with ultrathin graphitic shells was obtained, which possessed large specific surface area (1198 m^2^ g^−1^) and high mesoporous content (91.3% volume for 2.2 nm mesopore).^[^ ^125^ ^]^ Furthermore, the interconnected graphene‐like carbon nanocages can be regarded as a 3D electrically conducting network that permits fast electron transport among the different carbon nanocages, thus achieving better electrochemical kinetics to ensure excellent rate performance (Figure 7G).^[^ ^125^ ^]^ In addition, the interconnected carbon nanocages with ultrahigh surface area and narrow nanocavities can be used as an efficient host to confine the growth of extraneous metallic‐component nanoparticles (e.g., ultrasmall Co_3_O_4_ nanoparticles anchored in superfine carbon nanocages).^[^ ^81^ ^]^ These ultrafine and tightly connected carbon nanocage networks tend to have excellent electrochemical properties due to their 3D electrically conductive networks and extremely high specific surface areas.

Recently, our research group has proposed an efficient quasi‐chemical vapor deposition (Q‐CVD) strategy for the convenient synthesis of graphene‐like interconnected carbon nanocages (see Figure 7H–K).^[^ ^38^ ^]^ Typically, bagasse as a carbon source was placed at the bottom of reaction crucible, and nickel acetate as a catalyst precursor was placed at the top of reaction crucible (see Figure 7H). When the system is heated at 750 °C, graphene‐like carbon nanocages can be formed on nickel template, by the catalysis of nickel nanoparticles formed in situ. After removing the nickel template, macroporous and ultrathin nanocages can be harvested (see Figure 7I–K). During pyrolysis, the solid bagasse first released carbonaceous gas, then carbonaceous gas deposited as solid graphene structure. This “solid–gas–solid” model is a high‐efficiency and scalable production technique, which is superior to previous synthesis methods by direct mixing and pyrolysis of nickel acetate and carbon sources. The unique macroporous structure, ultrathin nanostructure, and good graphitization structure provide favorable conditions for the electrochemical application of these graphene‐like carbon nanocages.

**Figure 8:**
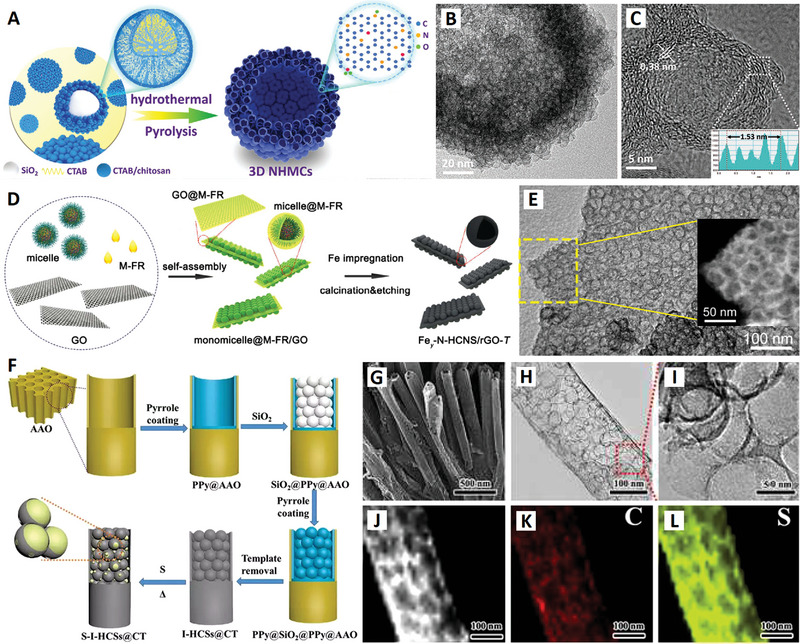
A–C) Carbon nanocages self‐assembled hollow microspheres. Reproduced with permission.^[^ ^126^ ^]^ Copyright 2020, Wiley. D,E) Carbon nanocages@rGO sandwich nanosheets. Reproduced with permission.^[^ ^127^ ^]^ Copyright 2018, ACS. F–L) Spheres‐in‐tube array structures built up of incalculable interconnected carbon nanocages. Reproduced with permission.^[^ ^128^ ^]^ Copyright 2018, RSC.

Above, we discussed the aggregation state regulations and the benefits of interconnected carbon nanocages on electrochemical performance. Compared with the monodisperse structures,^[^ ^122^ ^]^ the aggregation state network structures^[^ ^124^, ^125^ ^]^ can indeed provide more structural advantages, such as 3D electrical conductivity and structural stability. Moreover, when relying on a specific carrier or template, these interconnected carbon nanocages can be assembled into superstructures with freestanding array structures (such as hollow microspheres, sandwich nanosheets, and spheres‐in‐tube array structures built up of incalculable carbon nanocages)^[^ ^126^, ^127^, ^128^ ^]^ (see **Figure** **8** for details).

For example, Wen and co‐workers successfully prepared nitrogen‐doped carbon nanospheres with hierarchical multicavity structure by self‐sacrificing template method. The self‐assembly process of the material was accompanied by a mild high‐temperature hydrothermal etching phenomenon, so as to obtain the hierarchical multicavity structure (i.e., small carbon nanocages (≈10 nm) self‐assembled hollow microspheres)^[^ ^126^ ^]^ (see Figure 8A–C). When used as a selenium carrier, the macroporous hollow inner cavity is beneficial to achieve high quality selenium loading electrode, and the microporous/mesoporous hollow shell is similar to the micro‐electrochemical nanoreactor. Its unique multicavity structure combined with its surface amino group provides a highly conductive and high‐activity 3D carbon network. This network not only showed an effective physical–chemical double barrier effect in the process of charging and discharging of electrode, but also endowed an efficient in situ anchor‐diffusion‐transformation location for selenide, so as to obtain rapid reaction kinetics.^[^ ^126^ ^]^ This hierarchical multicavity structure made up of small carbon nanocages (≈10 nm) also demonstrated the close‐packed storage conception of ultrafine carbon nanocages array.^[^ ^78^ ^]^

Tan et al. used melamine‐formaldehyde resin‐coated triblock copolymer micelles to self‐assemble into tightly packed 2D arrangements on the surface of graphene oxide (GO) sheets. Carbonization of these structures resulted in the formation of a carbon nanocage@graphene sandwich structure, consisting of reduced graphene oxide (rGO) sandwiched between two hollow nitrogen‐doped carbon nanospheres (N‐HCNS) monolayers less than 40 nm in diameter^[^ ^127^ ^]^ (see Figure 8D,E). The sandwiched construction and mesoporous structure can increase the electrical conductivity of independent nanosheets, and increase the contact area between the material and the electrolyte, promoting the effective ion transport. Yang and co‐workers successfully encapsulated hollow carbon spheres in carbon tubes by the restricted assembly method to prepare spheres‐in‐tube superstructural nanomaterials^[^ ^128^ ^]^ (see Figure 8F–L). When it is used in Li–S battery, it shows excellent energy storage performances. The existence of interconnected carbon nanocages in large carbon tubes can optimize the conductivity of S and improve its utilization rate. The spheres‐in‐tube superstructure provides a shortened diffusion pathway for the rapid transport of protons and ions, which is very important for improving the cycling stability of sulfur at high power condition. In a word, these freestanding aggregation state superstructures of interconnected carbon nanocages provide an efficient platform for designing high‐performance electrodes for different electrochemical systems.

### 3.5 Multicavity and Polyhedral Morphology Regulations

The design and synthesis of spherical porous carbon nanocages give carbon materials some new characteristics, such as controllable size, fine structure, regular geometry, and good mobility, which are important indicators of photoelectric, catalytic, and advanced electrode materials. In particular, the construction of fine structures (such as multicavity hollow structures) plays a crucial role in the performance improvement and application expansion of new carbon materials. In electrochemical applications, the multicavity hollow structure with hierarchical porous structure, high conductivity, large pore volume, and specific surface area ensures continuous and rapid electron and ion transfer.^[^ ^129^ ^]^ The synthesis of carbon nanocages with multicavity hollow structure is still a difficult problem in synthesis methodology, because the conventional method is difficult to control the formation of internal structure and shell structure of nanocages across the micro‐ and nanoscale.

**Figure 9:**
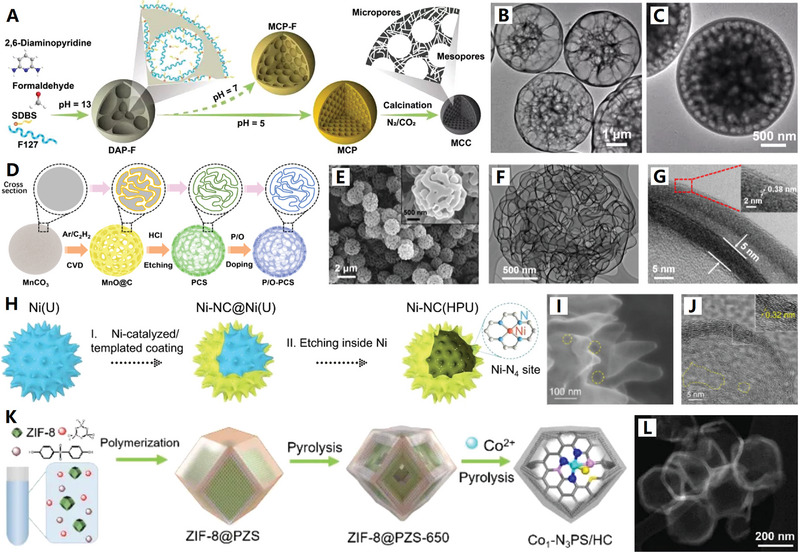
A–C) Multicavity carbon nanocages. Reproduced with permission.^[^ ^130^ ^]^ Copyright 2019, Wiley. D–G) Multicavity porous carbon nanocages. Reproduced with permission.^[^ ^132^ ^]^ Copyright 2021, Wiley. H–J) Multicavity urchin‐like carbon nanocages. Reproduced with permission.^[^ ^133^ ^]^ Copyright 2022, Wiley. K,L) Polyhedral carbon nanocages. Reproduced with permission.^[^ ^135^ ^]^ Copyright 2020, Wiley.

In 2019, Wang and co‐workers proposed a novel surfactant‐induced space‐confined polymerization strategy to synthesize a unique carbon nanocage: multicavity carbon microspheres with fine structure^[^ ^130^ ^]^ (see **Figure** **9**A–C). The precursor of this carbon nanocage is a novel multichamber polymer based on 2,6‐diaminopyridine (DAP). In the first step, DAP was grown with formaldehyde in alkaline solution to form a prepolymer with cytoskeleton‐like structure (DAP‐F). In polymerization process, polymer surfactant F127 and anionic surfactant sodium dodecyl benzene sulfonate formed a double surfactant system to control the growth of DAP‐F prepolymer. The second stage of polymerization began after the addition of acetic acid. With the decrease of solution pH, the DAP‐F prepolymer was further cross‐linked, and its polymerization in the space‐confined space of microspheres led to the space separation of large compartments to form rich small compartments. Finally, the hierarchical porous multicavity carbon nanocages can be obtained by carbonization under N_2_/CO_2_ atmosphere. In 2022, Qiao and co‐workers further reported a hierarchical growth and space‐confined polymerization strategy for the construction of tunable hollow structures in Schiff Base Polymer colloidal spheres.^[^ ^131^ ^]^ When the mass ratio of sodium dodecyl benzene sulfonate to F127 was 0, 0.05, and 0.2, single‐cavity hollow nanospheres, multicavity hollow nanospheres, and multicavity microspheres were obtained, respectively. When these polymer spheres with fine structure were carbonized into corresponding porous carbon nanocages, their internal structures were well preserved during heating. This work provides an important reference for the growth mechanism of polymer colloids and the construction of fine hollow structures in polymer and carbon nanomaterials.

In 2021, Zhao et al. used manganese carbonate as a precursor to grow a layer of carbon with a thickness of only 5 nm on the surface by chemical vapor deposition technology, and then obtained multicavity porous carbon spheres (PCS) with brain‐shape structure after removing the template by acid washing^[^ ^132^ ^]^ (see Figure 9D–G). The porous carbon sphere has a highly open 3D channel, which is conducive to ion transport and increases the contact area between electrode and electrolyte. However, the subsequent P/O codoping treatment can maintain the 3D structure of porous carbon spheres while introducing a large number of doping atoms and defective active sites, thus greatly improving the reaction kinetics based on surface adsorption in electrochemical applications. In 2022, Li et al. also developed a simple Ni catalytic and templating strategy to fabricate multicavity hollow porous sea urchin‐like N‐doped carbon nanostructures with single atom Ni sites^[^ ^133^ ^]^ (see Figure 9H–J). The unique hollow spines with high crystallinity allow for good electrical conductivity and large surface area, while achieving efficient electron/mass transfer and single atom Ni site exposure. This electrocatalyst exhibits high catalytic current density and excellent electrochemical durability. Overall, the design of multicavity carbon nanocages provides a tunable and scalable strategy for the preparation of high‐performance electrochemical materials.

On the other hand, designing polyhedral carbon nanocages is a typical morphological regulation strategy that can enhance the materials from the aspects of geometric topology. The MOFs are of great interest due to its large specific surface area and high porosity. The MOF‐derived polyhedral hollow carbon materials have great potential in improving electrochemical performances due to their unique microstructure and functionalized characteristics.^[^ ^134^ ^]^ Recently, Chen et al. developed a two‐step pyrolysis strategy derived from MOFs to design polyhedral hollow carbon nanocages with single atom Co sites^[^ ^135^ ^]^ (see Figure 9K,L). First, the ZIF‐8 composite coated with poly(cyclotriphospazene‐*co*‐4,4′‐sulfonyldiphenol) was pyrolyzed at 650 °C in Ar atmosphere to form nitrogen, phosphorus, and sulfur codoped hollow carbon polyhedral, and then Co precursor was added. The catalyst can be adsorbed in the multistage pore and then pyrolyzed at 950 °C to obtain a single atomic Co site catalyst with Co_1_‐N_3_Ps active center (Co_1_N_3_PS/hollow carbon (HC)). Because of the unique polyhedral hollow structure and single atomic site design, the catalytic efficiency and reaction kinetics are greatly improved, so that it shows excellent electrochemical performances.

## 4 Structural Modifications of Hollow Carbon Nanocages

### 4.1 Nonmetal Heteroatom Doping

Hollow nanocarbon‐based catalysts have attracted the attention of researchers in recent years due to their good electrical conductivity and adjustable surface chemical properties. The catalytic activity of hollow nanocarbon materials can be adjusted by heteroatom doping (such as boron, nitrogen, phosphorus, sulfur, and fluorine), and then the electronegativity, charge distribution, and electron transfer behavior can be properly adjusted. The codoping of nonmetal atoms with different electronegativity can introduce more active centers and improve the catalytic performances of hollow nanocarbon materials.^[^ ^66^, ^126^, ^132^ ^]^ The doping of nonmetal atoms can improve the conductivity and infiltration of carbon materials, introduce pseudocapacitance, promote the interfacial reaction between electrode and electrolyte, and improve the electrochemical energy storage performance by changing the behavior of electron donor/acceptor.^[^ ^12^, ^74^ ^]^ For example, as an electron donor, N atom doped in the carbon matrix can improve the conductivity, increase the reactive active site, change the infiltration and polarization, and change the carbon valence band and electron energy level, thus improving the electrochemical performance for capacitance energy storage. In addition, the uniform N, P,S codoped carbon skeleton facilitates electron and ion transport, while the hollow structure also provides a wide space to buffer volume expansion for electrochemical energy storage.^[^ ^136^ ^]^

**Figure 10:**
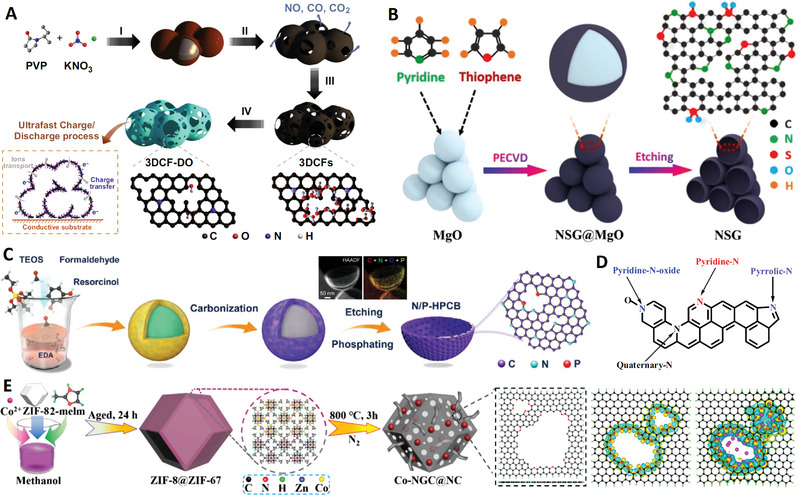
A) N‐doped interconnected carbon nanocages with less oxygen groups. Reproduced with permission.^[^ ^137^ ^]^ Copyright 2021, Springer. B) N/S dual‐doped graphitic carbon nanocages. Reproduced with permission.^[^ ^138^ ^]^ Copyright 2021, Wiley. C) N/P dual‐doped graphitic carbon nanocages. Reproduced with permission.^[^ ^139^ ^]^ Copyright 2021, Wiley. D) Schematic diagram of N doping structure of graphene. Reproduced with permission.^[^ ^140^ ^]^ Copyright 2020, Wiley. E) Cobalt (Co)‐modified N‐doped core–shell carbon nanocages. Reproduced with permission.^[^ ^142^ ^]^ Copyright 2020, Wiley.

In 2021, Qiu and Zhao used chemical foaming and in situ activation methods to fabricate 3D N‐doped carbon nanomaterials constructed from interconnected carbon nanocages, starting from the polyvinyl pyrrolidone (PVP) and potassium nitrate (KNO_3_) precursors^[^ ^137^ ^]^ (see **Figure** **10**A). The 3D carbon nanocages material has high surface area, hierarchical pore structure, good electrical conductivity, and suitable nitrogen content. The interconnected and hierarchical carbon nanocages are conducive to the storage and improve the charging and discharging rate. High‐specific surface area is conducive to high electric double layer capacitance, and the N‐doped structure gives considerable pseudocapacitance, which provides a possibility for the construction of high‐energy and high‐power supercapacitors. In addition, the removal of oxygen‐containing functional groups on the surface of the carbon nanocages has three benefits: 1) electrolyte ions are easy to enter the pore interior, which is helpful for ion transport in the process of charging and discharging, (2) improves the conductivity of the material and promotes the rapid transfer of electrons, and 3) avoid all kinds of side reactions and gas generation, make material as electrode more stable in high potential window.^[^ ^137^ ^]^

Doping heteroatoms (N, S, P, B, etc.) to carbon skeletons has been shown to be a feasible way to significantly improve the electrochemical performance of metal ion batteries (MIBs). For example, N doping can generate enough active sites to increase the interlayer distance of pure carbon, which is beneficial to improve the rate performance and buffer volume expansion. In the search for further improvement of carbon negatives in MIBs, codoping heteroatoms has proved to be an effective solution to ultimately improve conductivity, enhance metal ion capture, and facilitate reaction kinetics. In 2020, Liu et al. used low‐temperature plasma‐enhanced chemical vapor deposition (PECVD) strategy to precisely synthesize nitrogen/sulfur dual‐doped graphitic carbon (NSG) hollow nanocages in one step^[^ ^138^ ^]^ (see Figure 10B). The NSG has uniform N/S dual‐doping structure, abundant potassiophilic surface moieties, efficient electron/ion transport paths, and enhanced electrode–electrolyte interactions, which are important for high‐rate performance and extended cycle life. The hollow structure of NSG makes it have structural stability, so as to adapt to the volume change of graphite layer in the charging and discharging. The N/S dual doping leads to the increase of layer spacing and defects, which is conducive to the adsorption, diffusion, and embedding of K ions.

In addition, hard carbon, or nongraphite carbon, is considered to be the most attractive candidate for battery anode materials due to its high degree of disorder, less crystalline structure, and adjustable interlayer spacing. It is necessary to design a hard carbon material with the advantages of heteroatom doping, stable hollow porous structure, and large layer spacing to significantly improve the potassium storage performance. In 2021, Wang and co‐workers further developed a potential doped carbon nanocage material for high‐performance potassium ion batteries: a hollow bowl‐shaped hard carbon structure with nitrogen/phosphorus double doping and high porosity (N/P‐HPCB)^[^ ^139^ ^]^ (see Figure 10C). The N/P‐HPCB has high content of N/P double doping, large specific surface area, extended layer spacing, and strong structural stability, which make it exhibit extraordinary K ions storage capacity and electrochemical performance. The excellent performance of N/P‐HPCB could be attributed to the comprehensive advantages of high content of N/P double doping, expanded layer spacing, more defects and active sites, and ultrastable porous bowl‐shaped hollow structure.

In three nitrogen dopant types, pyridine N, pyrrole N, and graphitized N^[^ ^140^ ^]^ (see Figure 10D), the highly coordinated N atom of the graphitized N replaces the C atom in the graphene layer, which is beneficial to enhance electron transfer and improve electrical conductivity. The pyridine N and pyrrole N at the carbon edge are expected to contribute positively to the electrochemical performances by improving the adsorption capacity of active ions through coordination effects.^[^ ^141^ ^]^ However, the controllable design of pyridine N and pyrrole N on carbon nanocages remains a great challenge. In 2020, Zhang and co‐workers obtained cobalt‐modified N‐doped (abundant pyridine N and pyrrole N) core–shell carbon nanocages (Co‐NGC@NC) for anode materials of high‐performance lithium‐ion batteries by pyrolysis with a double‐layer MOF structure (ZIF‐8@ZIF‐67)^[^ ^142^ ^]^ (see Figure 10E). The Co‐NGC@NC has a robust polyhedral carbon nanocage structure, and its surface carbon nanotubes and cobalt nanoparticles in the nanocage can effectively improve the electronic conductivity of the material. In addition, this hollow carbon materials have unique dominant N‐doped structure (pyridine N and pyrrole N) at the hole edge, so the sites and spaces of lithium embedded are rich, and it is easy to obtain a large pseudocapacitance in the process of charging and discharging.

### 4.2 Metal Single/Dual Atom Doping

An ideal catalyst for energy storage and conversion needs to have high catalytic activity, good stability, low cost, and high metal utilization ratio. In recent years, single atom catalysts (SACs) have attracted much attention due to their extremely high atomic utilization ratio and catalytic activity. For the metals (M)‐based SACs, the metal species are typically immobilized on carbon nanomaterials by the coordination with doped N atoms (dispersing metal species via the so‐called M–N_4_ moieties).^[^ ^143^ ^]^ Moreover, most SACs currently have only a single catalytic function, which cannot meet the multifunctional requirements of catalysts in energy devices such rechargeable metal–air batteries and all‐water decomposition devices. Therefore, it is urgent to develop high performance bimetallic SACs (or dual atom catalysts (DACs)) by metal atom dual doping on carbon supports.^[^ ^144^ ^]^ The N‐doped hollow carbon nanocages are promising carbon supports for the construction of high‐performance SACs or DACs, due to their rich nitrogen content and potential carbon defects.

**Figure 11:**
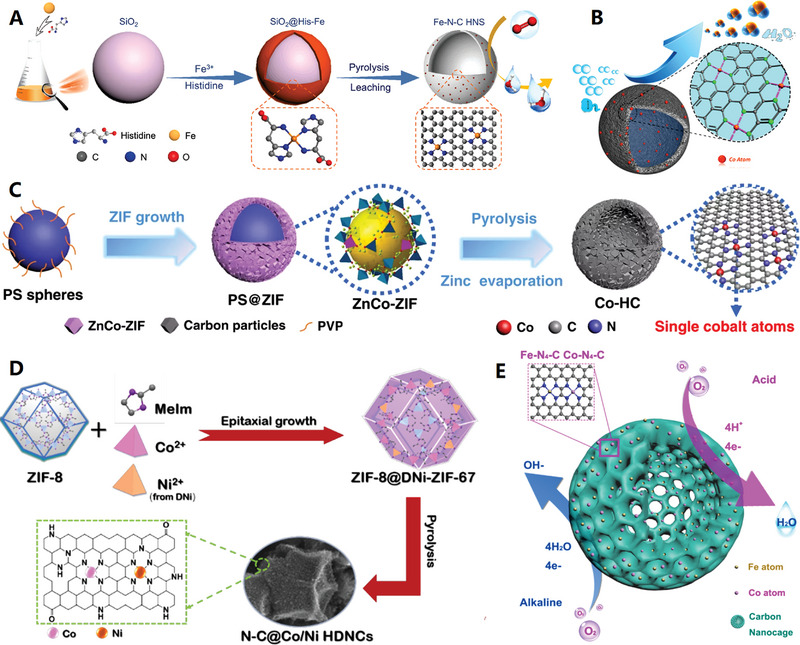
A) Fe single atoms doping on N‐doped hollow carbon spheres. Reproduced with permission.^[^ ^145^ ^]^ Copyright 2019, Wiley. B) Co single atoms doping on N‐doped hollow carbon spheres. Reproduced with permission.^[^ ^146^ ^]^ Copyright 2017, ACS. C) Co single atoms doping on N‐doped porous hollow carbon spheres. Reproduced with permission.^[^ ^147^ ^]^ Copyright 2020, Nature. D) Co–Ni dual atoms doping on hollow N‐doped carbon polyhedrons. Reproduced with permission.^[^ ^148^ ^]^ Copyright 2021, Elsevier. E) Fe–Co dual atoms doping on N‐doped porous hollow carbon nanocages. Reproduced with permission.^[^ ^149^ ^]^ Copyright 2021, Wiley.

In 2019, Tang and co‐workers, using the inexpensive and sustainable biomaterial Histidine (His) as an N and C source, have carefully designed a SiO_2_ as a template for the synthesis of immobilized and dispersed Fe single atoms on hollow N‐doped carbon spheres (Fe–N–C HNSs)^[^ ^145^ ^]^ (see **Figure** **11**A). The Fe^3+^ ions can be easily adsorbed on the surface of modified SiO_2_ nanospheres by electrostatic attraction. Subsequently, His molecules bind closely with Fe^3+^ ions through coordination interaction to form SiO_2_@Fe‐His nanospheres. After pyrolysis in an inert atmosphere, the Fe^3+^‐His layer is carbonized to N‐doped carbon, and the Fe^3+^ ions are converted into metal elements at the same time. Finally, SiO_2_ template and large metal particles are removed by leaching in HF solution, resulting in the formation of single Fe atoms fixed on N‐doped carbon hollow nanospheres. With atomically dispersed Fe–N_4_ and a unique hollow structure, the Fe‐N‐C HNSs exhibit excellent oxygen reduction reaction (ORR) performance in alkaline media, with high activity, long‐term stability, and excellent tolerance to methanol, outperforming commercial Pt/C catalysts and most reported non‐noble metal catalysts.^[^ ^145^ ^]^

The well‐dispersed single‐atom metal species doped on hollow carbon nanocages provide an opportunity to achieve maximum atomic efficiency and expose the most active sites. Compared with metallic nanomaterials, well‐dispersed atomic catalysts are more stable, especially in acidic media. In 2017, a SiO_2_ template‐induced pyrolysis strategy was used to prepare a well‐dispersed catalyst of single atom Co immobilized on N‐doped hollow carbon spheres (with abundant Co–N_4_ moieties) by Li and co‐workers^[^ ^146^ ^]^ (see Figure 11B). The single Co–N_4_ moieties and hollow substrate give the catalyst excellent ORR performance in acidic medium (the half‐wave potential is close to Pt/C). Experiments and density functional theory confirm that the enhanced hydrogenation of OH* by dispersed Co is the source of high ORR activity. In 2020, Wang and collaborators also developed a mild PS template strategy to prepare Co single atoms/N‐doped hollow carbon (CoSA‐HC) catalysts for high‐performance lithium–selenium batteries^[^ ^147^ ^]^ (see Figure 11C). The PVP‐modified PS spheres were mixed with Co(NO_3_)_2_, Zn(NO_3_)_2_, and 2‐methylimidazole salts in methanol solution, and PVP@Zn‐Co ZIF was formed on the surface of PS spheres. After pyrolysis, the PS template and Zn were evaporated in situ to form porous hollow carbon and well‐dispersed Co–N_4_ moieties.

In a bimetallic atom catalyst, when the distance between two different single atoms is close enough, there will be strong interaction between them, and then the double active center with synergistic effect will be formed, and the catalytic performance will be significantly improved.^[^ ^144^ ^]^ In 2021, Wang et al., using CoNi containing bimetallic MOFs as precursors, prepared atomic‐scale Co–Ni bimetallic active site modified N‐doped carbon hollow nanocage composites (N‐C@Co/Ni HDNCs) by epitaxial growth and high‐temperature pyrolysis strategy, and used them as the negative electrode of lithium‐ion batteries^[^ ^148^ ^]^ (see Figure 11D). The lithium storage properties of the materials and the mechanism of atomic Co–Ni bimetallic active sites in the process of lithium storage were revealed by experimental and theoretical calculations. In terms of structure, hollow and porous structures are cleverly designed to alleviate volume expansion and effectively shorten the diffusion distance of lithium ions. The atomic level of Co–Ni double active site, with super Li affinity and synergistic catalytic effect, makes the prepared anode materials show excellent lithium storage capacity.

At present, the controllable preparation of adjacent single‐atom double‐active center structures is still a very challenging topic. Meanwhile, the characterization of adjacent single‐atom double‐active centers from different elements is also a major problem. Recently, Li et al. used selective polymerization to prepare N‐doped hollow carbon nanocages with double active centers of adjacent Fe–N_4_–C and Co–N_4_–C structures as efficient catalysts for ORR^[^ ^149^ ^]^ (see Figure 11E). It shows excellent ORR performance in both acidic and alkaline media. The structures of adjacent Fe–N_4_–C and Co–N_4_–C double active centers in this Fe–Co DACs were determined by high‐resolution spherical electron microscopy, synchrotron radiation spectroscopy, and microregion electron energy loss spectroscopy. The experimental tests and theory calculation show that this Fe–Co DACs has better catalytic activity than Fe or Co SACs in ORR, which is due to the synergistic effect of Fe–N_4_–C and Co–N_4_–C double active centers to reduce the energy barrier of ORR reaction.

### 4.3 Composite Interface Designing

The interface, known as the boundary of two different components, has been shown to exhibit unique properties compared to individual components. The interface formed between two types of active materials can form more active centers than a single component because of strong chemical bonds, electronic interactions or synergies. According to the type of composite material, the interfaces can be divided into different categories, among which the interfaces between metals (or metal compounds) and carbon materials are widely used in the multiple electrochemical fields.^[^ ^150^ ^]^ The interface engineering of hollow carbon nanocages can significantly improve the electrochemical activity and stability of the hollow materials, because of the strong interface interaction can create robust hollow structure, high‐transfer‐rate channel, and highly exposed active centers.

**Figure 12:**
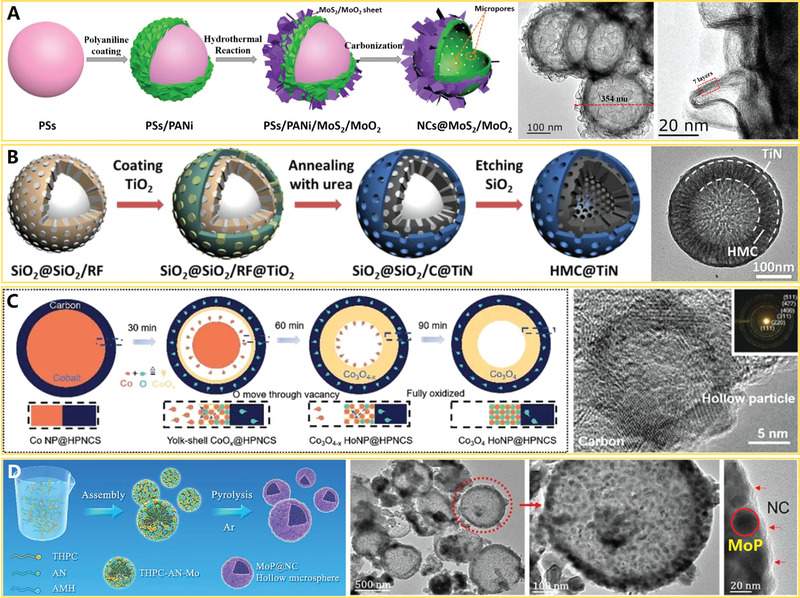
A) Microporous carbon nanocages@MoS_2_/MoO_2_ interface material. Reproduced with permission.^[^ ^151^ ^]^ Copyright 2018, ACS. B) Mesoporous carbon nanocages@TiN interface material. Reproduced with permission.^[^ ^152^ ^]^ Copyright 2017, Wiley. C) Co_3_O_4_ hollow spheres@carbon nanocages interface material. Reproduced with permission.^[^ ^153^ ^]^ Copyright 2019, Wiley. D) MoP@N‐doped carbon (N) composite nanocages interface material. Reproduced with permission.^[^ ^154^ ^]^ Copyright 2021, Elsevier.

The design of hollow carbon nanocages‐based interfacial composites includes two approaches: 1) to construct hollow carbon nanocages coated with metal or metal compounds and 2) to construct metal or metal compounds encapsulated by hollow carbon nanocages. In 2018, Zhang and co‐workers proposed a very simple method to synthesize double‐layer N‐doped microporous hollow carbon nanocages@MoS_2_/MoO_2_ nanospheres (denoted as NCs@MoS_2_/MoO_2_) via a Mo‐mediated in situ growth on polystyrene (PS) spheres surrounded by a PANI shell and subsequent carbonization process^[^ ^151^ ^]^ (see **Figure** **12**A). In the carbonization step, the template of PS was decomposed to form a shell structure with abundant surrounding micropores, and PANI was converted into N‐doped carbon nanocages. Therefore, the as‐designed NCs@MoS_2_/MoO_2_ interfacial composites have numerous active sites for electrochemical energy storage, short ion transport path, and high‐rate performance. In 2017, Lou and co‐workers designed and synthesized a hollow mesoporous carbon nanocages@titanium nitride (HMC@TiN) interface nanostructure as SeS_2_ carrier for Li–SeS_2_ battery anode material^[^ ^152^ ^]^ (see Figure 12B). Benefiting from the physical and the chemical trapping of hollow mesoporous carbon nanocages and TiN components, the utilization rate of positive active substances in HMC@TiN/SeS_2_ is much higher and the cycling stability is much better than the single HMC/SeS_2_. In general, hollow carbon nanocages can be used as a good conductive carrier to load active components on their surface, which can be used to design high‐performance electrode materials.^[^ ^151^ ^]^ Moreover, the compact interfacial effect of carbon nanocages@metal compounds can further promote the electrochemical energy storage characteristics of the hollow interfacial materials.^[^ ^152^ ^]^

In 2019, a research group led by Prof. Guo at Peking University proposed a simple method. In the absence of damaging multistage porous N‐doped carbon nanostructures (HPNCS), oxygen vacancies are introduced into Co_3_O_4_ hollow particles to form Co_3_O_4_‐X HoNP@HPNCS, and the number of oxygen vacancies is regulated and controlled by Kirkendall effect^[^ ^153^ ^]^ (see Figure 12C). The oxygen vacancy‐rich Co_3_O_4_‐X HoNP@ HPNCS (metal@carbon) mixed nanocage has a very good catalytic effect on ORR and oxygen evolution reaction (OER) in alkaline media, and it has excellent battery performance when applied to the air electrode of portable zinc–air battery. In 2021, Liu et al. collaborated to successfully prepare molybdenum phosphide nanoparticles and nitrogen‐doped carbon (MoP@NC) complex hollow nanocages by designing organic and inorganic composite precursors, combining nontemplate and annealing processes^[^ ^154^ ^]^ (see Figure 12D). The results show that the synergism between nitrogen‐doped carbon hollow nanocages and molybdenum phosphide nanoparticles (metal@carbon) can improve the dissociation rate of adsorbed water and accelerate the hydrogen production in alkaline electrocatalysis. Among them, the d‐band center of Mo decreases at the pyridine N‐MoP interaction site, thereby weakening the Mo‐H_ads_ bond and improving hydrogen evolution reaction (HER) performance. Particularly, these metal@carbon nanocages can confer higher structural integrity and superior electrochemical stability for the metal components because of the protective effect of carbon coating structures.

### 4.4 Ship‐in‐Bottle Structure Designing

**Figure 13:**
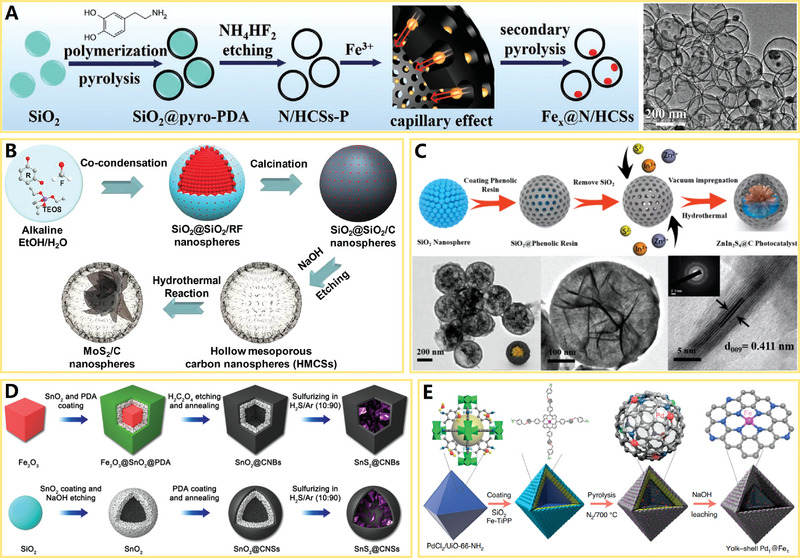
A) Fe_3_O_4_ nanoparticles in N‐doped hollow mesoporous carbon nanocages. Reproduced with permission.^[^ ^157^ ^]^ Copyright 2020, Wiley. B) MoS_2_ nanoflowers in hollow mesoporous carbon nanocages. Reproduced with permission.^[^ ^158^ ^]^ Copyright 2017, ACS. C) ZnIn_2_S_4_ nanoflowers in microporous carbon nanocages. Reproduced with permission.^[^ ^160^ ^]^ Copyright 2021, ACS. D) SnS_2_ nanosheets in hollow carbon nanocages. Reproduced with permission.^[^ ^161^ ^]^ Copyright 2018, Elsevier. E) Pd_1_@Fe_1_ yolk–shell structure of polyhedral carbon nanocages. Reproduced with permission.^[^ ^162^ ^]^ Copyright 2021, Nature.

The “ship‐in‐bottle” method was first proposed by Sulikowski in 1996, where phosphotungstic acid was successfully encapsulated in the Y‐molecular sieve’ supercages.^[^ ^155^ ^]^ The method is to spread the raw material small molecules into the supercages of the molecular sieve for the synthesis of heteropoly compounds. Due to the small size of the molecular sieve, the synthesized heteropoly compounds will not escape through the mouth of the molecular sieve, and is encapsulated in the supercage. This preparation process is like the process of building a “ship” in a “bottle,” so it is vividly called “ship‐in‐bottle” method. In 2012, Xiao et al. successfully prepared various noble metal nanostructures (gold nanorods and gold nanospheres) in hollow mesoporous silica microspheres (i.e., mesoporous silica nanocages) by using the “ship‐in‐bottle” method.^[^ ^156^ ^]^ These hollow nanocage‐encapsulated metal nanostructures have broad application prospects as recyclable and efficient catalysts for various liquid‐phase catalytic reactions. In recent years, hollow carbon nanocages have also been widely used in the design of complex nanostructures via the “ship‐in‐bottle” method. These nanostructures have shown satisfactory performances in various electrochemical systems, due to their unique “ship‐in‐bottle” constructions and extra functionalities (see **Figure** **13** for details).^[^ ^157^, ^158^, ^159^, ^160^, ^161^ ^]^

In 2020, Xia and co‐workers reported space‐confined yolk–shell construction (i.e., “ship‐in‐bottle” construction) of Fe_3_O_4_ nanoparticles (with Fe–N*_x_* active sites) inside the N‐doped hollow mesoporous carbon spheres (Fe*_x_* @N/HCSs)^[^ ^157^ ^]^ (see Figure 13A). First, the uniform N‐doped hollow mesoporous carbon spheres with ultrathin (≈8 nm) carbon layer were prepared by calcining SiO_2_@polydopamine and etching by NH_4_HF_2_. The N/HCSs‐P serves as nanoreactors, and the Fe^3+^ permeates into the inner voids of porous N/HCSs‐P via the melting–diffusion strategy with capillary force. In the secondary pyrolysis process, N/HCSs‐P can control the confined growth of Fe_3_O_4_ nanoparticles inside the hollow carbon sphere. The constructed yolk–shell structured Fe_20_@N/HCSs ecosystem with Fe–N*_x_* active sites exhibits excellent ORR and OER activity and stability, which even surpass commercial grade Pt/C, RuO_2_, IrO_2_, and many reported catalysts. This work provides a new avenue for the design and optimization of high‐performance yolk–shell materials with nanoscale confinement structures.

As early as 2017, Chen's team have developed an innovative method to generate MoS_2_ 3D nanosheets (nanoflowers) inside the hollow mesoporous carbon spheres (HMCSs)^[^ ^158^ ^]^ (see Figure 13B). The hollow carbon nanocages (i.e., HMCS) synthesized by SiO_2_@SiO_2_/RF template method can be used as nanoreactors to effectively control and limit the in situ growth of MoS_2_ nanoflowers in hydrothermal process via the “ship‐in‐bottle” design philosophy. When used as a cathode material for Li‐ion batteries, the yolk–shell structure MoS_2_@C shows higher reversible capacity and rate properties compared to pure MoS_2_ and MoS_2_ attached to carbon spheres (C@MoS_2_). The energy density of Li‐ion batteries can be enhanced by increasing the mass fraction of active substances, and the gap between the yolk (MoS_2_ nanoflowers) and the shell (hollow carbon nanocages) can be used as the volume expansion buffer of active substances when lithium is removed.^[^ ^158^ ^]^ The HMCSs can also regulate and limit the growth of Ni_3_Si_2_O_5_(OH)_4_ nanosheets, which obviously enhance the electrosorption capacity in capacitive deionization.^[^ ^159^ ^]^ In 2021, Yang and co‐workers also designed a nanoconfinement‐induced ZnIn_2_S_4_ nanoflowers@microporous carbon nanocages by the “ship‐in‐bottle” method, which can increase the water content (stabilized the water molecules inside the nanocage), and change the local electron distribution of water molecules on the surface of ZnIn_2_S_4_^[^ ^160^ ^]^ (see Figure 13C). The chemisorption capacity of the ZnIn_2_S_4_ photocatalyst to the inner water is sensitive to the microporous nanocage structure, thus further improving the photocatalytic performances.

In addition to the postinjection approach mentioned above, the synchronous synthesis approach can also be used to construct the delicate “ship‐in‐bottle” composite nanostructures. Tin disulfide (SnS_2_) is a promising anode material for sodium ion batteries because of its high specific capacity. However, the low conductivity and large volume variation of sodium ions limit its practical application. In 2018, Liu et al. used one‐step template and subsequent sulfuration strategy to reasonably design and synthesize SnS_2_ nanosheets in two hollow carbon nanocages (carbon nanoboxes (CNBs) and carbon nanospheres (CNSs))^[^ ^161^ ^]^ (see Figure 13D). Due to their unique “ship‐in‐bottle” structural advantages, the SnS_2_@CNBs and SnS_2_@CNSs show improved sodium storage performance in terms of high specific capacity, good cycling stability, and superior rate performance. In 2021, Wu and co‐workers also reported the preparation of polyhedral nanocage single‐atom yolk–shell structures (Pd_1_@Fe_1_) by a synchronous template and subsequent pyrolysis strategy^[^ ^162^ ^]^ (see Figure 13E). The composite structure contains Fe_1_ site dispersed in the shell of N‐doped carbon and Pd_1_ site located in the yolk, respectively. The catalyst generates O_2_ and H_2_ in situ as oxidant and reducing agent in electrocatalytic water decomposition, catalyzes hydrogenation of nitroaromatics, epoxidation of olefin, and cascade catalytic synthesis of amine alcohols. The findings in this paper provide experience and examples for the integration of multiple single metals in the same carbon nanocage catalytic system for complex, continuous, and difficult reactions.

### 4.5 Spatial‐Separation Dual‐Function Designing

In the field of thermal catalysis, bifunctional or even multifunctional catalysts with spatially separated sites are often used to achieve optimal catalytic performance in complex reactions involving multiple successive steps, such as hydrocarbon upgrading, biomass conversion, and direct syngas conversion.^[^ ^163^ ^]^ In the field of photocatalysis, it is a feasible way to realize the effective synergistic effect of redox cocatalysts by considering the mechanism of cocatalyst and realizing the independent channel of electron and hole through the space separation of double cocatalysts.^[^ ^164^ ^]^ In the field of electrocatalysis, some bifunctional electrocatalysis systems (such as metal–air cells and overall water splitting) often require two‐site spatial isolation to achieve independent and efficient electrocatalytic reactions.^[^ ^64^, ^165^ ^]^ In 2012, Domen group proposed for the first time that the structural properties of hollow structures can be used to load the oxidation and reduction cocatalysts on the two sides of the shell, so as to achieve the spatial separation of the oxidation–reduction centers.^[^ ^166^ ^]^

**Figure 14:**
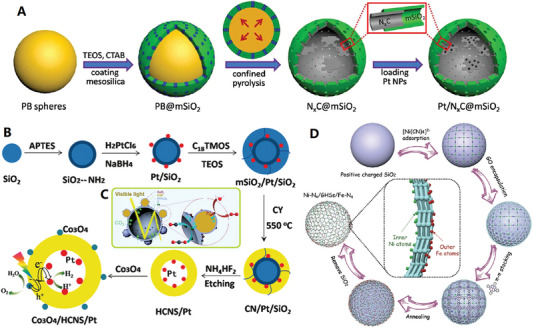
A) N‐doped carbon@silica Janus amphiphilic hollow nanocages. Reproduced with permission.^[^ ^167^ ^]^ Copyright 2022, ACS. B) Janus Pt and Co_3_O_4_ on hollow carbon nitride nanocages. Reproduced with permission.^[^ ^168^ ^]^ Copyright 2016, Wiley. C) Improving of light absorption by multilight reflection in carbon nanocages. Reproduced with permission.^[^ ^169^ ^]^ Copyright 2022, ACS. D) Janus Ni–N_4_ and Fe–N_4_ on hollow graphene nanocages. Reproduced with permission.^[^ ^170^ ^]^ Copyright 2020, Wiley.

In recent years, more hollow carbon nanocages have been used in spatial‐separation dual‐function designing for different energy catalysis fields. In 2022, Dai et al. reported a novel multifunctional amphiphilic nanoreactor consisting of N‐doped carbon@silica Janus hollow nanocages and platinum (Pt) nanoparticles, using core–shell polybenzoxazine (PB)@mesosilica (m‐SiO_2_) spheres as pyrolysis precursors^[^ ^167^ ^]^ (see **Figure** **14**A). Using air as oxidant, this Janus nanoreactor has significant activity and selectivity for alkali‐free aerobic oxidation of alcohol in water. Moreover, due to the anchoring effect of nitrogen and the extremely stable amphiphilicity, the nanoreactor shows good catalytic stability. These facts prove that the amphiphilic character (i.e., hydrophobicity and hydrophilicity) of hollow nanoreactor catalysts has a significant effect on their catalytic properties by regulating the adsorption, transfer, and desorption of reactants/products. The spatial‐separation amphiphilic function designing based on hollow carbon nanocages is a promising approach for bifunctional catalysis.

Bifunctional reactions (such as redox reactions) are usually caused by the synergistic effect of different active sites, and the bifunctional synergistic effect can be achieved by constructing compartmentalized nanoreactors. Janus catalyst based on hollow nanocages has typical spatial compartmentalization effect. For example, the redox function of HCNS can be greatly improved by loading Pt and Co_3_O_4_ particles on the inner and outer surfaces, respectively^[^ ^168^ ^]^ (see Figure 14B). The HCNS structure has separate reduction centers and oxidation centers, and the HCNS structure has strong photocatalytic redox activity. Moreover, this improved performance is due to the unidirectional migration structure of electrons and holes on both surfaces, which can suppress undesired reverse reactions and further contribute to the suppression of charge recombination. In addition, the improving of light absorption by multilight reflection in hollow carbon spheres, combined with high electrical conductivity of carbon to promote the separation of the electron hole, adsorption, and activation of molecules through the porous structure of the carbon shell, and space separation of reduction and oxidation catalyst promoters, can significantly improve the catalytic activity and product selectivity for the CO_2_ photoreduction^[^ ^169^ ^]^ (see Figure 14C).

In 2020, Qiu, Fu, and Ma (co‐corresponding author) jointly developed a stepwise self‐assembly method for immobilizing nickel and iron single atoms on the inner and outer sides of graphene hollow nanospheres (GHSs), realizing the functionalization of different single atoms on the two sides of hollow graphene^[^ ^170^ ^]^ (see Figure 14D). It was found that the Ni–N_4_/GHSs/Fe–N_4_ dual‐sided material was formed by the coordination of nickel and iron atoms with four nitrogen atoms (N_4_) by forming the plane conformation of Ni–N_4_ or Fe–N_4_. In this material, the outer Fe–N_4_ cluster has high activity ORR effect, while the inner Ni–N_4_ has OER performance, so that the material exhibits excellent bifunctional electrocatalytic properties. Theoretical calculation also proved the decisive role of dual‐sided structure and single‐atom activity on electrochemical performance. Particularly, in the zinc–air battery, the air negative electrode composed of Ni–N_4_/GHSs/Fe–N_4_ is superior to the Pt/C+RuO_2_ electrode, which gives the battery excellent energy efficiency and cycle stability.^[^ ^170^ ^]^ In 2021, Kim et al. also proposed a bifunctional polyhedral carbon nanocage electrocatalyst composed of Co_9_S_8_ (OER activity) and MoS_2_ (HER activity), which has a hierarchical structure, huge electrochemical active center, and strong charge transfer ability for electrocatalytic overall water splitting.^[^ ^165^ ^]^

## 5 Electrochemical Energy Storages and Conversions of Hollow Carbon Nanocages

Hollow characteristics of carbon nanocages are highly favored in the electrochemical field in the structural design and geometric modification of electrode materials. The cavity efficiently facilitates material/charge transport and reduces mechanical stress, thus ensuring high structural stability and magnification properties of the electrode material, especially during long cycles.^[^ ^8^ ^]^ The hollow carbon nanocages and their composite electrode materials are widely used in electrochemical energy storage (supercapacitors and rechargeable batteries (metal‐ion batteries, lithium–sulfur batteries, etc.)). The supported‐type catalyst (from highly dispersed nanostructures to single atomic structures) or metal‐free catalyst based on hollow carbon nanocages can be used for efficient electrocatalytic conversion (electrocatalytic water splitting, oxygen reduction, carbon dioxide reduction, etc.)

### 5.1 Application Case Analysis of Hollow Carbon Nanocage for Supercapacitors

As a new type of energy storage element, supercapacitor has the advantages of high power density, short charging and discharging time, good cycle stability, and so on. It fills the gap between traditional capacitor and battery and has broad application prospects.^[^ ^11^ ^]^ The unique structures and intrinsic properties of hollow carbon nanocage materials (and their composite materials) make them promising as ideal electrode materials for supercapacitors (including electric double layer capacitors (EDLCs), Faraday pseudocapacitors (PCs), and metal‐ion hybrid capacitors (HCs)).

#### 5.1.1 Electric Double Layer Capacitors

The suitable electrode material has a great influence on the capacitance of the EDLCs. HCNCs is an important electrode material. It is generally believed that the regular spherical morphology is conducive to the full contact between the electrode and the electrolyte, the cavity can store the electrolyte, and the porous shell can promote the rapid charge transfer.^[^ ^38^ ^]^ The hierarchical pore structure is very suitable for supercapacitors, in which the micropore is the main storage site of electrolyte ions, the mesoporous is the fast channel of ion transport, and the large pore is the reservoir of electrolyte.^[^ ^7^ ^]^ On the other hand, based on the wettability and capacitance characteristics, using heteroatoms to modify the surface of the electrode is another effective way to improve its performance. Therefore, it is of great significance to explore the efficient preparation of doping HCNCs with improved electrochemical performances.^[^ ^171^ ^]^ At the same time, the nanomorphology also has a great influence on the electrochemical performances of HCNCs. In the following, we will introduce the application progress of EDLCs based on the morphology of carbon nanocages, which mainly includes three aspects: amorphous carbon nanocages, graphene‐like carbon nanocages, and hollow porous (micro/mesoporous) carbon nanospheres.

##### Amorphous Carbon Nanocages for EDLCs

**Figure 15:**
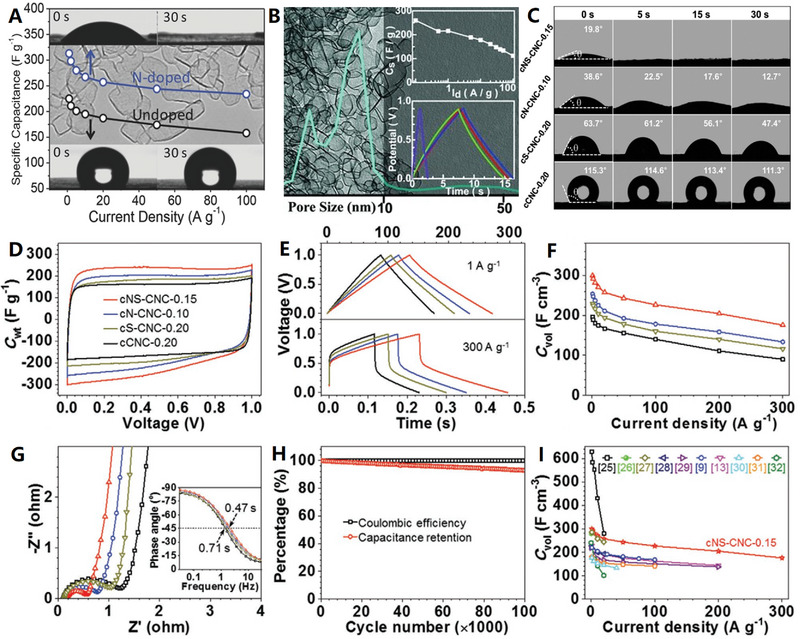
Amorphous carbon nanocages for high‐performance EDLCs. A) MgO‐templated polyhedral carbon nanocages. Reproduced with permission.^[^ ^23^ ^]^ Copyright 2012, Wiley. B) N‐doped 3D hierarchical carbon nanocages. Reproduced with permission.^[^ ^8^ ^]^ Copyright 2015, Wiley. C–I) Collapsed N,S double‐doped carbon nanocages (cNS‐CNC‐0.15). Reproduced with permission.^[^ ^88^ ^]^ Copyright 2020, Wiley.

In 2012, Prof. Hu's team reported that amorphous carbon nanocages were prepared with benzene precursor by MgO in situ template method, where the large specific surface area, good medium porosity, and polyhedral structure of these amorphous nanocages make them have excellent capacitance performances^[^ ^23^ ^]^ (see **Figure** **15**A). Recently, Hu's team further reported an effective method (capillary force compression route) to improve the volumetric properties of carbon materials, namely, to fabricate folded carbon nanocages with high density and optimized porous structure: an ideal model system is developed to show the correlation between volume properties and pore size distribution.^[^ ^26^ ^]^ The results show that it is an effective strategy to improve the volumetric energy density by reducing the gap between large and medium pores while maintaining high power density. In 2015, Hu's team prepared unique N‐doped 3D hierarchical carbon nanocages with large specific surface area, multiscale porous structure, good electrical conductivity, and excellent wettability^[^ ^8^ ^]^ (see Figure 15B). The synergistic effect of these properties ensures sufficient space for charge storage and fast transport of ions and electrons. The N doping and hierarchical structure can greatly improve the hydrophilicity of materials and effectively increase the surface accessibility to ions and promote the ion diffusion within pores, thereby reducing the charge‐transfer resistance and enhancing the performances of device.

In 2020, Hu's team constructed collapsed N,S double‐doped amorphous carbon nanocages via the capillary compression method (see Figure 15C–I for details).^[^ ^88^ ^]^ This compression eliminates excess mesopore and macropore, thus greatly increasing the density with a high surface area. The N,S double doping induces strong polarity on the carbon surface, which greatly improves wettability and charge transfer. The synergistic effect of high density, large ion receiving surface area, and fast charge transfer enables high‐capacity performance at high rates. As a result, the EDLCs based on this material (cNS‐CNC‐0.15) demonstrated excellent volumetric capacitance performances (302 F cm^−3^ at 1 A g^−1^ and 243 F cm^−3^ at 50 A g^−1^) in aqueous KOH electrolyte (see Figure 15D**–**F for details). The greatly improved wettability of the carbon surface ensures fast ion/electron transfer with high electrochemical active surface area and low charge transfer resistance (<0.5 Ω) and fast response (0.47 s) (see Figure 15G for details). The stability was also excellent with retention rates of 93% after 100 000 cycles, and Coulomb efficiency of ≈100% (see Figure 15H for details). Compared with the materials reported in the literature, this material has high volumetric energy density and high average volumetric power density (see Figure 15I for details). This study provides an effective way (namely, N and S double doping combined with capillary compression) to develop high high‐performance carbon nanocage materials for high‐performance compact EDLCs with a wide range of practical applications.^[^ ^88^ ^]^

Amorphous 3D carbon framework (3DCF) structures with high surface area, layered porosity, and large conductive networks are considered as promising electrodes for EDLCs. Carbon nanocages with accessible active centers, thin carbon walls, and small cavities can provide high specific capacitance, shorten the ion transport length, and alleviate the stress caused by expansion and contraction during charging and discharging. The amorphous 3DCF constructed by continuous and conductive carbon nanocages network exhibited ultrafast charging and discharging rate and high energy density both in aqueous solution and ionic liquid electrolytes. In particular, it can deliver a high energy density of 34 Wh kg^−1^ at an ultrahigh power density of 150 kW kg^−1^ in EMIMBF_4_ at 4 V, which makes it a promising high performance EDLCs electrode.^[^ ^137^ ^]^ In addition, the development of hollow carbon materials is very important for the practical application of flexible supercapacitors. For example, the carbon nanofibers connected by porous ultrathin carbon nanocages can be used as flexible supercapacitors with a long cycle life. Even at a current density of 10 A g^−1^, the capacitance retention rate is up to 94.1% after 35 000 cycles. These excellent electrochemical properties can be attributed to the interconnected amorphous 3DCF structure of the carbon nanocages network.^[^ ^86^ ^]^

##### Graphene‐Like Carbon Nanocages for EDLCs

**Figure 16:**
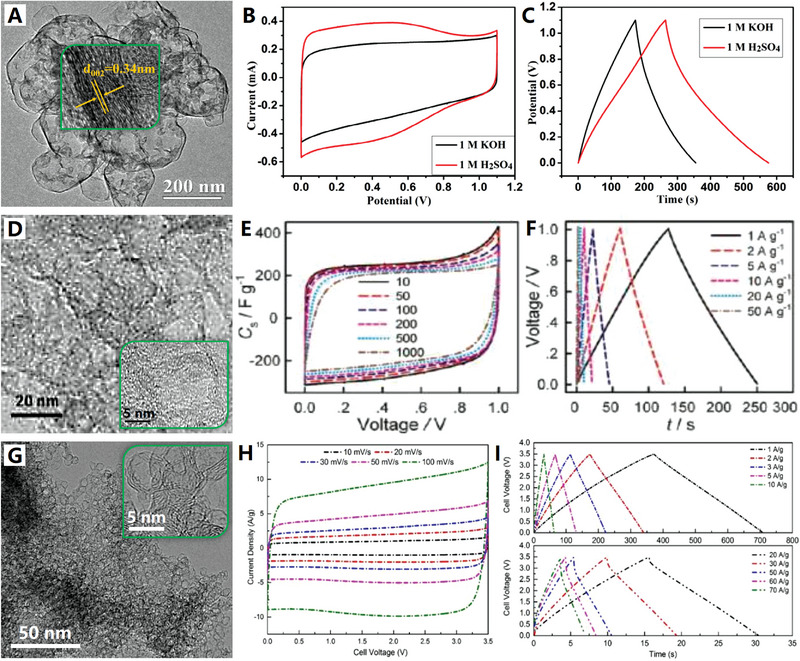
Graphene‐like carbon nanocages for high‐performance EDLCs. A–C) Hollow graphene‐like nanocages. Reproduced with permission.^[^ ^38^ ^]^ Copyright 2017, Elsevier. D–F) N‐doped graphene‐like carbon nanocages. Reproduced with permission.^[^ ^100^ ^]^ Copyright 2012, ACS. G–I) Carbon nanomesh composed of interconnected graphene‐like carbon nanocages. Reproduced with permission.^[^ ^125^ ^]^ Copyright 2018, Elsevier.

Hollow graphene‐like nanocages (HGNCs) materials, which have been successfully prepared on a large scale by an efficient quasi‐CVD method, have a typical hollow structure of ultrathin nanopores (<10 nm), a unique hierarchical porous structure with multiple mesoporous (2.5, 9.2, and 45 nm), and a wide distribution of macropores (50–200 nm) (see Figure 7H–K for details).^[^ ^38^ ^]^ Due to the good conductivity and graphene‐like nanostructures, the HGNCs electrode shows excellent electrochemical supercapacitance performance in both 1 m KOH and 1 m H_2_SO_4_ electrolytes. For KOH and H_2_SO_4_ electrolytes, the specific capacitances are 146 F g^−1^ and 283 g^−1^, respectively, when the current density is 1 A g^−1^. The HGNCs electrode exhibits better performance in 1 m H_2_SO_4_ electrolyte due to its pseudocapacitive form of oxygen‐containing groups. The results show that the electrode has good rate performance and good cycle stability, indicating that HGNCs is a promising electrode material for EDLCs (see **Figure** **16**A–C for details).^[^ ^38^ ^]^ Synthesis of N‐doped graphene‐like carbon nanocages (NGCNCs) with large surface area, high conductivity, and appropriate pore size distribution is an urgent need for high‐performance supercapacitor applications.^[^ ^100^ ^]^ The ultrathin NGCNCs based on polyaniline have excellent capacitive properties: high specific capacitance (248 F g^−1^ at 1.0 A g^−1^), excellent rate capacity (88% and 76% retention at 10 and 100 A g^−1^), and excellent cycle stability (95% retention after 5000 cycles) in aqueous solution of 6 m KOH. In addition to the large electrochemical double‐layer capacitance, polyaniline‐based NGCNCs also have a large pseudocapacitance in 0.5 m H_2_SO_4_ aqueous solution (see Figure 16D–F for details).^[^ ^100^ ^]^

It is an urgent need to explore graphene‐like carbon nanocages with excellent ratio capacitance in ionic liquid (IL) electrolytes for the development of next‐generation supercapacitor devices. A novel carbon nanomesh composed of interconnected high‐density graphene‐like carbon nanocages and ultrathin graphene shells, with large surface area and good mesoporous combined with its unique structural features, makes it a highly competitive high performance supercapacitor material in IL electrolytes (see Figure 16G–I for details).^[^ ^125^ ^]^ This unique carbon nanomesh morphology can provide a rapid ion diffusion pathway across the plane, and the graphene‐like carbon shell ensures the rapid transfer of electrons. At 1 A g^−1^, the capacitance is as high as 194 F g^−1^, at 70 A g^−1^, capacitance retention rate is 68%. In addition, thanks to the wide working voltage (3.5 V) and high ionic diffusion characteristics, even in 61.25 kW kg^−1^ of ultrahigh power density, the energy density can maintain at 56.1 Wh kg^−1^. Remarkably, the as‐assembled two‐electrode supercapacitors device can successfully power for light emitting diode (LED, 2.2 V) module (with 53 red LED lamps), showing its great potential of practical application in supercapacitor energy storage devices.^[^ ^125^ ^]^

##### Hollow Porous (Micro/Mesoporous) Carbon Nanospheres for EDLCs

**Figure 17:**
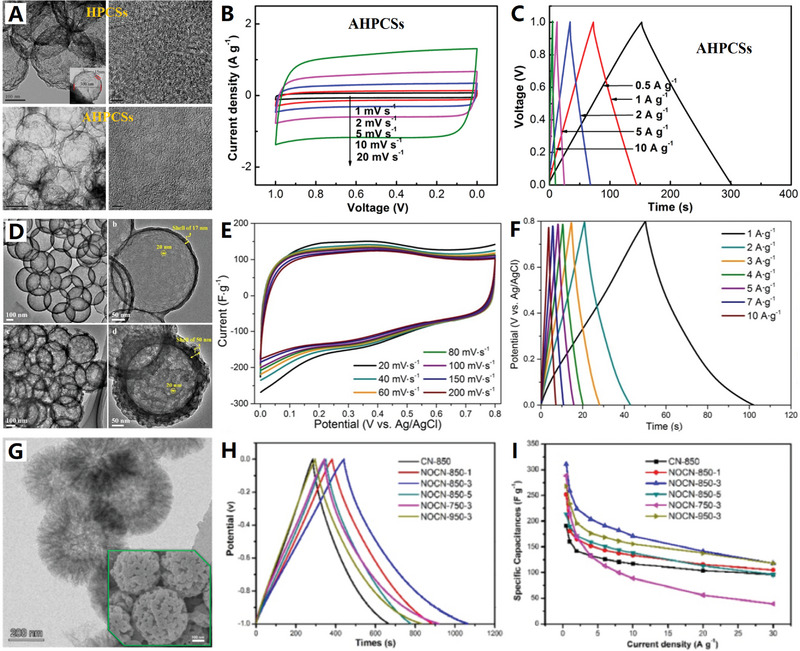
Hollow porous (micro/mesoporous) carbon nanospheres for high‐performance EDLCs. A–C) micro‐ and mesoporous hollow carbon nanospheres. Reproduced with permission.^[^ ^16^ ^]^ Copyright 2016, Elsevier. D–F) N‐doped large mesoporous (≈20 nm) hollow carbon nanospheres. Reproduced with permission.^[^ ^110^ ^]^ Copyright 2016, RSC. G–I) N,O codoped wrinkled carbon nanocages (mesoporous hollow carbon nanospheres). Reproduced with permission.^[^ ^172^ ^]^ Copyright 2021, Elsevier.

The hollow porous carbon spheres (HPCSs) with porous (micro‐ and mesoporous) carbon shell and macroporous cavity can be prepared by a convenient hydrothermal method with silica nanospheres as hard template and furfuryl alcohol as carbon source, and subsequent activation with potassium hydroxide. The HPCSs possess unique well‐balanced hierarchical porous structure with macropores, mesopores, and micropores combination, easy‐accessibly large surface area and high conductivity, which can provide fast ion transport kinetics and outstanding capacitive performance. A high specific capacitance of 240.0 and 303.9 F g^−1^ at 1 A g^−1^ was demonstrated for the HPCSs and activated HPCSs (AHPCSs), respectively. The two‐electrode device also delivered a high specific capacitance of 74.5, 71.5, 67.4, 61.0, and 51.0 F g ^−1^ at current densities of 0.5, 1, 2, 5, and 10 A g^−1^, respectively, indicating that the micro‐ and mesoporous hollow carbon nanospheres are a promising electrode material for high‐performance EDLCs (see **Figure** **17**A–C for details).^[^ ^16^ ^]^ The N‐doped hollow carbon nanospheres with thickness‐tunable large mesoporous (≈20 nm) shells were successfully synthesized for the first time by using the colloidal silica and the diblock copolymer PS‐*b*‐PEO as the dual‐template and dopamine as the precursor. The unique structural properties enable them to be promising materials as electrode materials for supercapacitor applications (see Figure 17D–F for details).^[^ ^110^ ^]^

Recently, Prof. Qin research group from Guilin University of Technology and Prof. Tan research group from Guangdong University of Petrochemical Technology cooperated to synthesize N,O codoped wrinkled carbon nanocages (mesoporous hollow carbon nanospheres) for the first time by using dendrite fiber nanosilica spheres as sacrifice‐template, glucose as carbon source and melamine as nitrogen source (see Figure 17G–I for details).^[^ ^172^ ^]^ The carbon nanocage has high specific surface area (813 m^2^ g^−1^), high nitrogen (16.24 at%) and oxygen (5.36 at%) content, high specific capacitance (311.1 F g^−1^ at 0.5 A g^−1^ and 118.1 F g^−1^ at 30 A g^−1^), and good cycling performance (97.6% capacitance retention after 10 000 cycles at 10 A g^−1^). Assembled as a button‐symmetric cell, the mesoporous hollow carbon nanospheres also have an energy density of 12.3 Wh kg^−1^ at a power density of 175 W kg^−1^. Based on these, the unique mesoporous structure and N,O codoping characteristic endue the carbon nanocage electrode material a potential application in supercapacitors. In general, these hollow carbon nanospheres (microporous or mesoporous structures) constructed from silica hard template strategies^[^ ^108^, ^109^, ^110^, ^111^, ^112^, ^113^, ^114^, ^115^ ^]^ can exhibit different electrochemical capacitance properties, due to their different specific surface area and varying pore size or doping structures.

#### 5.1.2 Faraday PCs

The electrode materials of Faraday pseudocapacitors include transition metal oxides, hydroxides, sulfide, etc. These materials have the problem of low conductivity, which leads to the low‐rate performance and poor cycle stability when charging and discharging under large current. Therefore, researchers usually improve the rate and cycle properties of pseudocapacitive materials by means of carbon‐supporting composite design. Hollow carbon nanocages are an ideal carbon carrier material for improving the performance of pseudocapacitive material and the overall electrode performance. A previous study found that ultrathin MnO_2_ nanofibers grown vertically on the outer surface of graphited hollow carbon spheres (GHCS) yielded composite electrode materials with good electron transport, fast ion penetration, fast reversible Faraday reaction, and excellent rate performance.^[^ ^59^ ^]^ The asymmetric supercapacitor with GHCS‐MnO_2_ as the positive electrode and GHCS as the negative electrode can be reversibly charged/discharged at a maximum battery voltage of 2.0 V in 1.0 mol L^−1^ Na_2_SO_4_ aqueous electrolyte, which demonstrated a much higher energy density (22.1 Wh kg^−1^) than the GHCS symmetric supercapacitor (2.6 Wh kg^−1^). Recently, a new type of hollow carbon microsphere/MnO_2_ nanosheet composite was prepared by in situ self‐limiting deposition under hydrothermal conditions, and its electrochemical capacitor performance was also studied. The MnO_2_ nanosheet grows on the surface of the carbon microsphere and forms a loose‐packed morphology. As the electrode layer, it can reduce the quality requirements of MnO_2_ and obtain high specific capacitance of 239 F g^−1^ at current density is 5 mA cm^−2^, which would be a strong promise for high‐rate electrochemical pseudocapacitive energy storage applications.^[^ ^173^ ^]^

**Figure 18:**
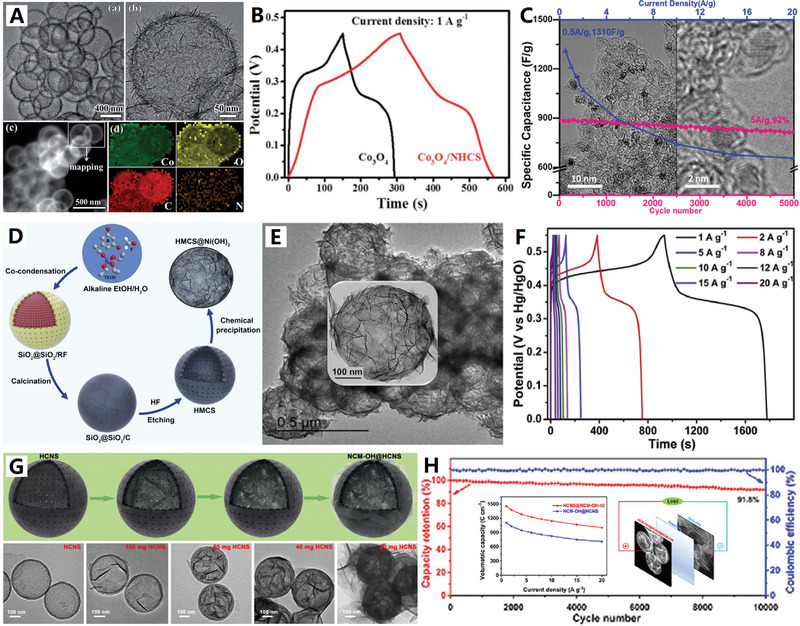
Hollow carbon nanocages for high‐performance Faraday pseudocapacitors. A,B) Co_3_O_4_ nanosheets supported on N‐doped carbon hollow spheres. Reproduced with permission.^[^ ^174^ ^]^ Copyright 2018, Wiley. C) Small Co_3_O_4_ nanoparticles fixed inside carbon nanocages. Reproduced with permission.^[^ ^81^ ^]^ Copyright 2020, ACS. D–F) Ni(OH)_2_ nanosheets on hollow carbon nanocages enwrapped construction. Reproduced with permission.^[^ ^175^ ^]^ Copyright 2018, Elsevier. G,H) Ni–Co–Mn hydroxide nanosheets@hollow carbon nanocages ship‐in‐bottle construction. Reproduced with permission.^[^ ^176^ ^]^ Copyright 2021, Elsevier.

In 2018, Yu and colleagues prepared Co_3_O_4_ nanosheets/N‐doped carbon hollow spheres (Co_3_O_4_/NHCSs) composite electrodes with hierarchical structure by hydrothermal and calcination methods (see **Figure** **18**A,B for details).^[^ ^174^ ^]^ The specific capacitance of the Co_3_O_4_/NHCSs composite electrode reached 581 F g^−1^ at 1 A g^−1^, and the retention rate reaches 91.6% at 20 A g^−1^, which is much better than that of the single Co_3_O_4_ electrode (318 F g^−1^ at 1 A g^−1^, 67.1% at 20 A g^−1^). In addition, using the composite as a positive electrode to fabricate an asymmetric supercapacitor, the device can provide a high energy density of 34.5 Wh kg^−1^ at a power density of 753 W kg^−1^ and show good cycle stability. All these attractive results make the Co_3_O_4_/NHCSs composite structure a promising electrode material for high performance Faraday pseudocapacitors. In 2020, Yang et al. used the P,N codoped carbon matrix (dense small‐diameter carbon nanocages) of ultrahigh specific surface area and abundant nanocavity as a novel host to limit the growth of Co_3_O_4_ nanoparticles, and successfully prepared extremely fine Co_3_O_4_ active capacitive materials (see Figure 18C for details).^[^ ^81^ ^]^ The small Co_3_O_4_ nanoparticles (less than 2 nm in particle size) are firmly fixed inside the carbon nanocages, thus improving the capacitance performance (1310 F g^−1^ at 0.5 A g^−1^) and ensuring the long‐term stability (92% capacitance retention after 5000 cycles at 5 A g^−1^) of the composite. In addition, the asymmetric supercapacitor assembled using the composite as positive electrode material and activated carbon as negative electrode material can provide a high energy density of 47.18 W h kg^−1^ at 375 W kg^−1^, demonstrating the application prospect for practical supercapacitor devices.

The hollow carbon nanocages (namely, hollow mesoporous carbon spheres)‐built “ship‐in‐bottle” composite nanostructures manifested excellent electrochemical performances (high activity and high stability), due to their unique interior constructions and extra confined functionalities (see Figure 12 for details).^[^ ^157^, ^158^, ^159^, ^160^, ^161^ ^]^ In 2018, Fu et al. proposed a simple chemical precipitation method to prepare hierarchical nanocomposites of hollow mesoporous carbon spheres enwrapped by 3D Ni(OH)_2_ ultrathin nanosheets (see Figure 18D–F for details).^[^ ^175^ ^]^ This hollow carbon nanocages@Ni(OH)_2_ nanosheets hierarchical core–shell construction showed high specific capacity (844 C g^−1^ at 1 A g^−1^), high rate performance (515 C g^−1^ at 10 A g^−1^), and good cycle stability (91.4% retention after 10 000 cycles at 10 A g^−1^). Although hollow nanocage nanostructures have great advantages as electrodes for supercapacitors, the huge cavity inside nanocages seriously hinders the improvement of volumetric performances. In 2021, Zhou et al. successfully constructed a hierarchical spherical nanostructure by confining Ni–Co–Mn hydroxide nanosheets mostly to the interior of hollow carbon nanospheres (with a few nanosheets loading on the exterior) (see Figure 18G,H for details).^[^ ^176^ ^]^ This unique Ni–Co–Mn hydroxide nanosheets@hollow carbon nanocages ship‐in‐bottle construction significantly improved the electrical conductivity and structural stability of the hydroxide nanosheets and greatly increased the volumetric specific capacity of the electrode material (1455.2 C cm^−3^ at 1 A g^−1^). Particularly, the assembled hybrid supercapacitors exhibited high specific energy (44.9 Wh kg^−1^ at the 793.5 W kg^−1^) and ultrahigh stability (high capacitance retention ratio of 91.8% after 10 000 cycles). The hollow carbon nanocages showed unique interior spatial confinement effect and outer surface‐confined effect, which can be used to design high‐performance multifunctional composite nanomaterials.

#### 5.1.3 Metal‐Ion HCs

HCs with metal ions (such as lithium, sodium, potassium, and zinc ions) have gained wide attention in recent years, because they combine the advantages of conventional metal‐ion batteries and double‐layer capacitors to achieve a balance between power and energy output.^[^ ^177^ ^]^ The main challenge of metal‐ion HCs is that the power density and long‐term stability of battery‐type anodes are generally not ideal because of the poor kinetic and structural stability of metallic materials. Recently, Li and collaborators synthesized manganese oxide embedded in hollow carbon nanoboxes anode materials (MnO@HCNB) by salt template‐assisted chemical vapor deposition for sodium‐ion HCs.^[^ ^178^ ^]^ The MnO particles were ≈40 nm, coated with 3 nm carbon on the surface (forming MnO@C yolk–shell structure), and uniformly pinned into hollow carbon nanoboxes with a wall thickness of ≈15 nm. For sodium ion storage, the yolk–shell structure can not only reduce the internal stress in the process of sodium storage, but also limit the MnO conversion reaction inside the carbon shell, which induces a large number of Mn/Na_2_O interfaces and reduces the diffusion barrier of sodium ions. The sodium ion HCs assembled with active carbon (MnO@HCNB//AC) can provide a maximum energy density of 116 Wh kg^−1^ and a power density of 4.2 kW kg^−1^.^[^ ^178^ ^]^ Tin disulfide (SnS_2_) is a promising anode material for capacitive sodium storage. The SnS_2_ nanosheets constrained by hollow carbon materials, such as the SnS_2_ nanosheets confined in CNBs and hollow CNSs, have shown better sodium storage performances in terms of high specific capacity, good cycle stability, and good rate performances.^[^ ^161^ ^]^

In 2021, Zhao et al. used manganese carbonate as a precursor to obtained multicavity PCS with enhanced reaction kinetics as anode materials for high‐performance potassium‐ion HCs.^[^ ^132^ ^]^ The carbon sphere has a highly open 3D channel, which is conducive to the transport of potassium ions and increases the contact area between electrode and electrolyte. The subsequent P,O codoping treatment, while maintaining the 3D structure of porous carbon spheres, introduced a large number of doping atoms and defective active sites, thus greatly improving the reaction kinetics based on surface adsorption. Since the ionic size and mass of K^+^ is much larger than that of Li^+^ and Na^+^, it is difficult to identify anode materials with high capacity and rate capabilities. In 2022, Wang et al. reported that the pyridine‐rich nitrogen hollow carbon nanospheres (NHCNs) with tunable shell thickness can be prepared by core–shell polymerization and carbonization.^[^ ^93^ ^]^ By simply adjusting the shell thickness, it is possible to control the surface area and defect/functional group levels of carbon nanospheres, which are critical in determining their potassium storage capacity. In addition, the kinetically matched NHCNs//porous carbon potassium‐ion HCs combined the high energy and power density, demonstrating the promise of using NHCNs for potassium storage.

Zinc‐ion HCs combine the superpower of supercapacitors with the high energy of batteries, making them a highly competitive candidate power source for current water‐based energy storage systems. Using ZIF‐8 as a platform, Zhu et al. constructed the 3D skeleton of N‐doped carbon nanocages by pyrolysis and activation of polypyrrole, and achieved porosity and high N‐doping content at the same time.^[^ ^17^ ^]^ Due to the comprehensive regulation of morphology, pore structure, and surface chemistry, the obtained carbon nanocages showed a significant enhancement in terms of active center exposure and diffusion kinetics. Therefore, the zinc‐ion HCs with this carbon cathode achieve a significantly increased zinc‐ion storage capacity. Xu and co‐workers designed a solid‐state gas‐steamed metal–organic framework approach to fabricate carbon nanocages with controlled openings on walls, and N,P dopants.^[^ ^179^ ^]^ Due to the advantage of large openings on their walls for enhanced kinetics of mass transport and N,P dopants within the carbon matrix for favoring chemical adsorption of Zn ions, when used as carbon cathodes for aqueous Zn‐ion HCs, such open carbon nanocages display a wide operation voltage of 2.0 V and an enhanced capacity of 225 mAh g^−1^ at 0.1 A g^−1^, as well as ultralong cycling lifespan of up to 300 000 cycles with 96.5% capacity retention.

### 5.2 Application Case Analysis of Hollow Carbon Nanocage for Rechargeable Batteries

Among various forms of carbon‐based materials, hollow carbon materials have been widely studied as electrode materials for rechargeable batteries due to their unique hollow structure, controllable pore size distribution, high specific surface area, high electrical conductivity, and excellent chemical and mechanical stability.^[^ ^180^ ^]^ The specific structural advantages of hollow carbon nanocages in battery applications include the following. 1) The hollow inner cavity can provide a buffer space for the volume change of the electrode, and promote the electrolyte penetration, which is conducive to shorten the electron transport/ion diffusion and enhance the kinetic process. 2) The porous sphere wall can provide abundant active sites for charge storage, and the doping structure can improve the interfacial contact and wettability with electrolyte, thus improving the capacity and rate performance of the battery. 3) The well cage‐like structure gives the electrode high mechanical stability, which can effectively inhibit the material volume deformation, so as to achieve excellent cyclic stability.

#### 5.2.1 Lithium‐Ion Batteries (LIBs)

LIBs are the most commonly used rechargeable batteries. Improving the energy density and cycle life of LIBs is of great significance for the further development of the new energy industry. Carbon materials with controllable structures are considered as potential anode materials for LIBs due to their desirable advantages. Carbon material has the advantages of low cost, environmental friendliness, good conductivity, long cycle life, and so on, which is considered as the most excellent anode material for LIBs.^[^ ^28^ ^]^ Unfortunately, commercial carbon materials, limited by their theoretical capacity of 372 mAh g^−1^, cannot meet the high energy density requirements of LIBs. In recent years, many efforts have been made to develop new carbon materials (such as hollow carbon nanocages and their composites) for better energy storage performances.^[^ ^84^, ^142^, ^158^ ^]^

**Figure 19:**
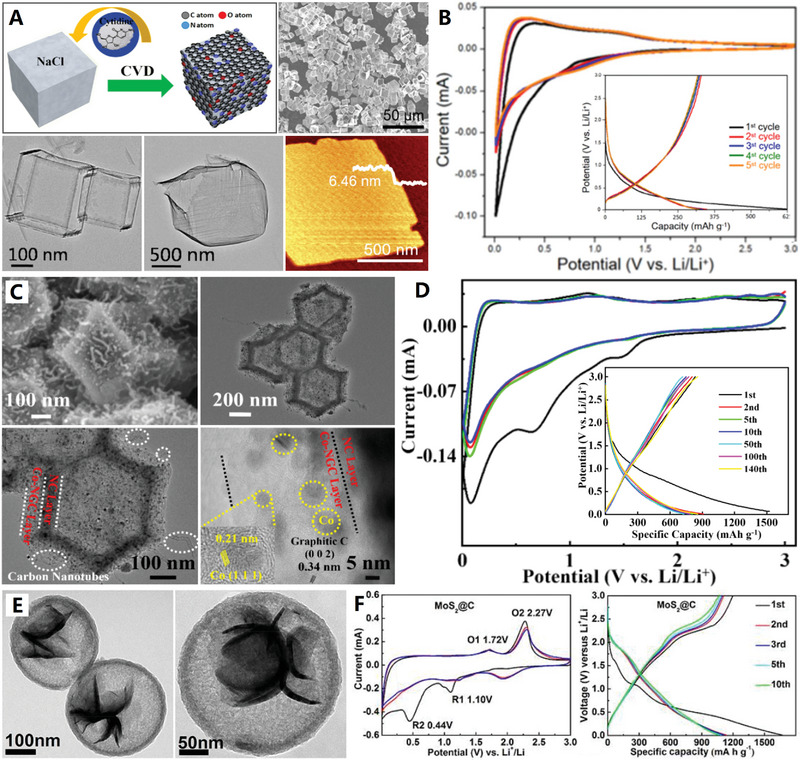
Hollow carbon nanocages for high‐performance Li‐ion batteries. A,B) N,O codoped graphene‐like carbon nanocages (hollow cubes). Reproduced with permission.^[^ ^84^ ^]^ Copyright 2020, Elsevier. C,D) Co nanoparticles embedded in CNTs‐decorated polyhedral carbon nanocages. Reproduced with permission.^[^ ^142^ ^]^ Copyright 2020, Wiley. E,F) 3D flower‐like MoS_2_ nanosheets@hollow carbon nanocages ship‐in‐bottle construction. Reproduced with permission.^[^ ^158^ ^]^ Copyright 2017, ACS.

For example, in 2020, Sun et al. synthesized N,O codoped graphene‐like carbon nanocages (NOGCN) as anodes for LIBs by a one‐step process on water‐soluble NaCl nanocrystals from biomass cytidine (the reactants used are fully renewable and readily available) (see **Figure** **19**A,B for details).^[^ ^84^ ^]^ The heterogeneous element doping, controlled layer spacing, and favorable self‐supported nanocage structure greatly promote electrolyte penetration and improve the kinetics of ion and electron transport, resulting in excellent electrochemical performances. The synthesized NOGCN electrode has a high Li storage capacity of 620 mAh g^−1^ over 500 cycles at 500 mA g^−1^, and a continuous amplification capability. Kinetic analysis and density functional theory calculations detail the Li absorption properties of the N,O‐doped graphene‐like structure, further demonstrating its chemical affinity and advantage for Li storage. This study provides an efficient and green method for the general preparation of hollow carbon nanocages (hollow cubes) anode materials for LIBs.

In 2020, through the pyrolysis of the double‐layer MOF structure (ZIF‐8@ZIF‐67), Zhang and co‐coworkers obtained the N‐doped core–shell carbon nanocages modified by Co nanoparticles and N‐doped graphitic carbon nanotubes (Co‐NGC@NC), which were used as anode materials for high‐performance LIBs (see Figure 19C,D for details).^[^ ^142^ ^]^ The anode material exhibits a specific capacity of 1580 mAh g^−1^ at a current density of 100 mA g^−1^, and retains a specific capacity of 950 mAh g^−1^ after 140 cycles. It also has excellent cycle stability and rate performance. Co‐NGC@NC has a robust nanocage structure, and its surface carbon nanotubes and Co nanoparticles in the material can effectively improve the electronic conductivity of the material. The material has rich N‐doped structure, porous and hollow structure, so the site and space for Li insertion are rich, and it is easy to obtain a large pseudocapacitance in the charging and discharging process. The special structure of Co nanoparticles embedded polyhedral carbon nanocages endows with excellent electrochemical performances, so they can be used as anode materials for high‐performance LIBs.

Typical nanosheet materials of MoS_2_, due to their adjustable crystal structure and nanostructure, often exhibit excellent lithium storage properties (theoretical capacity is almost twice that of graphite). However, the rapid capacity decay and poor rate performance limit the application of MoS_2_, and carbon coating is one of the most effective and direct methods to improve the electrochemical performances. The ship‐in‐bottle yolk–shell structured nanomaterials can enhance the energy density of LIBs by increasing the mass fraction of active substances, and the gap between yolk and shell can be used as the volume expansion buffer of active substances when lithium is removed. Chen and co‐workers developed the 3D MoS_2_ nanosheets enveloped inside hollow mesoporous carbon spheres (MoS_2_@C) as high‐performance cathode material for the LIBs (see Figure 19E,F for details).^[^ ^158^ ^]^ The first discharge and charging capacity of the core–shell MoS_2_@C are 1671 and 1197 mAh g^−1^, which are higher than those of C@MoS_2_ (1337 and 866 mAh g^−1^) and pure MoS_2_ (813 and 580 mAh g^−1^). The excellent electrochemical performance of MoS_2_@C with ship‐in‐bottle construction can be attributed to the following aspects. 1) The high specific surface area and pore structure of carbon shell facilitate the effective penetration of electrolyte in nanocages, and bring large electrode/electrolyte contact area for the rapid diffusion and migration of Li^+^. 2) The yolk–shell construction provides sufficient buffer space for the volume expansion of MoS_2_ in the process of lithium insertion and deintercalation, which prevents the material from falling off from carbonaceous material. 3) The curved carbon shell as the whole skeleton significantly improves the structural integrity of the material, thus ensuring the cycle stability and rate performance required by the electrode materials.^[^ ^158^ ^]^

#### 5.2.2 Sodium‐Ion Batteries (SIBs)

**Figure 20:**
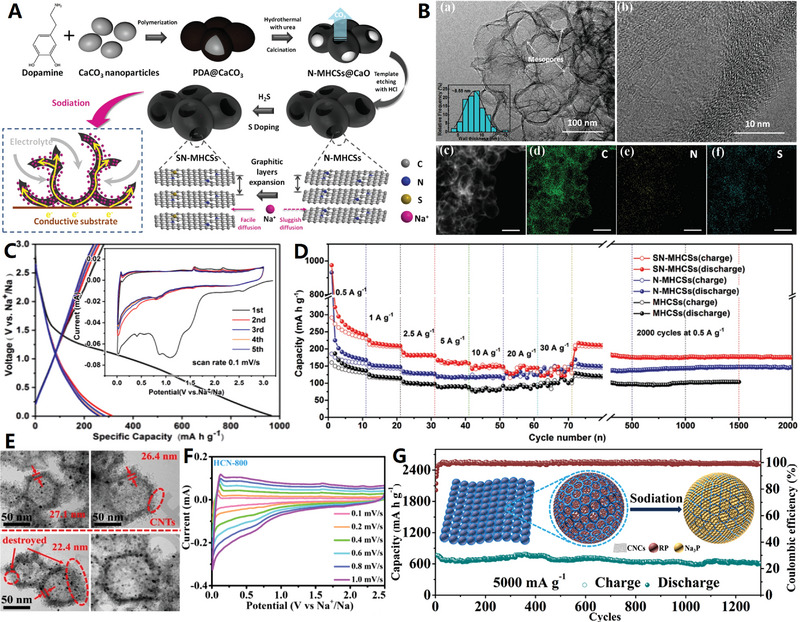
Hollow carbon nanocages for high‐performance Na‐ion batteries. A–D) Mesoporous hollow carbon nanocages codoped with S and N. Reproduced with permission.^[^ ^181^ ^]^ Copyright 2019, Wiley. E,F) Co nanoparticles embedded in polyhedral carbon nanocages. Reproduced with permission.^[^ ^14^ ^]^ Copyright 2021, Elsevier. G) Red phosphorus (RP) embedded in dense carbon nanocages (CNCs) conductive network. Reproduced with permission.^[^ ^182^ ^]^ Copyright 2021, ACS.

Compared with LIBs, SIBs have the advantages of abundant resources and low price, so they have high application potential as a new generation of energy storage batteries. The development of electrode materials with high rate and long cycle performance is an urgent problem to be solved in the application of SIBs. Among them, hollow carbon anode materials show a high commercial prospect due to their high performance, low cost, and environmental friendliness. Recently, a team of Prof. Sun from Beijing Institute of Technology prepared mesoporous hollow carbon nanocages codoped with S and N using polydopamine as carbon source and calcium carbonate as template for application of SIBs (see **Figure** **20**A–D for details).^[^ ^181^ ^]^ The material has interconnected hollow mesoporous structure with a diameter of 70–100 nm, and the size of mesoporous is ≈20–30 nm, N and S are evenly doped in carbon. When used as the negative electrode of SIBs, it shows excellent rate performance and cycle stability, and after long‐term cycle at high rate, the carbon layer spacing increases, the arrangement uniformity increases, and the capacity increases. At the current density of 0.5, 1.0, 2.5, 5, 10, 20, and 30 A g^−1^, the steady capacity is 240, 208, 180, 157, 147, 144, and 138 mAh g^−1^, respectively. After 2000 cycles of 0.5 A g^−1^, the capacity is 184 mAh g^−1^. This study solves the problem of slow diffusion rate caused by sodium ions with large radius, and buffers the significant electrode volume change, greatly improves the material ratio and cycle stability, and promotes the practical application of carbon materials in SIBs.

Hollow nanostructure‐based composites show great potential in sodium ion energy storage and have attracted much attention. Recently, Zhang et al. reported a series of HCNs embedded with cobalt (Co) nanoparticles prepared by charring ZIF‐8@ZIF‐67 core–shell precursor systems at various temperatures (700, 800, and 900 °C) for application of SIBs (see Figure 20E,F for details).^[^ ^14^ ^]^ It is found that when the carbonization temperature is 800 °C, HCN‐800 (with Co nanoparticles) has good hollow structure, thin shell, and large specific surface area, and contains abundant micropores and mesoporous. When used as anode material for SIBs, it can achieve a reversible capacity of 111.7 mAh g^−1^ (after 10 000 cycles) even at a current density of 10.0 A g^−1^, with good cycle stability. In addition, the kinetics behavior of sodium storage was analyzed by CV technique: the HCN‐800 (with Co nanoparticles) had good capacitance characteristics (80.4% capacitance contribution). A complete cell was assembled with LiCoO_2_/Na_3_V_2_(PO4)_3_ cathode and HCN‐800 (with Co nanoparticles) anode, and its cycle performance was investigated. The reversible capacity of 164.7 mAh g^−1^ or the SIBs can be obtained after 100 cycles at 0.1 A g^−1^ current density, which demonstrated the potential for practical application of SIBs.

Due to its extremely high theoretical capacity, appropriate operating voltage, and abundant reserves, red phosphorus (RP) has attracted much attention as a promising candidate negative material for the NIBs with high energy density (2595 mAh g^−1^, Na_3_P). However, poor conductivity (≈10^−4^ S cm^−1^) with low conductivity and large volume changes can lead to poor rate performance and cyclability of RP, which severely limits its practical use in NIBs. Recently, Yu and Hu jointly reported that by trapping [ethylenediamine‐P*_n_*]—([EN‐P*_n_*]—), the marginal micropores in carbon nanocages (CNCs) are conducive to the nucleation and growth of P*_n_* clusters, and nanoscale RP can be encapsulated into conductive CNCs (RP@CNCs) network by a simple phosphoamine method combined with an evacuation–filling process (see Figure 20G for details).^[^ ^182^ ^]^ The large intracellular volume of CNCs and controllable filling method make the RP@CNCs composite with ultrahigh RP loading (85.3 wt%). Thanks to the synergistic effect of high structural stability and fast electron transport of the intracellular and conductive network. The RP@CNCs composite has a high capacity of 1363 mAh g^−1^ after 150 cycles at 100 mA g^−1^, excellent rate performance, and long cycle performance, with a capacity retention rate of more than 80% after 1300 cycles at 5000 mA g^−1^. This prototypical design promises an efficient solution to maximize RP loading within carbon nanocages as well as to boost the electrochemical performance of RP‐based anodes for NIBs.

#### 5.2.3 Potassium‐Ion Batteries (PIBs)

PIBs have attracted extensive attention due to their abundant potassium resources and similar performance to lithium/sodium ion batteries. More importantly, the redox potential of K^+^ is lower than that of Na^+^ and close to that of Li^+^, so PIBs can operate under high pressure and provide high energy density.^[^ ^78^ ^]^ The development of nanostructured carbon materials with hollow and porous structures is very beneficial to accommodate abundant potassium ions, which can allow volume expansion during multiple cycles and shorten the diffusion path of ions, thus improving the rate performance and long‐term stability.^[^ ^139^ ^]^ Graphite materials cannot store sodium effectively by electrochemical reaction, but they show high potassium storage activity, because K^+^ can form intercalated compounds with graphite through intercalation reaction. When the first‐order intercalated compound KC_8_ is formed, it can have a theoretical specific capacity of 279 mAh g^−1^.

**Figure 21:**
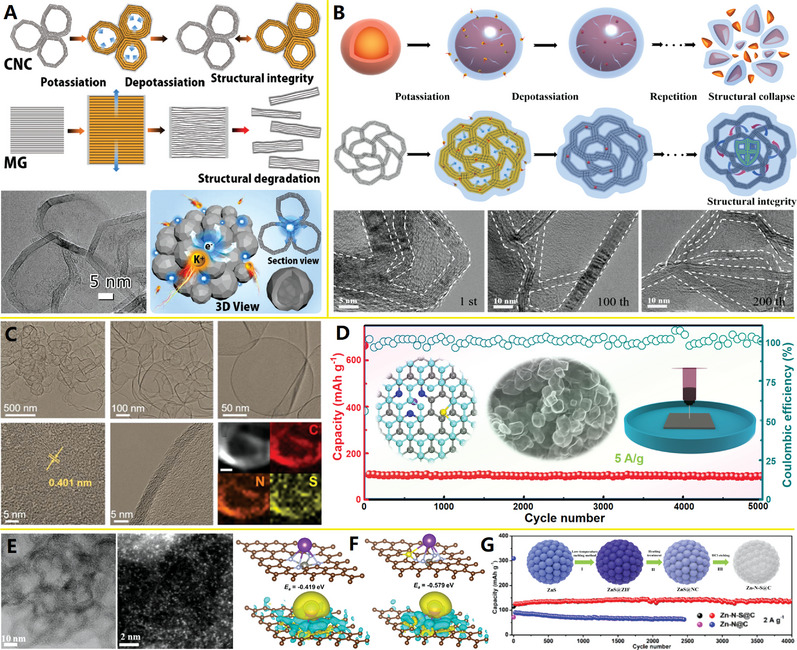
Hollow carbon nanocages for high‐performance K‐ion batteries. A) Nonopen layered graphite carbon nanocages. Reproduced with permission.^[^ ^22^ ^]^ Copyright 2018, Wiley. B) Cross‐linked hollow graphite carbon nanocages. Reproduced with permission.^[^ ^72^ ^]^ Copyright 2021, RSC. C,D) Heteroatom dual‐doped graphene‐like hollow nanocages. Reproduced with permission.^[^ ^138^ ^]^ Copyright 2020, Wiley. E–G) Metal single atoms‐doped multicavity graphene‐like carbon nanocages. Reproduced with permission.^[^ ^183^ ^]^ Copyright 2021, Elsevier.

According to the shortcomings of traditional graphite materials, such as high anisotropy and weak interlayer interaction, Song and collaborators recently proposed to use CNCs with high graphitization as anode materials for PIBs (see **Figure** **21**A for details).^[^ ^22^ ^]^ The results show that small particle size mesophase graphite (MG) with open lamellar structure will lead to the peeling of carbon layer due to the large interlayer variation in the process of potassium storage, which leads to the destruction of lamellar structure and the degradation of cycle performance. The CNC has high structural stability and peeling resistance. It can keep the structure stable in the process of potassium storage, so it has excellent potassium storage cycle performance. The excellent performance of CNC is mainly attributed to its unique nanocages structure. 1) Due to the concentric arrangement of carbon layers, the cage‐like structure can effectively reduce the anisotropy and avoid the slip between carbon layers to ensure the integrity of the layered structure. 2) The hollow structure is also conducive to buffer the stress of K^+^ during intercalation/deintercalation to ensure the structure integrity. This study reveals the advantages of carbon with nonopen layered structure (graphite carbon nanocages), which provides a new idea for the structural design of carbon anode materials for PIBs.^[^ ^22^ ^]^

Recently, Lu and colleagues used ascorbic acid and ferric chloride hexahydrate to synthesize an ultrastable structure of cross‐linked hollow graphite carbon (HGC) nanocages through a simple catalytic graphitization process to achieve stable potassium storage (see Figure 21B for details).^[^ ^72^ ^]^ During the annealing process, the resulting Fe catalyst is graphitized with the carbon source, and the graphite layer grows under high temperature carbonization to form a 3D cross‐linked structure. The Fe particles were then removed by etching with hydrochloric acid to form a tightly cross‐linked hollow structure. The HGC material has the following advantages. 1) The preparation process of cross‐linked HGC nanocages anode is simple and rapid, which meets the requirements of low cost efficiency and mass production. 2) The cross‐linked HGC nanocages forms a compact network structure, which allows the rapid transfer of electrons between different carbon frames, promotes the reaction kinetics and fast transport of K^+^, and achieves good electrical conductivity. 3) Thanks to the superstability of cross‐linked HGC structure, the HGC potassium storage anode with aluminum foil as a collector achieves high reversible capacity and excellent stability. The hollow structure of HGC with cross‐linked structure is different from the hollow sphere. The hollow sphere is prone to cracking and crushing during continuous charging and discharging processes, while the cross‐linked skeleton of HGC has good traction force to maintain the stability and integrity of the structure.^[^ ^72^ ^]^

In 2020, Lu et al. used low‐temperature PECVD strategy to accurately synthesize NSG hollow nanocages for PIBs (see Figure 21C,D for details).^[^ ^138^ ^]^ The NSG has uniform N/S dual doping, abundant potassium‐philic surface molecules, efficient electron/ion transport paths, and enhanced electrode–electrolyte interactions, which are important for high‐rate performance and extended cycle life. The hollow structure of NSG makes it structurally stable, which can adapt to the volume change of graphite layer in the process of charging and discharging. As a result, NSG hollow nanocages showed high potassium storage capacity, including good rate capacity (≈100 mAh g^−1^ at 20 A g^−1^) and excellent cycle stability (90.2% capacity retention after 5000 cycles at 5 A g^−1^). Density functional theory (DFT) simulations were used as a theoretical guide to verify the advantages of N/S dual doping in terms of K ion adsorption/diffusion and total conductivity. Finally, the construction of high‐loading NSG electrode is realized by printing technology, which brings a broad prospect for the application of PIBs with high performance and low cost in the future.^[^ ^138^ ^]^

To sum up, the graphited carbon materials with hollow structures have been shown to effectively alleviate volume expansion and accelerate the diffusion of K ions, thus improving the rate performance and long‐term stability.^[^ ^22^, ^72^ ^]^ At the same time, doping nonmetallic atoms in the carbon frame has been shown to change the electronic structure and increase the storage sites of K ions to improve its K storage performances.^[^ ^138^ ^]^ In 2021, Wu and co‐workers demonstrated that the simultaneous doping of nonmetallic (N and S) and metallic (Zn) single atoms on multicavity graphene‐like carbon nanocages (Zn–N–S@C) can greatly improve the K storage performances of PIBs (see Figure 21E–G for details).^[^ ^183^ ^]^ Thanks to the S‐coordinated Zn–N_4_ structure in the hollow carbon sphere, as the cathode material of PIBs, Zn–N–S@C achieves high capacity and excellent cycle stability of 350 mAh g^−1^ at 0.1 A g^−1^, and the capacity retention rate of 4000 cycles at 2 A g^−1^ is 92%. Its capacity and cycling performance are better than that of the sulfur‐free Zn–N@C sample. The DFT calculation shows that the Zn single atom can be used as an additional storage site for electrons and enhance the reactivity of surrounding anions during potassiation and depotassiation. In addition, the binding of S/N codopants to the Zn single atom in the carbon skeleton of nanocages can regulate the electronic structure and enhance the adsorption of K^+^, thus enhancing the K storage capacity. This work reveals new directions for achieving high‐performance carbon nanocage materials for energy storage applications by modulating the coordination chemistry of metal single atoms.^[^ ^183^ ^]^

#### 5.2.4 Lithium–Sulfur Batteries (LSBs)

**Figure 22:**
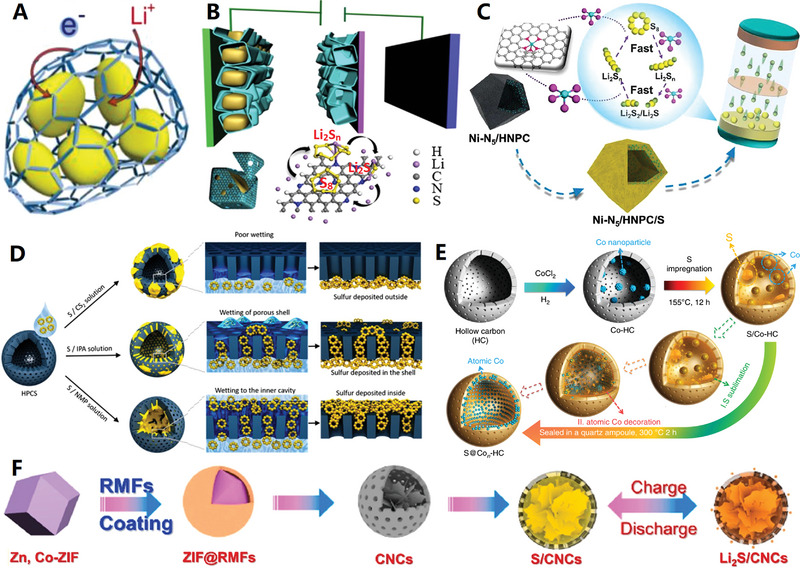
Hollow carbon nanocages for high‐performance Li–S batteries. A) S@graphene‐like carbon nanocages. Reproduced with permission.^[^ ^186^ ^]^ Copyright 2016, Wiley. B) S@cubic carbon nanocages. Reproduced with permission.^[^ ^187^ ^]^ Copyright 2019, Elsevier. C) S@polyhedral carbon nanocages. Reproduced with permission.^[^ ^188^ ^]^ Copyright 2020, ACS. D) S@hollow porous carbon spheres. Reproduced with permission.^[^ ^189^ ^]^ Copyright 2021, National Acad Sciences. E) S@Co*_n_*‐hollow porous carbon spheres. Reproduced with permission.^[^ ^190^ ^]^ Copyright 2021, Nature. F) S@carbon nanosheets/porous carbon spheres ship‐in‐bottle construction. Reproduced with permission.^[^ ^191^ ^]^ Copyright 2021, Elsevier.

LSBs have high theoretical energy density (2600 Wh kg^−1^) and specific capacity (1675 mAh g^−1^), which are recognized as a promising new generation of electrochemical energy storage technology.^[^ ^184^ ^]^ However, due to the shuttle effect of positive polysulfide and slow redox conversion kinetics, low electronic conductivity and poor electrochemical cycle stability of sulfur electrodes have severely limited their commercial development. The rational design light‐quality and high‐stability carbon materials with high loading capacity and high catalytic activity (such as foreign active component‐functionalized hollow carbon nanocages), can simultaneously suppress the shuttle effect of polysulfide (via physical and chemical associated adsorption) and improve the redox conversion kinetics (via catalytic conversion of additional active sites) for LSBs.^[^ ^185^ ^]^ The different morphologies (graphene‐like nanocages, cubic or polyhedral nanocages, and spherical nanocages), different porous structures (microporous and/or mesoporous structures), and different interior structures (hollow or ship‐in‐bottle structures) of carbon nanocages can have different effects on sulfur adsorption and polysulfide conversion (see **Figure** **22** for details).^[^ ^186^, ^187^, ^188^, ^189^, ^190^, ^191^ ^]^

As early as 2016, Guo's group developed a unique graphene‐like carbon nanocage and used it as a carrier of sulfur in high‐rate and long‐life LSBs (see Figure 22A for details).^[^ ^186^ ^]^ The carbon nanocages are surrounded by several layers of graphitized sp^2^ carbon, and the diameter of the internal cavity is ≈3–5 nm. Due to the large pore volume of carbon nanocages, high loading (77 wt%) of sulfur nanoparticles can be loaded into graphene carbon cage units by solution method to achieve efficient dispersion of sulfur and give full play to their electrochemical activity. The well graphene layer of carbon nanocages is not only conducive to high‐speed electron transport, but also can effectively inhibit the dissolution and shuttle of polysulfide, and improve the cycling performance. The sulfur–carbon composite positive electrode can play the specific capacity of 1375 mAh g^−1^ at 0.1 C current density, and 765 mAh g^−1^ at 5 C; the capacity retention rate is up to 78.4% after 1000 cycles at 1 C. This study proposed graphene‐like carbon nanocages as high‐performance carrier material of sulfur nanoparticles, which opens up a new idea for the rational design of sulfur–carbon composite electrode materials with long‐life and high‐rate performances for the LSBs.

In 2019, Hu and colleagues reported a durable high‐power LSBs cathode with interconnected cubic hollow N‐doped carbon nanocages encapsulated nanoscale sulfur (S@hNCNC) as an active layer (see Figure 22B for details).^[^ ^187^ ^]^ Through electrocatalytic experiments and PDF simulation, the synergistic effect of sulfur adsorption by carbon nanocages and the catalytic conversion of lithium polysulfide by N‐doped sp^2^ carbon was revealed. As a metal‐free catalyst, S@hNCNC has shown excellent performance in high efficiency conversion of sulfur by electrocatalysis. At high current density of 20 A g^−1^, a high capacity of 539 mAh g^−1^ was obtained. The well durability is proved by 1000 cycles at 10 A g^−1^ with a retained capacity of 438 mAh g^−1^. In this study, the functions of “physical limiting,” “chemisorption,” and “catalytic conversion” were skillfully integrated into the N‐doped carbon nanocages, which effectively inhibited the polarization effect and shuttle effect, which provided a guidance for obtaining high‐power and long‐life lithium LSBs electrode materials.

Recently, in 2021, Wang and collaborators from Tsinghua University constructed a multifunctional catalyst of isolated single‐atom nickel (Ni–N_5_/HNPC) using the best Ni–N_5_ active group and N‐doped hollow polyhedral carbon nanocages as carrier material for the sulfur positive electrode (see Figure 22C for details).^[^ ^188^ ^]^ It was found that the carrier improved sulfur conductivity, enhanced the physical–chemical dual limiting ability of lithium polysulfide, and more importantly promoted the kinetics of redox reactions by the active site of Ni–N_5_. Therefore, the prepared Ni–N_5_/HNPC can be used as an ideal sulfur cathode for Li–S batteries, and the Ni–N_5_/HNPC/S cathode has excellent rate performance (average specific capacity of 684 mAh g^−1^ at 4 C), long‐term cycle stability (after 500 cycles, the capacity decay rate per cycle is 0.053%). This work highlights the important role of active site coordination numbers in single atom catalysts and provides a strategy for designing single‐atom active sites functionalized hollow nanostructures for high‐performance LSBs.

A relatively overlooked factor in the loading process of sulfur into hollow carbon is that the low or moderate compatibility (partially wetting) of molten sulfur or sulfur‐dissolved solutions (usually sulfur/CS_2_ solutions) with carbon makes it difficult to fully inject sulfur into hollow carbon by the capillary principle. Moon’ team recently revealed experimentally and theoretically that regulating the interfacial energy on the carbon surface in sulfur solution is the key to promoting the complete coating of sulfur (see Figure 22D for details).^[^ ^189^ ^]^ The HPCSs with hierarchical pore structure (macropore of cavity and mesopores in shell) were used as carbon host. Sulfur solutions were prepared using isopropanol (IPA) or N‐methyl‐2‐pyrrolidone (NMP) or CS_2_. The sulfur/CS_2_ solution results in poor permeability due to its low wettability on carbon surface (sulfur is deposited on the outer surface of HPCS). Solutions containing IPA have low sulfur solution–carbon interfacial energy due to low surface tension, thus improving penetration (sulfur is deposited in the shell of HPCS). The NMP is highly compatible with carbon, therefore, solutions containing NMP show the strongest permeability (sulfur is deposited on the inner surface of HPCS). Note that the sufficient sulfur dispersion in carbon nanocages is the key to design high‐performance LSBs.

Recently, Zhang et al. also successfully synthesized a porous HC nanospheres functionalized with atomic Co as a bifunctional sulfur host, whose atomic Co (including SA Co and Co clusters Co*_n_*) is well supported in the micropores of the HC nanospheres (see Figure 22E for details).^[^ ^190^ ^]^ Using HC nanospheres as an ideal framework, the initial anchoring and subsequent S encapsulation of Co nanoparticles can be achieved. In each HC reactor, it is interesting that the diffusion of sulfur molecules can drag Co (Co*_n_*) atoms to migrate into the carbon shell and form a new Co*_n_*‐HC host. The S@Co*_n_*‐HC showed excellent electrochemical performance in sodium–sulfur battery, suggesting that maximizing the use of atoms optimizes the multiple functions of Co metals in improving sulfur conductivity, activating sulfur reactivity, fixing sulfur, and converting polysulfide. It was confirmed that atomic Co could alleviate the shuttle effect and effectively electrocatalyze the reduction of Na_2_S_4_ to the final product Na_2_S within the HC nanospheres. This work introduces atomic metal into electrode design, which innovatively connects the field of battery and electrocatalyst, and provides a new design direction of electrode material for various sulfur battery technologies.

In order to improve the volumetric properties of the electrode material, the carbon nanocages with ship‐in‐bottle structures can be used to load high‐content sulfur and by improving internal utilization. In 2021, Ma et al. reported the construction of high‐performance sulfur–carbon composite electrodes in carbon nanosheets@porous carbon spheres ship‐in‐bottle construction (see Figure 22F for details).^[^ ^191^ ^]^ First, N‐doped hollow CNCs with customized internal carbon nanosheets were prepared by the restricted pyrolysis of Zn, Co zeolitic imidazolate framework coated resorcinolmelamine‐formaldehyde (ZIF@RMF). The interconnected cavities and porous carbon skeleton ensure good dispersion and immobilization of sulfur cathodes at high sulfur content (80 wt%). In addition, the developed micropores and numerous nitrogen functional groups on the carbon surface are conducive to the chemisorption and transformation of polysulfide and the uniform growth of Li_2_S. The resulting S/CNCs cathode delivers a high initial capacity of 1310 mAh g^−1^@0.2 C, excellent rate performance with 762 mAh g^−1^ at 8 C, as well as ultrahigh stability with a reversible capacity of 841 mAh g^−1^ after 800 cycles. These findings provide an effective method for future development of practical LSBs with superior rate performance and long lifespan. Recently, Xu's group also successfully synthesized a ship‐in‐bottle nanocage structure, namely, yolk–shell structure of mesoporous Mo_2_C@microporous carbon nanocages, as the sulfur host material of Li–S batteries.^[^ ^50^ ^]^ The microporous shell of carbon nanocages inhibited the shuttle of polysulfide and shortened the charge/mass diffusion distance. The Mo_2_C@nanocages yolk–shell hollow structure provides buffer space to accommodate volume changes during the discharge–charge process.

**Figure 23:**
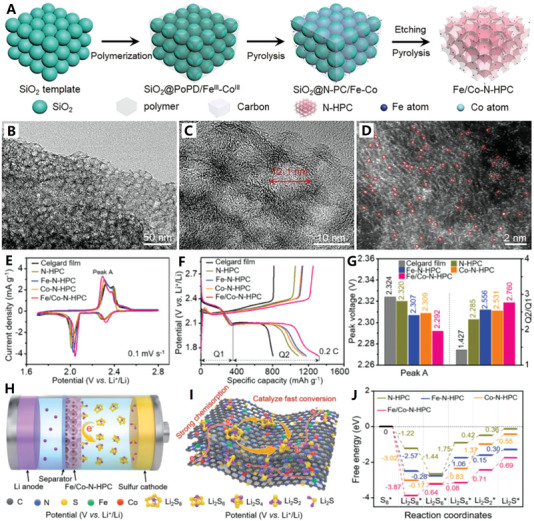
Interconnected and small‐size N‐doped hollow carbon nanocages modified with Fe/Co single atoms for high‐performance Li–S batteries. A) Schematic diagram of synthesis process, B–D) structural characterization, E–G) electrochemical performances, H) device schematic diagram, I) schematic diagram of polysulfide conversion, J) theoretical calculation of free energy. Reproduced with permission.^[^ ^192^ ^]^ Copyright 2022, Wiley.

On the premise of improving the sulfur loading and ensuring good dispersion, designing a dense 3D porous carbon network with interconnected and small‐size carbon nanocages is also a feasible strategy to improve the volumetric performance of sulfur electrodes for Li–S batteries. As recently as 2022 (October), Chen of Beijing Institute of Technology, Xu of Fudan University, Wu of Anhui University of Technology and others constructed an efficient bimetal single atoms‐functionalized carbon nanocages 3D interconnected network electrocatalyst, including N‐coordinated binary metal Fe/Co single atoms (Fe/Co SAs) implanted in N‐doped hierarchical porous carbon (N‐HPC) small nanocage skeleton (see **Figure** **23** for details).^[^ ^192^ ^]^ The efficient conversion of polysulfide was captured and catalyzed by membrane coating strategy on separators. The results show that the introduction of Co atoms can enrich the number of electrons in the active center of Fe, so as to realize the significant synergistic catalysis of the binary metal SAs catalyst and improve the bidirectional catalysis of Li–S redox reactions. Benefiting from the efficient adsorption of dense small carbon nanocages and the cocatalytic effect of binary metal SAs in the process of promoting lithium‐polysulfide conversion, the electrochemical performances of Li–S batteries using the Fe/Co SAs‐N‐HPC modified separator has been greatly improved in both low sulfur loading and high sulfur loading conditions.

The synthesis of Fe/Co SAs‐N‐HPC adopts a typical silicon dioxide template strategy, in which Fe and Co salt in the positive trivalent (Fe^III^–Co^III^) are used as the metallic precursor and poly(o‐phenylenediamine) is used as the carbon source (see Figure 23A). Carbon nanocages have a dense 3D network structure with small pore size (≈12 nm), forming a uniform single atom metal distribution on the carbon substrate (see Figure 23B–D). The Fe and Co bimetallic single‐atom functionalized carbon nanocages (Fe/Co SAs‐N‐HPC) exhibited higher redox current densities and narrower peak distances, with one‐step oxidation and two‐step reduction processes (the minimum peak A potential) (see Figure 23E–G). These results proved that the membrane coating strategy with dense hollow carbon nanocages and bimetallic single atoms can effectively promote the catalytic conversion of lithium polysulfides (Li_2_S_8_, Li_2_S_6_, Li_2_S_4_, Li_2_S_2_, and Li_2_S) (see Figure 23H,I). Theoretical calculations show that the Fe/Co SAs‐N‐HPC has the lowest Gibbs free energy in the continuous conversion, and the rate‐determining step is the reduction process from Li_2_S_4_ to Li_2_S_2_ (only 0.71 eV), suggesting the favorable thermodynamic reduction process (see Figure 23J). The introduction of Co atoms can enrich the number of electrons in the active center of Fe, and the binary metal Fe/Co SAs have obvious synergistic catalytic effect. Therefore, the lithium–sulfur battery using Fe/Co‐N‐HPC modified separator has excellent rate capability and high long‐term cycle stability.^[^ ^192^ ^]^

In addition to Li–S batteries, the hollow carbon nanocages (including nonmetal‐doped and metal‐doped structures) have also been widely applied in sodium–sulfur (Na–S) batteries,^[^ ^190^, ^193^ ^]^ lithium–selenium (Li–Se) batteries,^[^ ^147^ ^]^ sodium–selenium (Na–Se) batteries,^[^ ^126^ ^]^ potassium–selenium (K–Se) batteries,^[^ ^194^ ^]^ which can effectively inhibit the shuttle effect of Li/Na/K polysulfide or Li/Na/K polyselenium. The Se positive electrode has excellent electronic conductivity, which is comparable to the capacity of the S positive electrode, and has attracted extensive attention of researchers. However, due to the shuttle effect of selenide, the reactivity of Se electrode is low, and the capacity decay is fast, which hinders the practical application of Li–Se, Na–Se or K–Se batteries. For example, as efficient polyselenide reservoir the hierarchical multicavity N‐doped hollow carbon nanocages have high adsorption capacity and strong chemical affinity for Se, and the as‐produced anode material demonstrated excellent reversible capacity and long cycle life.^[^ ^195^ ^]^ The unique multicavity structure combined with surface amino group generated a highly conductive, stable 3D carbon network, which provides an effective physical–chemical double barrier effect for Se (namely, efficient anchoring, diffusion, conversion of polyselenide), to obtain rapid oxidation reaction kinetics.

### 5.3 Application Case Analysis of Hollow Carbon Nanocage for Electrocatalysis

At present, functionalized hollow carbon nanocages (such as carbon nanocages with nonmetallic doping, metal SACs and composite interface structures) are widely used in all kinds of electrocatalysis, such as fuel cell electrocatalysis, water splitting electrocatalysis, and other energy conversion electrocatalysis. The typical catalytic reaction involved: ORR, OER, HER, carbon dioxide reduction reaction (CO_2_RR), and other energy‐conversion electrocatalytic reactions. This part focuses on the introduction of single‐atom functionalized hollow carbon nanocages for electrocatalysis.

#### 5.3.1 ORR

ORR is one of the two half reactions necessary in new energy storage and conversion systems such as fuel cells and metal–air batteries. However, the multistep and slow electron transfer and inefficient mass transfer of ORR result in slow dynamics, which greatly hinders their application in practical devices. Therefore, it is necessary to develop efficient ORR catalysts that can achieve rapid charge transfer and mass transfer simultaneously.^[^ ^196^ ^]^ The three‐phase interface of electrocatalyst, electrolyte, and gas at the oxygen electrode will affect the intrinsic activity of the catalysts. The three‐phase interfacial microenvironment controls the diffusion of substances (reaction gases, water, and hydroxide ions) and the transfer of electrons. Therefore, reasonable catalyst design (such as hydrophilic hollow porous carbon nanocages) can adjust the three‐phase interface microenvironment, thus improving the catalytic performance of the catalyst and promoting the electrode reaction process.^[^ ^197^ ^]^ For example, the three‐stage porous N‐doped hollow carbon nanocages with micropore, mesopore, and macropore exhibited the best electrocatalytic performance of ORR: in this new three‐stage porous hollow system, macropores can store reactants, mesoporous can improve the efficiency of reactants transport, and micropores are conducive to the accumulation of reactive ions.^[^ ^198^ ^]^ These hydrophilic and hierarchical hollow porous carbon nanocages can be used as qualified carrier materials for designing high‐performance metal single‐atom ORR catalysts (including precious metals (Pt) and transition metals (Fe, Co, and Ni) SACs).

**Figure 24:**
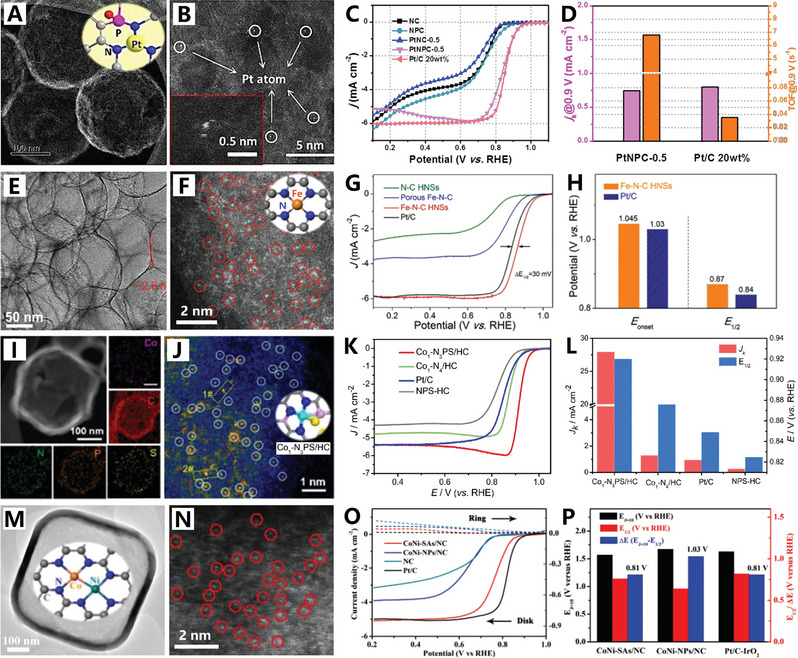
Hollow carbon nanocages as efficient ORR electrocatalysts. A–D) Carbon nanocages‐supported single‐atom Pt catalyst. Reproduced with permission.^[^ ^199^ ^]^ Copyright 2021, Wiley. E–H) carbon nanocages‐supported single‐atom Fe catalyst. Reproduced with permission.^[^ ^145^ ^]^ Copyright 2019, Wiley. I–L) Carbon nanocages‐supported single‐atom Co catalyst. Reproduced with permission.^[^ ^135^ ^]^ Copyright 2020, Wiley. M–P) Carbon nanocages‐supported dual‐atom Co,Ni catalyst. Reproduced with permission.^[^ ^200^ ^]^ Copyright 2019, Wiley.

Platinum (Pt) is the most efficient ORR electrocatalyst at present. It is the most important task to provide the utilization of Pt atoms and reduce the amount of Pt metal. The implementation of carbon‐supported single‐atom Pt catalysts is expected to minimize the content of Pt, and hollow carbon materials can promote oxygen diffusion and promote the accessibility of Pt sites to further improve the ORR performance. Recently, Amal and collaborators realized a Pt monatomic oxygen reduction catalyst with high activity, high stability, high conversion efficiency (or turnover frequency (TOF)), and only 0.026 wt% content (hollow carbon sphere‐supported single atom Pt catalyst) (see **Figure** **24**A–D for details).^[^ ^199^ ^]^ The DFT simulation results show that when P atoms replace the *α*‐carbon at Pt–N_3_, the d band center of Pt becomes deeper, which is conducive to oxygen reduction. The catalyst was tested to contain only ≈0.026 wt% Pt, but showed comparable oxygen reduction activity to commercial Pt/C (20 wt%) (with high half‐wave potential close to 0.85 V) in 0.1 m KOH. The TOF of single atom Pt was as high as 6.80 s^−1^ (at 0.9 V), nearly 170 times that of commercial Pt/C. It also has high stability and excellent TOF over 15 000 cycles of testing. This work opens up a new strategy for designing ultralow platinum loading and high‐performance ORR electrocatalysts.

The Fe SACs based on the famous Fe–N–C (Fe–N_4_) structure has attracted extensive research because of its maximum atomic utilization and excellent ORR characteristics (comparable to Pt). Previously, Tang and co‐workers used the inexpensive biomaterial His as an N and C source to elaborate a SiO_2_ templated synthesis of immobilized and dispersed Fe single atoms on hollow N‐doped carbon spheres (Fe–N–C HNSs) (see Figure 24E–H for details).^[^ ^145^ ^]^ With numerous atomically dispersed Fe–N_4_ and unique spherical hollow structure, the prepared Fe–N–C HNSs showed excellent ORR performance in alkaline KOH medium, with high activity, long‐term stability, and excellent tolerance to methanol, exceeding commercial Pt/C catalysts and most previously reported non‐noble metal catalysts. The *E*_onset_ and *E*_1/2_ of Fe–N–C HNS were 1.046 and 0.87 V, respectively, which exceeded the Pt/C values (1.03 and 0.84 V). Even after 10 000 consecutive cycles, the *E*_1/2_ of Fe–N–C HNS did not decay significantly. The excellent ORR performance of the prepared Fe–N–C HNS is attributed to the unique structure and chemical composition. 1) Hollow carbon nanospheres with thin walls and open frames can effectively expose and utilize the active sites. 2) Mesoporous pores in carbon nanospheres can significantly expand the electrolyte/catalyst contact area and promote O_2_ diffusion. 3) High graphitization of Fe–N–C HNSs can enhance electrical conductivity and promote electron transfer. 4) The high content of pyridine‐N and graphite‐N not only facilitates the adsorption of O_2_, but also promotes the conductivity and hydrophilicity.

In 2020, Chen et al. demonstrated a correlation between atomic configuration‐induced electron density at the active site of single‐atom Co catalyst and ORR performance using a combination of DFT calculations and electrochemical analysis (see Figure 24I–L for details).^[^ ^135^ ^]^ Under the guidance of DFT calculation, an MOFs‐derived single‐atom Co catalyst was designed and synthesized. The best active part of Co_1_‐N_3_PS was added to the hollow carbon polyhedron (Co_1_‐N_3_PS/HC). As predicted, Co_1_‐N_3_PS/HC exhibits excellent basic ORR activity with a half‐wave potential of 0.920 V, good ORR kinetics, a record current density, and an ultralow Taffel slope of 31 mV dec^−1^, which exceeds Pt/C and almost all nonprecious metal ORR electrocatalysts. The ORR kinetics of Co_1_‐N_3_PS/HC is better than that of Pt/C even in more acidic medium. This paper confirms the key role of optimal electron density in the active center of atomically dispersed Co caused by the cooperative coordination of unique N, P, and S atoms in the dramatic improvement of ORR performance. This work provided a new and universal method for the rational design of high‐performance polyhedral carbon nanocages functionalized with single atomic catalysts.

In 2019, Han et al. also proposed a controlled pyrolysis strategy of dopamine (DPA)‐coated MOF to synthesize a new type of atomically dispersed Co–Ni bimetallic site material supported by N‐doped hollow carbon nanocages (CoNi‐SA/NC) for ORR (see Figure 24M–P for details).^[^ ^200^ ^]^ The bimetallic Co–Ni sites with high reactivity can reduce the potential barrier, accelerate the reaction kinetics, and greatly contribute to the improvement of ORR activity. The CoNi‐SA/NC in 0.1 m KOH had a H_2_O_2_ content of less than 6% and a *n*‐value of more than 3.9, indicating high ORR selectivity and efficient 4e^−^ transfer mechanism. The overall reversible electrocatalytic redox properties of the catalysts were analyzed by oxygen electrode activity (Δ*E*) (difference between OER potential at 10 mA cm^−2^ and ORR half‐wave potential). Surprisingly, similar to Pt/C‐IrO_2_, the CoNi‐SA/NC has a Δ*E* value of 0.81 V, which exceeds most of the reported non‐noble metal efficient bifunctional catalysts. Such adjacent double active center of M_1_M_2_–N–C in carbon nanocages not only activates the adsorbed O_2_ molecule, but also changes the adsorption state of each intermediate in the process of ORR, thus effectively reducing the reaction energy barrier and improving the catalytic activity.^[^ ^149^ ^]^

On the other hand, carbon nanocages enveloped metal nanoparticles also showed excellent ORR catalytic performances. For example, Hu and co‐workers reported that the Pt nanoparticles encapsulated inside N‐doped carbon nanocages (Pt@NCNC) presented excellent alcohol‐tolerant ORR activity and durability in acidic media, far superior to the Pt counterpart immobilized outside the nanocages (Pt/NCNC).^[^ ^24^ ^]^ Ying et al. demonstrated that the MOFs derived Pt–Co bimetallic nanoparticles within N‐doped hollow porous carbon capsules can be used as a highly active and durable catalyst for ORR, with a mass activity that is 5.5 times greater than that of commercial Pt/C.^[^ ^31^ ^]^ The 3D Co (nanoparticles)‐N‐doped hollow carbon spheres are proved to be excellent bifunctional electrocatalysts for ORR and OER, thanks to the high surface area and 3D hollow architecture.^[^ ^33^ ^]^ The MOFs derived Co nanoparticles‐functionalized hybrid double‐shelled carbon nanocages are also a high‐performance bifunctional electrocatalyst for ORR and OER, due to the high activity of Co–N–graphene shells into the robust N‐doped carbon hollow framework.^[^ ^75^ ^]^

#### 5.3.2 OER

Electrochemical decomposition of water to produce green hydrogen is expected to solve the problems of energy shortage and environmental pollution, but the overall conversion efficiency is limited by OER due to its slow reaction kinetics.^[^ ^201^ ^]^ Therefore, an efficient electrocatalyst is needed to reduce the OER overpotential and improve the efficiency of water splitting. In addition, the OER is also an essential electrode reaction process for metal–air batteries, such as zinc–air batteries. Metal nanoparticles (or metal single atom species) encapsulated in carbon layers play an important role in the formation of active centers in electrocatalysts.^[^ ^202^ ^]^ The unique hollow and porous structure of carbon nanocages can effectively increase the specific surface area of the material and promotes the exchange of electrons and substances, which is very beneficial to improve the OER performances, such as high catalytic kinetics.^[^ ^203^ ^]^

**Figure 25:**
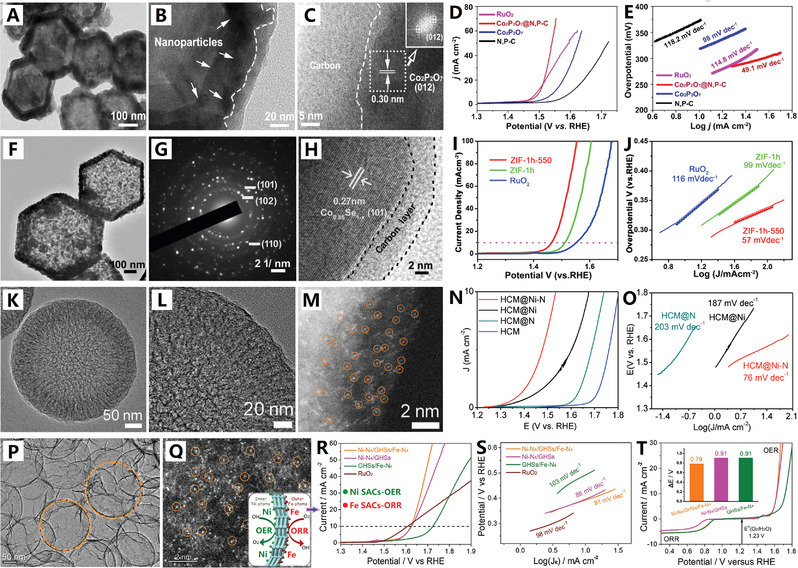
Hollow carbon nanocages as efficient OER electrocatalysts. A–E) N,P codoped polyhedral carbon nanocages‐supported Co_2_P_2_O_7_ nanoparticles. Reproduced with permission.^[^ ^204^ ^]^ Copyright 2020, Elsevier. F–J) Polyhedral carbon nanocages‐supported Co_0.85_Se_1‐_ *_x_* nanoparticles. Reproduced with permission.^[^ ^205^ ^]^ Copyright 2021, Wiley. K–O) Mesoporous carbon nanocages‐supported single‐atom Ni catalyst. Reproduced with permission.^[^ ^206^ ^]^ Copyright 2019, Wiley. P–T) Graphene hollow spheres (GHSs)‐supported single‐atom Ni and Fe Janus catalyst. Reproduced with permission.^[^ ^170^ ^]^ Copyright 2020, Wiley.

In the development of carbon nanocages‐based OER electrocatalysts, the regulation of valence electron states near the Fermi level in the metal center is a key element to obtain efficient oxygen evolution kinetics. In 2020, Liang et al. reported a novel Mott–Schottky heterojunction electrocatalyst, Co_2_P_2_O_7_@N,P codoped polyhedral carbon nanocages for OER (see **Figure** **25**A–E for details).^[^ ^204^ ^]^ The N,P codoped carbon layer as cocatalyst can effectively regulate the excess Co center occupation and stabilize the microstructure of Co_2_P_2_O_7_ nanoparticles. The DFT calculations showed that the built‐in electric field promoted the local charge polarization of the heterogeneous interface and greatly promoted the adsorption of the intermediate (OOH*) with high intrinsic activity. The prepared Co_2_P_2_O_7_@carbon nanocages electrocatalyst has excellent OER catalytic kinetics performance (with a Tafel slope of 49.1 mV dac^−1^), and the overpotential is only 310 mV at a current density of 50 mA cm^−2^ and ultrahigh electrochemical stability (more than 100 h). This work demonstrates the concept of charge polarization of a hollow carbon nanocage‐based heterogeneous interface to accelerate the water splitting OER over highly efficient nanoscale electrocatalysts.

In 2021, Lin et al. designed a general selenic acid‐assisted etching strategy using a metal–organic framework as the precursor to implement polyhedral carbon nanocages encapsulated 3D metal selenide M_m_Se_n_ (Co_0.85_Se_1‐_ *_x_*) with selenium‐rich vacancies as a high‐performance noble metal‐free OER electrocatalyst (see Figure 25F–J for details).^[^ ^205^ ^]^ The ZIF‐67 framework was partially etched by selenic acid under moderate conditions, and was also used as a source of selenium. Finally, after calcination in an inert atmosphere at moderate temperature, carbon‐coated M_m_Se_n_ derivatives were obtained. Importantly, compared with the traditional calcination method, selenic acid‐assisted etching strategy has succeeded in introducing rich selenium vacancy in M_m_Se_n_/C hollow structure, which greatly increased the active site and improve electrical conductivity (significantly improve the catalytic activity of OER with a Tafel slope of 57 mV dac^−1^). At the OER current density of 10 mA cm^−2^, the Co_0.85_Se_1‐_ *_x_* @C nanocages only provide 231 mV overpotential, while the corresponding all‐water electrolyzer only needs 1.49 V at the current density of 10 mA cm^−2^ in alkaline medium. In conclusion, this work provides a new opportunity to realize high‐performance carbon nanocages‐based OER electrocatalysts using a general strategy of selenic acid etching vacancy engineering.

Previously, Prof. Lou's team from Nanyang Technological University in Singapore reported that the atomic‐scale Ni site distributed on N‐doped hollow carbon nanocages (hollow mesoporous carbon spheres) matrix (HCM@Ni–N) can be used as an efficient OER electrocatalyst under alkaline conditions (see Figure 25K–O for details).^[^ ^206^ ^]^ It was found that the catalytic activity was significantly enhanced in the optimized Ni active site coordination geometry (the overpotential is 300 mV at a current density of 10 mA cm^−2^ and the Tafel slope is 76 mV dac^−1^). In addition, the use of Ni–N coordination will carry out effective electron coupling and reduce the Fermi level and the adsorption energy of the intermediate, thus improving the reaction kinetics of OER. The N doping also greatly changes the 3d orbital distribution of Ni in HCM@Ni–N and significantly improves the charge polarization of Ni, thus greatly reducing the reaction barrier of the OER reaction. Overall, this work reveals a correlation between catalytic activity and the electronic structure of the isolated active site on carbon nanocages, thus shedding new light on the origin of the high activity of SACs.

On the premise of designing SACs with high activity and selectivity, it is very important to construct efficient electrocatalysts with independent ORR/OER dual functional interfaces to accelerate the development of rechargeable metal–air batteries. In order to realize the construction of bimetallic SACs with independent functional interfaces, Chen et al. chose SiO_2_ as hard template, and formed a Ni precursors–graphene–Fe precursor three‐layer interface by layering self‐assembling route, and the Ni–N_4_/GHSs/Fe–N_4_ ORR/OER bifunctional catalyst was obtained in the subsequent pyrolysis process (see Figures 13D and 25P–T for details).^[^ ^170^ ^]^ Within the GHSs, the inner layer was single atom Ni (OER active component) and the outer layer was single atom Fe (ORR active component) (Janus bimetallic SACs) (see Figure 25P,Q). The electrochemical test showed that Ni–N_4_/GHSs/Fe–N_4_ had high activity on ORR and OER. The half‐wave potential of ORR was 0.83 V, while the overpotential of OER *E*_j10_ was only 0.39 V (Tafel slope was 81 mV dac^−1^ and the Δ*E* = 0.79 V) (see Figure 25R,T). The dual‐functional Ni–N_4_/GHSs/Fe–N_4_ air cathode showed a large specific capacity (777.6 mAh g_Zn_^−1^, 94.8% of the theoretical value) and energy density (970.4 Wh kg_Zn_^−1^, 89.5% of the theoretical value). These results provide an idea for the construction of bimetallic SACs with independent functional interfaces on the two sides of hollow carbon nanocages.

**Figure 26:**
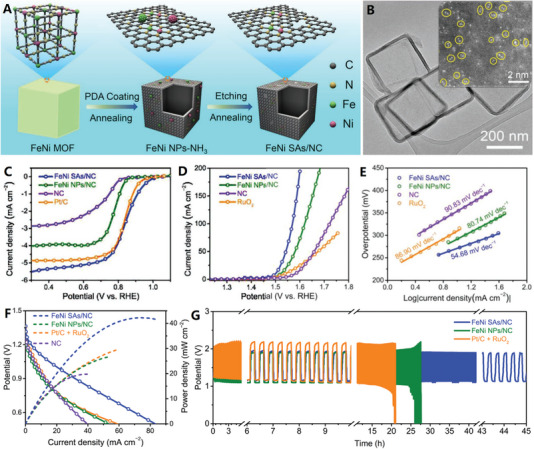
Fe–Ni dual atoms embedded in a N‐doped carbon (cubic carbon nanocages) (FeNi SAs/NC) for the bifunctional ORR/OER electrocatalysts and high‐performance zinc–air batteries. A) Schematic diagram of synthesis process, B) structural characterization, C–E) electrochemical performances, and F,G) device performances. Reproduced with permission.^[^ ^207^ ^]^ Copyright 2021, Wiley.

Dual‐site single‐atom catalysts (or diatomic catalysts) are expected to achieve effective regulation of multiple reaction processes (or synergistic catalytic effects of two sites).^[^ ^144^ ^]^ Recently, Yu et al. reported that atomically dispersed Fe–Ni dual atoms embedded in a N‐doped carbon matrix (cubic carbon nanocages) (FeNi SAs/NC) were successfully developed with remarkable electrocatalytic activity in ORR and OER (see **Figure** **26** for details).^[^ ^207^ ^]^ The FeNi NPs‐NH_3_ was obtained from FeNi‐MOF/DPA by calcination at 500 °C for 2 h under an ammonia stream. Then, FeNi NPs‐NH_3_ was immersed in nitric acid solution (5 m) for acid treatment, the solution was heated to 80 °C, stirred for 1 h, washed and dried, annealed at 900 °C, forming FeNi SAs/NC hollow cubes (see Figure 26A,B). The diatomic FeNi SAs/NC cubic carbon nanocages showed high initial potential (0.98 V) and half‐wave potential (0.84 V) for ORR and low overpotential (270 mV) for OER at 10 mA cm^−2^ (Tafel slope was 54.68 mV dac^−1^ and the Δ*E* = 0.66 V) (see Figure 26C–E). DFT calculation shows that Fe site, as the active center, can promote the four‐electron reaction process, while Ni site regulates the electronic structure of Fe site, which further reduces the energy barrier of the speed control step. In addition, the N‐doped carbon matrix can prevent the aggregation and corrosion of metal atoms, thus improving the durability of the catalyst. As a proof of concept, flexible quasi‐solid zinc–air battery assembled with FeNiSAs/NC catalysts exhibited promising battery properties, including: lower overpotential between charge and discharge, excellent peak power density, and higher discharge specific capacity and good cycle stability (better than noble metal‐based (Pt/C and RuO_2_) catalysts) (see Figure 26F,G). This work provides a reasonable guidance for the synthesis of carbon nanocages‐based bifunctional electrocatalysts in the next generation energy devices of flexible electronics.

#### 5.3.3 HER

In the process of water splitting, a suitable catalyst is often needed to promote the HER at the cathode. To date, Pt is considered to be the metal with the highest catalytic activity against HER in the periodic table. However, due to the scarce reserves and high price of Pt, its wide application is greatly limited. To overcome this shortcoming, it is necessary to find an inexpensive alternative to Pt catalyst or reduce the metal loading of Pt to improve the catalytic performance of HER electrocatalyst.^[^ ^208^ ^]^ The high‐performance confined Pt‐based or non‐Pt‐based HER catalysts can be easily constructed by using the controllable structure and composition of carbon nanocages (for example, the synergistic effect of trapping action of cage wall micropores and pyridine nitrogen anchoring along edges of micropores). These carbon nanocages‐supported metal catalysts often exhibit very low hydrogen evolution overpotential, high mass activity, and excellent stability. The synergistic effect of micropore trapping and heteroatom anchoring of carbon nanocages provides a new idea and method for the construction of highly stable single atom catalysts, which is of great value for promoting the basic research and practical application of single atom catalysts.^[^ ^209^ ^]^

**Figure 27:**
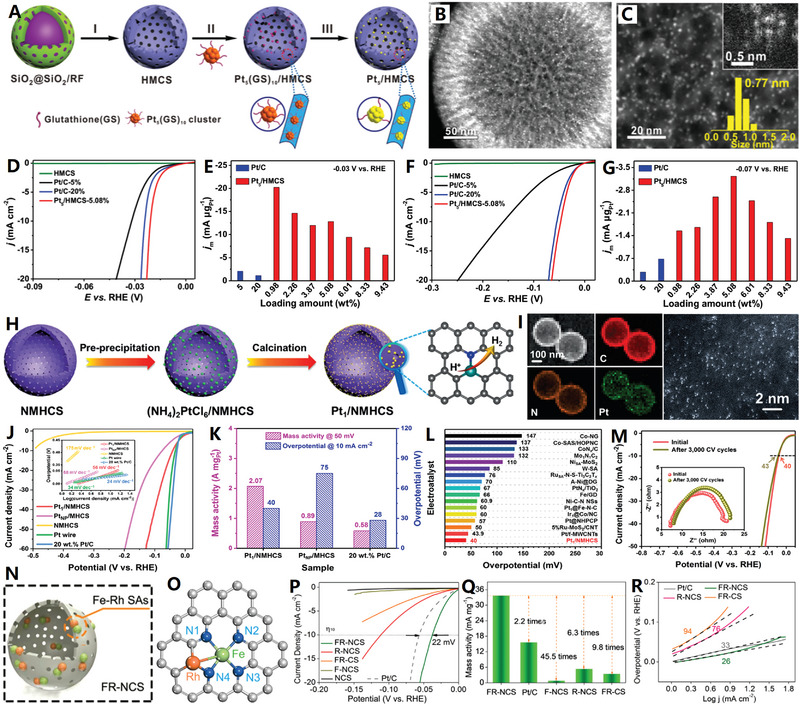
Hollow carbon nanocages as efficient HER electrocatalysts. A–G) Hollow mesoporous carbon spheres‐supported Pt clusters (Pt_5_/HMCS). Reproduced with permission.^[^ ^112^ ^]^ Copyright 2020, Wiley. H–M) N‐doped mesoporous hollow carbon spheres‐supported Pt single atoms (Pt_1_/NMHCS). Reproduced with permission.^[^ ^210^ ^]^ Copyright 2021, Wiley. N–R) N‐doped hollow carbon nanocages‐supported dual single‐atom Rh–Fe catalyst. Reproduced with permission.^[^ ^211^ ^]^ Copyright 2020, Wiley.

Because of the high atomic ratio on the surface and the adjustable composition and electronic structure, metal clusters can be potential catalysts for HER. Recently, Lou and co‐workers reported the synthesis of Pt clusters with domain limited structure using hollow mesoporous carbon spheres as carrier material (Pt_5_/HMCS) for efficient HER (see **Figure** **27**A–G for details).^[^ ^112^ ^]^ This domain limited structure can effectively stabilize the Pt clusters during ligand removal and show excellent hydrogen evolution performance under acidic and alkaline conditions. The Pt_5_/HMCS catalyst showed higher HER catalytic activity with the overpotential of 20.7 mV (acidic) (Figure 27D,E) and 46.2 mV (alkaline) (Figure 27F,G) at 10 mA cm^−2^. The mass activity of the optimized Pt_5_/HMCS catalyst is 12 times that of the commercial Pt/C catalyst with the same Pt loading (≈5 wt%) in alkaline condition. After 3000 CV tests, the commercial Pt/C have obvious performance attenuation, while the Pt_5_/HMCS’ performance attenuation is very weak, and the test in alkaline environment almost no attenuation, showing the stability of Pt clusters has been greatly improved by the hollow mesoporous carbon spheres. This study provides a simple and effective method for the design and modulation of noble metal clusters catalyst supported by hollow mesoporous carbon nanocages.

EMSI can induce the electron transfer between the metal and the support, regulate the electronic state of metal, and optimize the reduction of intermediate species, which plays a crucial role in HER catalytic reaction. In 2021, Yu's research group reported the use of strong EMSI engineering to control the electronic structure of N‐doped mesoporous hollow carbon spheres loaded with Pt single atoms (Pt_1_/NMHCS) for efficient HER (see Figure 27H–M for details).^[^ ^210^ ^]^ By synthesizing Pt_1_/NMHCS with strong EMSI effect, the stability of Pt single atoms is the result of the natural coupling of 3D hollow structure and N doping. For electrocatalyzing HER, the Pt_1_/NMHCS shows not only higher hydrogen evolution activity than the reference catalyst (i.e., Pt_NP_/NMHCS and 20 wt% Pt/C), but also excellent durability due to the minimal aggregation of Pt atoms in the long‐time test (3000 CV). The mass activity of Pt_1_/NMHCs, Pt_NP_/MHCs, and 20 wt% Pt/C is 2.07 A, 0.89 A, and 0.58 A mg^−1^ Pt, respectively, at the overpotential of 50 mV, which clearly verifies that the utilization efficiency of Pt atom in Pt_1_/NMHCs is significantly improved. The Tafel slope of Pt_1_/NMHCs is only 56 mV dec^−1^, suggesting a Volmer–Heyrovsky mechanism in HER process. The overpotential is only 40 mV at 10 mA cm^−2^. Theoretical calculation proves that the strong EMSI effect in the unique coordination structure of “N_1_–Pt_1_–C_2_” allows fine modulation of the 5d state of Pt_1_ single atoms, which optimizes the reduction of intermediate species and promotes the generation of H_2_, thus giving the Pt_1_/NMHCs excellent catalytic activity against HER.

Atomic‐dispersed metal catalysts supported by hollow carbon nanocages have high reactivity and selectivity by utilizing metal components and relatively stable ligand structure to the maximum extent. However, due to the drive of thermodynamic stability, forming atomic‐scale multicomponent metals without aggregation remains a great challenge. Zhou et al. recently proposed a top‐down process that spontaneously converts Fe nanoparticles to Fe single atoms at low temperatures using N‐doped hollow carbon nanocages as carriers, starting with Fe nanoparticles and using intermetallic bonding (Rh—Fe bonding) as a chemical promoter for efficient HER (see Figure 27N–R for details).^[^ ^211^ ^]^ The presence of Rh—Fe bonds between adjacent Fe and Rh atoms contributes to thermodynamic stability and promotes the stripping of individual Fe atoms from Fe nanoparticles, resulting in stable diatomic (C_2_N_1_Rh‐FeN_4_) construction. The dual single‐atom Rh—Fe catalyst has good electrocatalytic performance for the HER in acidic electrolyte. Due to the stable Rh and Fe single atom centers, the catalyst exhibited very high HER activity, with an overpotential of 36 mV at 10 mA cm^−2^ and a Tafel slope of 26 mV dec^−1^, as well as ultrahigh mass activity and excellent cyclic stability, greatly exceeding the commercial Pt/C catalyst. The discovery of bimetallic bond cooperation as chemical accelerators opens up a new way for the atomic dispersion of multicomponent metals and the design of efficient catalysts on the atomic scale with the hollow carbon nanocages as carriers.

**Figure 28:**
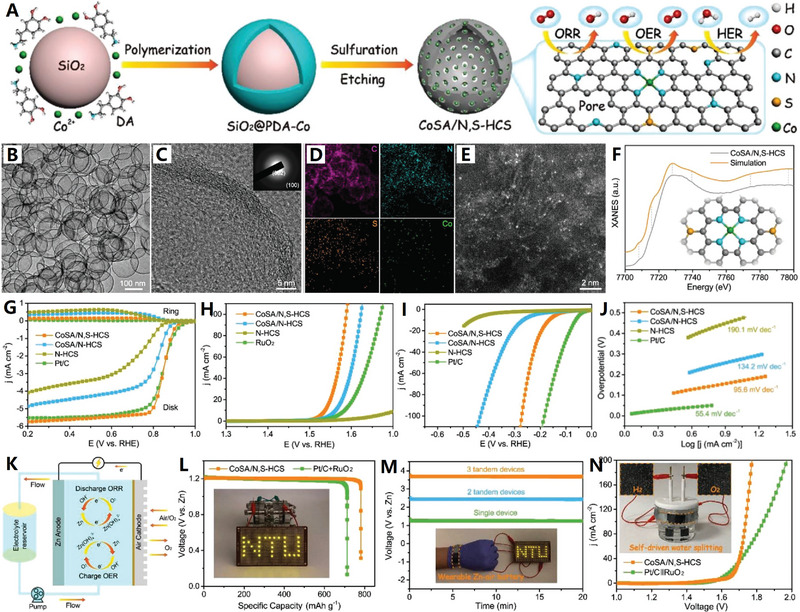
Single atom Co on hollow carbon spheres codoped with N and S (CoSA/N,S‐HCS) for the trifunctional ORR/OER/HER electrocatalysts. A) Schematic diagram of synthesis process, B–F) structural characterization, G–J) electrochemical performances, K) liquid zinc–air batteries, L,M) flexible zinc–air batteries, and N) all‐water decomposition system. Reproduced with permission.^[^ ^143^ ^]^ Copyright 2020, Wiley.

In renewable energy technologies, the development of efficient and inexpensive nonprecious metal alternatives as trifunctional active electrocatalysts in multiple electrochemical reactions (such as HER, OER, and ORR) is a top priority.^[^ ^212^ ^]^ In 2020, Prof. Chen's research group anchored single atom Co on hollow carbon spheres codoped with N and S (denoted as CoSA/N,S‐HCS) to form an efficient trifunctional SACs for efficient HER, OER, and ORR (see **Figure** **28** for details).^[^ ^143^ ^]^ The electron regulation of the active center of Co is realized by the short‐range N coordination and the long‐range S electron supply. By introducing secondary atom doping with relatively weak electronegativity (i.e., S) to optimize the local coordination environment of the active metal center, the catalytic activity of the Co–N_4_ single atom can be further improved. The CoSA/N,S‐HCS showed excellent three‐function electrocatalytic activity and stability for ORR, OER, and HER, and the performance was much better than that of CoSA/N‐HCS catalysts without doping S atoms. In‐depth experimental characterization and theoretical mechanism studies reveal synergies between atomically dispersed Co–N_4_ active centers, adjacent electron donor S doping, and unique hollow carbon nanocages carriers. In addition, when CoSA/N,S‐HCS were assembled into liquid or flexible solid‐state rechargeable zinc–air batteries, the devices demonstrated high power density and specific capacity, and showed good long‐term cycle stability, superior to battery systems based on commercial Pt/C + RuO_2_ dual catalysts. Finally, using CoSA/N,S‐HCS as the sole catalyst, we demonstrate an all‐water decomposition system powered by a flexible zinc–air battery with hydrogen production rates up to 184 µmol h^−1^. This work provides a new idea for the design and working mechanism of trifunctional ORR/OER/HER SACs supported on the hollow carbon nanocages with multiple heteroatomic doping structures.

#### 5.3.4 CO_2_RR

At present, the excessive emission of greenhouse gas of CO_2_ has caused increasingly serious climate problems. The preparation of high value‐added chemical fuel by CO_2_RR is an effective way to reduce CO_2_ emission. Therefore, the design of CO_2_RR catalysts with high selectivity and activity has become a research hotspot in recent years. Among them, the nickel–nitrogen–carbon (Ni–N*_x_*–C) catalyst showed high Faraday efficiency and conversion frequency to the product CO, and was considered as a potential CO_2_RR electrocatalyst.^[^ ^213^ ^]^ However, the Ni–N*_x_*–C catalyst still has some problems such as insufficient activity, low activity density, and slow charge transfer kinetics, so by choosing a new carbon carrier has a unique shape or structure (such as hollow porous carbon nano cage) and/or heteroatomic doping (N, S, and F, etc.) and to regulate activity structure, is an effective way to solve these problems.^[^ ^214^ ^]^ In order to regulate the active density and overall property of Ni–N*_x_*–C catalysts, constructing carbon nanocages with controllable thickness and pore size or designing multicavity carbon nanocages is an effective alternative strategy.

**Figure 29:**
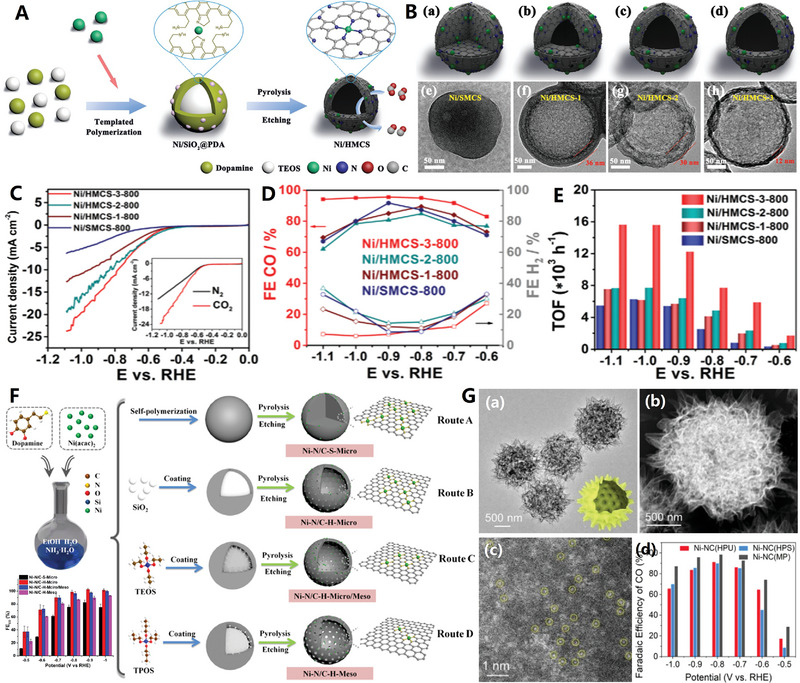
Hollow carbon nanocages as efficient CO_2_RR electrocatalysts. A–E) hollow mesoporous carbon spheres‐supported Ni–N_4_ single atoms. Reproduced with permission.^[^ ^215^ ^]^ Copyright 2020, Wiley. F) Micro/mesoporous hollow carbon spheres‐supported Ni–N_4_ single atoms. Reproduced with permission.^[^ ^216^ ^]^ Copyright 2022, Elsevier. G) Multicavity hollow porous urchin‐like N‐doped carbon nanocages‐supported Ni–N_4_ single atoms. Reproduced with permission.^[^ ^133^ ^]^ Copyright 2022, Wiley.

SACs have become a research hotspot due to their high atomic utilization and excellent activity. However, the role of the carrier structure in SACs is usually neglected in the catalytic process. In 2020, Cao's team successfully synthesized a series of Ni–N_4_ SACs supported by carbon spheres with different structures (including solid mesoporous carbon spheres and hollow mesoporous carbon spheres with different wall thickness) by fine‐adjusting the synthesis conditions (see **Figure** **29**A–E for details).^[^ ^215^ ^]^ The Ni–N_4_ catalyst (Ni/HMCS‐3 800) supported by hollow mesoporous carbon spheres with thin‐walled structure (12 nm) showed excellent catalytic activity for the electrocatalytic CO_2_RR. In the potential range of −0.7 to −1.1 V (vs RHE), Faraday efficiency for CO is up to 95%, with conversion frequency values up to 15 608 h^−1^. The effect of the geometric structure of carbon carrier on the performance of CO_2_RR was further studied. It was proved that the shell thickness and density of carbon can effectively regulate the chemical environment of N‐doped species in carbon skeleton, and promote the activation of CO_2_ molecules. In addition, the optimized mesoporous size is conducive to improving the mass diffusion and overflow within the hollow carbon materials, thus greatly increasing the adsorption capacity of CO_2_. This work provides a new direction for improving the CO_2_RR catalytic performance of SACs on carbon nanocages.

In the recent 2022, Cao et al. designed and synthesized four kinds of Ni single atom electrocatalysts with different morphologies and pore structures of N‐doped carbon spheres (including solid and hollow carbon spheres) by using the improved Stober method, and discussed the influence of catalyst morphologies and pore structures on the formation of single atom Ni active sites and CO_2_RR catalytic performances (see Figure 29F for details).^[^ ^216^ ^]^ The four synthesized catalysts are microporous solid carbon spheres (Ni–N/C–S‐Micro), microporous hollow carbon spheres (Ni–N/C–H–Micro), micro/mesoporous hollow carbon spheres (Ni–N/C–H–Micro/Meso), and mesoporous hollow carbon spheres (Ni–N/C–H–Meso). The test results show that Ni–N/C–H–Micro/Meso has a CO Faraday efficiency of more than 90% (−0.7 to −1.0 V vs RHE), CO current density of 16.2 mA cm^−2^, and the selectivity to CO remains above 90% after the stability test for 5 h. These results indicate that the micro/mesoporous structure of the catalyst contributes to the mass transfer and the exposure of the active site in the reaction, which enhances the catalytic activity. Compared with the microporous structure, the mesoporous structure shows better catalytic activity and reaction kinetics. In short, the hollow mesoporous carbon nanocages can be used in electrocatalytic CO_2_RR field as a carrier because of their advantages such as high surface area, adjustable pore structure, good conductivity, and excellent chemical stability, which contribute to the dispersion and mass transfer of single atoms.

Among the many structures that support single metal sites, hollow structures with double‐sided usable surfaces and abundant voids are one of the most effective structures for designing efficient SACs.^[^ ^170^ ^]^ However, the preparation of most hollow Ni–N*_x_*–C materials requires high‐temperature pyrolysis and solid templates without catalytic properties. Therefore, exploring new strategies for SACs hollow structures with special morphology, high conductivity, and high content accessible to single metal sites is particularly important, but a huge challenge. In view of this, in 2022, Prof. Lou from Nanyang Technological University developed a low‐temperature Ni catalytic templating strategy to anchor Ni single atoms in multicavity hollow porous urchin‐like N‐doped carbon nanocages (Ni‐NC (HPU)) with high crystallinity and high Ni content (2.4 wt%), showing excellent activity and stability in CO_2_RR (see Figure 29G for details).^[^ ^133^ ^]^ The as‐prepared Ni‐NC(HPU) with high crystallinity exhibits unique structures, allowing full exposure of monodisperse Ni sites and efficient electron/mass transfer. When used to catalyze CO_2_RR, the Ni‐NC(HPU) catalyst has high CO selectivity, significant CO partial current density and good stability. This work has important implications for the design and manufacture of advanced multicavity hollow porous nanostructured SACs for a variety of energy storage and conversion applications.

## 6 Opportunities and Challenges

In this review, the applications of carbon nanocages in many electrochemical fields, including electrochemical energy storage and electrochemical catalytic conversion, are summarized, and discussed in detail. Our main objective is to comprehensively analyze the application principles and performance optimization of functional carbon nanocages (such as doped carbon nanocages or composite carbon nanocages) in electrochemical energy storage (mainly supercapacitors and secondary batteries). Looking at the whole overview, we can find that, having a high specific surface area (e.g., >800 cm^2^) g^−1^) and reasonable pore structures (such as abundant micropores (<2 nm) or small mesoporous (2–10 nm) structures on carbon shells) of amorphous carbon nanocages (such as microporous hollow carbon cubes, mesoporous carbon nanospheres, etc.) are very favorable for the formation of electric double layer capacitance (of course, they can also be used as the basis for further construction of pseudcapacitor materials). These porous amorphous carbon nanocages also play a positive role in secondary battery energy storage (such as lithium storage or sodium storage). However, for battery applications, graphitized carbon nanocages are the real desirable and desirable anode materials (because graphite is a commercial anode material with a high reversible capacity and a low voltage platform). In addition, for lithium–sulfur batteries, carbon nanocages are a unique carrier material, whose unique structure of nanocages has a synergistic effect on promoting electrochemical performances, including physical confinement effect, chemical adsorption of functional groups and catalytic conversion of single atom metals on nanocages.

On the other hand, in this paper, we also systematically summarized the application of carbon nanocages in electrocatalytic energy conversion (including fuel cells, metal–air cells, water electrolysis to produce hydrogen, electrocatalytic carbon dioxide conversion, etc.). There is no doubt that in electrocatalytic systems, the core components of metallic catalysts, carbon materials are developed as conductive carriers or carriers for stabilizing metal components. However, the nanostructure of carbon support (including pore structure) plays a very important role in metal catalysts, especially for single‐atom metal catalysts (such as Fe–N–C), where carbon itself is a part of the catalyst. The carbon atom vacancy (multivacancy) on the wall of carbon nanocages is the best position for anchoring metal atoms, while the nitrogen atom on carbon is the first choice for metal coordination. The porous and hollow structures of carbon nanocages provide good conditions for mass transfer and gas diffusion of electrocatalysts. Therefore, in the field of electrochemistry, carbon nanocages‐based electrocatalysts are a promising and challenging research direction. In summary, the carbon nanocages are not only a good electrochemical platform for electrochemical energy storage applications, but also a new functional support material for electrocatalytic energy conversion applications.

At present, important progress has been made in the controllable preparation and electrochemical energy‐oriented functionalization of carbon‐based nanocages and their composites. The obtained carbon‐based nanocages (including graphene‐like carbon nanocages, cube carbon nanocages, MOFs‐based polyhedral carbon nanocages, and hollow porous carbon spheres) have the advantages of high surface area, large pore volume, good graphitization degree, abundant surface defects, and easy doping and modulation, which showed excellent performance in many electrochemical fields. On this basis, by taking advantage of the electronic structure of carbon nanocages and the coordination or anchoring effect of heteroatoms on active components, people have successfully constructed carbon‐based composite nanocages with metal nanostructures or metal single atom structures for different electrochemical applications (including catalytic and noncatalytic materials). In short, among many carbon nanomaterials (such as carbon nanotubes, graphene nanosheets, carbon black, etc.), carbon nanocages have unique hollow structure and controllable dimension and pore structure, showing bright prospects and fascinating opportunities in the field of electrochemical materials.

Compared with other carbon nanomaterials, several unique structural properties and geometrical advantages of carbon nanocages make them particularly attractive in practical applications and endow with enhancement effects for electrochemical applications, including electrochemical energy storage and electrocatalysis. i) The porous shell (micropore or mesopore shells) with nanoscale thickness can facilitate the electrolyte to easily enter the nanocages, strengthen the contact between the electrode and the electrolyte, and shorten the ion diffusion path. ii) The hollow interior structures of carbon nanocages are high‐efficiency nanoreactors or mass storage containers that can be used as electrolyte ion storage, providing additional space and sufficient electrolyte ions for rapid electrochemical dynamic processes. iii) The porous shells and hollow structures can also efficiently mitigate volume changes in electrochemical processes, especially in electrochemical storage applications (e.g., as sulfur storage containers and buffer space to accommodate volume expansion of charge and discharge), contributing to greatly improved cycling stability. In addition, the enhanced electrical conductivity (e.g., graphitized carbon nanocages), abundant contactable surface area (e.g., micro/mesoporous amorphous carbon nanocages), various nanocages morphology (especially spherical nanocages with high packing density and high machining performance) and adjustable heterogeneous doping or composite structures (such as carbon nanocages/metal single atoms, carbon nanocages/metal nanomaterials composite structures) compensate for the shortcomings of single‐size nanostructures and single component of carbon nanocages.

**Figure 30:**
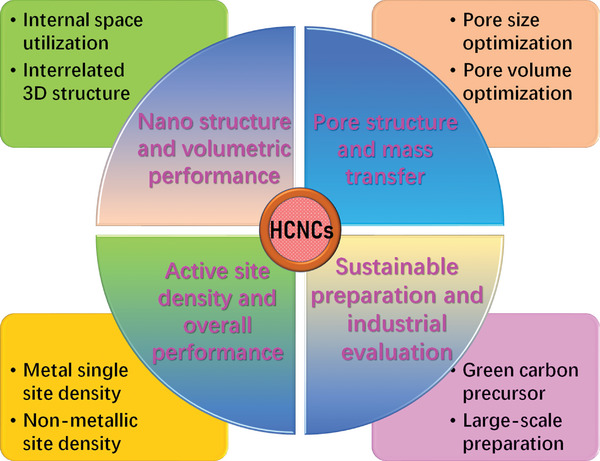
Challenges and strategies of hollow carbon nanocages (HCNCs) for electrochemical applications.

Despite these advantages, the application of carbon nanocages in electrochemical applications still faces some challenges. In high volume specific energy and high‐power density applications of advanced electrochemical systems (such as storage‐type battery and conversion‐type battery), the hollow carbon nanocages often face several challenges as follows: 1) the nanostructure and volumetric performance, 2) pore structure and mass transfer, 3) active site density and overall performance, and (4) sustainable preparation and industrial evaluation (see **Figure** **30** for details).

**Figure 31:**
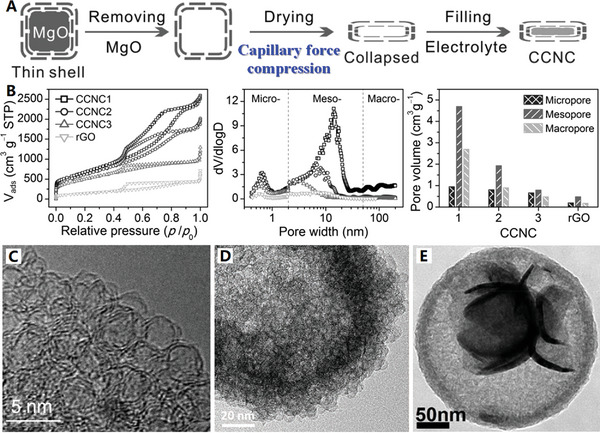
A,B) Collapse carbon nanocages prepared by capillary force compression with hierarchical porous structure. Reproduced with permission.^[^ ^26^ ^]^ Copyright 2017, Wiley. C) Dense and small size carbon nanocages 3D network. Reproduced with permission.^[^ ^124^ ^]^ Copyright 2017, Elsevier. D) Small carbon nanocages self‐assembled hollow microspheres. Reproduced with permission.^[^ ^126^ ^]^ Copyright 2020, Wiley. E) “Ship‐in‐bottle” nanostructures of carbon nanocages. Reproduced with permission.^[^ ^158^ ^]^ Copyright 2017, ACS.

**Figure 32:**
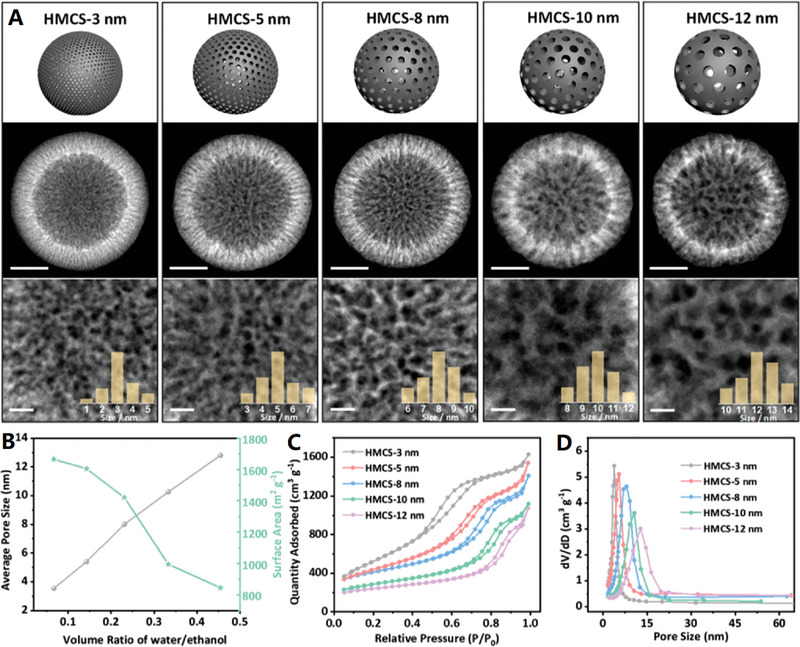
Mesoporous carbon spheres with different porosity (pore size 3, 5, 8, 10, 12 nm). Reproduced with permission.^[^ ^218^ ^]^ Copyright 2022, ACS.

1) The nanostructure and volumetric performance: the internal space of the carbon nanocages is too big (>100 nm), leading to low space utilization and insufficient volumetric performances. Therefore, it is necessary to further optimize the nanostructure of carbon nanocages (constructing collapsed hollow carbon nanocages (reduce excess macropores and mesoporous pores) (see **Figure** **31**A,B),^[^ ^26^ ^]^ dense and small size (≈5 nm) carbon nanocages 3D network structures (see Figure 31C) (or small carbon nanocages self‐assembled hollow microspheres (see Figure 31D)^[^ ^124^ ^]^) and “ship‐in‐bottle” nanostructures (see Figure 31E)^[^ ^126^ ^]^) to increase the internal material utilization and improve the volume performances. In particular, the collapse carbon nanocages (CCNs) prepared by capillary force compression can effectively reduce the excess macropores and mesoporous pores, which is a very effective method to improve the volume energy density, while still maintain a high power density. The optimal CCNs have unique hierarchical porous structure, extremely high electrode density (1.32 g cm^−3^), and excellent volumetric capacitance (233 F cm^−3^). The CCNs have high volume energy density (73 Wh L^−1^), high power density, and excellent stability in ionic liquid.^[^ ^158^ ^]^
2) Pore structure and mass transfer: at present, many carbon nanocages are limited to small micropore (<1 nm) structure (micropores are often formed spontaneously during pyrolysis^[^ ^26^ ^]^), which greatly limits the internal utilization and solution diffusion of carbon nanocages, and ultimately affects the dynamics. Therefore, in order to achieve effective diffusion of electrolyte ions and resultant kinetic optimization, it is necessary to design good nanostructures and rational porosity (hierarchical porous structure) at the same time.^[^ ^217^ ^]^ Particularly, the optimization of pore size and pore volume of carbon nanocages (increasing the proportion of smaller mesopores (e.g., 2–15 nm)) can improve the mass transfer rate and optimize the electrochemical kinetics. Very recently, Wang and co‐workers have investigated the effect of porosity engineering on catalytic performances (i.e., mass transfer enhanced electrochemical performance at mesoscopic scale) by using a porous material platform consisting of a series of HMCSs samples (pore size 3, 5, 8, 10, 12 nm) (see **Figure** **32**).^[^ ^218^ ^]^ Due to its optimal mass transfer, HMCS‐8 nm has the highest electrochemical performance (166 mW cm^−2^) for zinc–air cells (ZACs) and performs well in dual‐function ZACs for wastewater purification (70% RhB degradation after 2 min and 99% degradation after 32 min).
3) Active site density and overall performance: for the metal single‐atom functionalized carbon nanocages, the density of metal sites on the carbon substrate is still too low (<1 metal atom nm^−2^), resulting in the dilemma of insufficient overall catalytic efficiency. Therefore, it is necessary to further increase the density of both metal sites and coordinated nonmetal sites to solve the problems of low area catalytic activity and insufficient overall catalytic efficiency for the electrocatalysis applications.^[^ ^219^ ^]^ Recently, Hai et al. reported a general and scalable two‐step annealing method for the preparation of ultrahigh‐density single‐atom catalysts (UHD‐SACs) on various supports, including nitrogen‐doped carbon (NC). These UHD‐SACs have shown excellent performance in various catalytic applications. For example, the application of UHD Ni_1_/NC in the electrochemical reduction of carbon dioxide shows that the current density on UHD Ni_1_/NC is significantly higher (ten times higher) than that on low‐density Ni_1_/NC, and the high selectivity for CO is maintained.^[^ ^220^ ^]^
4) Sustainable preparation and industrial evaluation: most of the previous reported carbon nanocages do not consider sustainable large‐scale preparation (batch preparation in Kg grade) and industrial (practical technology application) environmental performance evaluation, which are often necessary for the industrial production and practical applications. Therefore, further consideration should be given to sustainable preparation (e.g., developing green carbon precursors and large‐scale preparation technologies) and evaluation of practical industrial applications for some high‐quality carbon nanocages. The carbon precursors require carbon containing substances that are more environmentally friendly and readily available (e.g., bagasse can be used as a green carbon precursor mentioned earlier^[^ ^38^ ^]^), or the smart precursors that integrate metals and carbon (e.g., MOFs^[^ ^221^, ^222^, ^223^, ^224^ ^]^). It is highly commendable that, the MOF (ZIF‐8)‐derived superporous carbon nanocages‐based aerogels with 3D interconnected networks hold great promise for the application in both energy storage and electrocatalysis.^[^ ^225^, ^226^ ^]^ On the other hand, most of the laboratory‐prepared hollow carbon nanocages need further structural optimization and performance improvement before proceeding to the next industrial application.

## 7 Conclusions and Prospects

This review provides a comprehensive review of the preparation methods, structural regulation, and modification strategies of HCNCs, and their application in different areas of electrochemical energy storage and conversion. The carbon nanocages here include amorphous carbon nanocages (cubic or polyhedral carbon nanocages), graphene‐like carbon nanocages, and hollow porous (micro/mesoporous) carbon spheres. Well‐shaped spherical or polyhedral carbon nanocages often require sacrificial hard templates or inherited precursors in preparation process. The abundant mesoporous structure of carbon nanocages requires specific secondary template or subsequent activation treatment. Well crystal structure and high graphitization degree of carbon nanocages induced by metal catalysis can greatly improve the conductivity and stability of materials. The interconnected aggregated state of dense carbon nanocages can provide 3D conductive networks. The ship‐in‐bottle structures of carbon nanocages can provide high internal utilization and versatility of materials. Nonmetal heteroatom doping and metal single/dual atom doping of carbon nanocages can endowed abundance surface active sites and greatly improve the reactivity of materials. These colorful carbon nanocages and their composites are widely used in supercapacitors, lithium‐ion batteries, lithium–sulfur batteries, metal–air batteries, as well as fuel cell electrocatalysis, water splitting electrocatalysis, carbon dioxide reduction electrocatalysis, and other electrocatalysis. The development of novel functional carbon nanocages (such as metal single atoms functionalized carbon nanocages) have provided a new idea and method for improving the energy density, power density, and volume performance of different electrochemical applications and practical devices.

The proposals and prospects of this review include 1) further grasp the law of the preparation of carbon nanocages, reveal the morphology genetic relationship between nanocages product and precursor or template, verify structure–activity relationship with the parameters (cage diameter, specific surface area, wall thickness, doping species, etc.), get a series of new type of carbon nanocage materials, laid a solid foundation for the study of the construction, performance, and regulation mechanism of carbon nanocages. 2) The important role of intrinsic carbon defects in efficient electrochemical energy storage or catalytic reactions should be revealed for designing high‐performance carbon nanocages, where the pure carbon nanocages with abundant defects and their excellent electrochemical activity comparable to that of doped carbon nanocages should be explored experimentally. 3) A series of high‐efficiency (optimized site density) metal single atom or diatomic electrocatalysts should be constructed using carbon‐based nanocages as new carriers to expand and deepen the research in the field of carbon‐based atomic structure electrocatalysts and promote the practical process of electrochemical energy storage or conversion system. 4) The development of some new hollow porous carbon nanomaterials, such as ship‐in‐bottle and ball in ball complex carbon nanocages, mixed type hollow porous carbon nanobowls can greatly increase the volumetric energy density, which will provide a new opportunity for the application of hollow porous carbon nanomaterials. 5) Enhancing the electronic conductivity and ionic conductivity of HCNCs are of great significance for high‐performance electrochemical energy storage and conversion devices. And it is obvious that different synthesis methods of HCNCs can lead to differences on their electronic conductivity and ionic conductivity. Therefore, it is necessary to optimize the graphitized structure and the porous structure to balance the relationship between the electronic conductivity and ionic conductivity in the future construction of high‐performance HCNCs.

## Conflict of Interest

The authors declare no conflict of interest.
